# The role of circadian rest-activity rhythm for the link between 25-hydroxyvitamin D and type 2 diabetes: a cohort study

**DOI:** 10.1038/s41387-025-00395-6

**Published:** 2025-10-23

**Authors:** Hanzhang Wu, Hongliang Feng, Jiahe Wei, Shuai Wang, Liangkai Chen, Ningjian Wang, Jihui Zhang, Xiao Tan

**Affiliations:** 1 Department of Big Data in Health Science, Zhejiang University School of Public Health and Sir Run Run Shaw Hospital, Zhejiang University School of Medicine, Hangzhou, China; 2 Center for Sleep and Circadian Medicine, The Affiliated Brain Hospital, Guangzhou Medical University, Guangdong, China; 3 Guangdong Engineering Technology Research Center for Translational Medicine of Mental Disorders, Guangdong, China; 4 Department of Nutrition and Food Hygiene, Hubei Key Laboratory of Food Nutrition and Safety, School of Public Health, Tongji Medical College, Huazhong University of Science and Technology, Wuhan, China; 5 Department of Endocrinology and Metabolism, Gongli Hospital of Shanghai Pudong New Area, Shanghai University of Medicine & Health Sciences, Shanghai, China; 6 Institute and Department of Endocrinology and Metabolism, Shanghai Ninth People’s Hospital, Shanghai Jiao Tong University School of Medicine, Shanghai, China; 7 Department of Psychiatry, Faculty of Medicine, The Chinese University of Hong Kong, Hong Kong, China; 8 Department of Medical Sciences, Uppsala University, Uppsala, Sweden

**Keywords:** Type 2 diabetes, Risk factors

## Abstract

**Background:**

Temporal distribution and amplitude of physical activity/inactivity in 24 h known as circadian rest-activity rhythm may predict the risk of various metabolic diseases, including type 2 diabetes (T2D), yet the mechanisms behind the diurnal behavior patterns remain largely unexplored.

**Methods:**

This study included 74,165 UK Biobank participants who were free of T2D at baseline. Circadian rest-activity rhythm (CRAR) characteristics, including amplitude (strength), acrophase (timing of peak activity), pseudo-F (robustness), and mesor (height), were assessed using an extended cosine model applied to accelerometer data. T2D was assessed using the established UK Biobank algorithms. Using Cox regression and restricted cubic spline models, we examined the association between CRAR and incident T2D as well as subsequent all-cause mortality among individuals developed T2D during the follow-up. Mediation analysis explored the mediating effects of blood and metabolic biomarkers.

**Results:**

During a median follow-up of 7.9 years, 1784 T2D cases were documented. We found that CRAR abnormalities was associated with a higher risk of incident T2D compared to optimal CRAR, and the multivariate adjusted hazard ratios (HRs) (95% CI) were 1.48 (1.31, 1.67) for low amplitude, 1.25 (1.07, 1.45) for delayed acrophase, 1.17 (1.04, 1.31) for pseudo-F, and 1.55 (1.38, 1.74) for low mesor. Furthermore, low amplitude and low mesor were associated with higher all-cause mortality following the diagnosis of T2D. Serum vitamin D emerged as a crucial mediator in the association between CRAR abnormalities and the risk of T2D as well as subsequent all-cause mortality.

**Conclusion:**

Our study suggests that CRAR abnormalities are linked to an elevated risk of incident T2D and subsequent mortality. These associations are mediated by blood and metabolic biomarkers, with serum vitamin D playing a significant role as the primary mediator.

## Introduction

Diabetes remains a significant global public health issue, affecting nearly 537 million adults in 2021, with projections suggesting this number could rise to 578 million by 2030 [1, 2]. Type 2 diabetes (T2D) accounts for approximately 90% of all diabetes cases. The rising prevalence of T2D can partly be attributed to lifestyle risk factors, including physical inactivity, poor diet, as well as insufficient sleep and disrupted circadian patterns (e.g. shift work) [3, 4].

Circadian rhythms regulate our daily behavioral and physiological cycles to align with the 24-h light-dark cycle. These rhythms are regulated by a master clock in the suprachiasmatic nuclei of the hypothalamus, which is crucial for coordinating daily sleep and wakefulness cycles, along with various metabolic functions including eating patterns, tissue metabolism, and hormone secretion [5–7]. The circadian clock autonomously regulates daily fluctuations in baseline glucose levels and glucose tolerance [8]. Experimental studies in humans have found that circadian misalignment can impair glucose regulation and increase inflammation, regardless of sleep loss [9]. Epidemiological studies have shown that night shift work, especially rotating shifts that include nights, is associated with a 15–44% increased risk of diabetes [4]. Additionally, our previous research has demonstrated that carrying the MTNR1B G risk allele, along with a late chronotype, increases the risk of T2D [10]. However, much of this research relies on self-reported data concerning chronotype and work schedules, which may introduce biases. A previous study has demonstrated that an imbalanced rest-activity rhythm, as detected by wearable accelerometry, is associated with an increased risk of T2D [11]. Importantly, this association persists even after considering established risk factors for T2D. However, the mechanisms through which objective circadian characteristics are linked to T2D remain elusive. Our study aimed to identify blood and metabolic markers associated with CRAR metrics and evaluate their association with incident T2D risk, along with exploring their potential mediating roles. Additionally, we conducted a supplementary analysis to determine how optimizing CRAR might reduce the risk of premature mortality among participants with T2D.

## Methods

### Study design and population

The UK Biobank is a prospective cohort study that enrolled over half a million participants aged between 40 and 73 years, who were recruited from 2006 to 2010 across 22 assessment centers throughout England, Scotland, and Wales. This extensive study provides a substantial dataset that supports a wide range of research projects aimed at understanding the genetic, lifestyle, and environmental factors that influence health and disease.

We utilized a subsample of 103,712 participants who, between 2013 and 2015, wore an Axivity AX3 accelerometer (Axivity, Newcastle upon Tyne, UK) on their dominant wrist to collect raw accelerometer datasets over a 7-day period. The UK Biobank accelerometer expert working group subsequently processed this data and generated detailed metrics on the participants’ physical activity intensity in 5-second intervals. We excluded participants with unreliable accelerometry data (n = 11,104), including (1) those accelerometer data were flagged as unreliable; (2) those with less than 72 h of data or who failed to provide data for every 1-h period within a 24-h cycle; (3) those with poorly calibrated data; (4) those whose data were recalibrated using previous records from the same device worn by different participants; (5) those with a non-zero count of interrupted recording periods; and (6) with more than 768 data recording errors (defined as Q3 + 1.5×IQR). We further excluded participants with incomplete information on any variables (n = 538), those with prevalent diabetes at baseline (n = 3,248), and those without complete genetic data or not of European descent (n = 14,657). Ultimately, participants were eligible for the analysis. The study workflow is illustrated in Figure S1.

### Assessment of circadian rest-activity rhythm

We employed an enhanced version of the traditional cosine model, widely used in prior research [12, 13], to map the circadian activity rhythm onto the activity data. This adjustment accommodates the squared wave shape often observed in activity data, offering greater flexibility for fitting. It proves especially useful for analyzing diurnal patterns in older populations, which typically deviate from a simple cosine shape [14]. Our analysis focused on four key parameters: (1) amplitude, defined as the peak-to-nadir difference in activity on the fitted curve, where higher values signify stronger rhythms, characterized by more active days and less active nights; (2) acrophase, represents the timing of peak activity on the fitted curve, measured in fractions of an hour (time of day); (3) pseudo-F, assesses the model’s goodness-of-fit and acts as an indicator of overall rhythmicity, with higher values indicating more robust rhythms; (4) mesor, the midline estimating statistic of rhythm, representing the average activity level over the fitted 24-h rest-activity pattern and reflecting the central tendency of the rhythmic variable. The data were analyzed using the ‘ActCR’ R package [15].

### Ascertainment of T2D

We utilized the established UK Biobank algorithms to identify T2D status [16], using hospital inpatient and outpatient records, primary care data, death registration, self-reported medical history and medication, as well as biochemical examination for glycated hemoglobin (if HbA1c ≥ 6.5%). The admissions and diagnoses data of the records were used to ascertain incident T2D using the ICD-10 code E11. The time-to-event was calculated from the accelerometer assessment to the date of T2D diagnosis, death, or censorship (31 October 2022 for England, 31 August 2022 for Scotland, and 31 May 2022 for Wales), whichever came first. For assessing the association between CARA and all-cause mortality among participants with T2D, person-time was calculated from accelerometer assessment to the occurrence of study outcomes or the end of follow-up (30 November 2022), whichever came first.

### Genotype information

The methodology for genotyping, imputation, and quality control of genetic data in the UK Biobank has been detailed elsewhere [17]. We computed the polygenic risk score (PRS) for T2D using 424 single nucleotide polymorphisms (SNPs) linked to an increased risk of T2D in participants of European ancestry [18]. The PRS was determined by the formula PRS = β1×SNP1 + β2×SNP2 + … + βn×SNPn, where βn represents the effect size of each SNP, and SNPn indicates the number of risk alleles present. This PRS was then categorized into low (lowest tertile), intermediate (middle tertile), and high (highest tertile) risk. The SNP MTNR1B rs10830963 on chromosome 11 was among those directly genotyped in the UK Biobank. In the sensitivity analysis, participants were categorized into GG genotype, GC genotype and CC genotype.

### Blood biochemistry

Blood biochemistry data were collected from about 480,000 participants during their recruitment visits (2006–2010) and from about 20,000 participants during repeat assessments approximately five years later. Details on quality control procedures can be found in an open-source document (https://biobank.ndph.ox.ac.uk/showcase/ukb/docs/biomarker_issues.pdf). Blood count data were also collected from the same participants during their first visit, with additional information on hematology analysis available at (https://biobank.ndph.ox.ac.uk/showcase/ukb/docs/haematology.pdf). In the present study, a total of 30 blood biochemistry biomarkers and 31 blood cell counts were incorporated. Baseline plasma samples from around 280,000 randomly selected UK Biobank participants were analyzed using a high-throughput nuclear magnetic resonance (NMR) metabolomics platform. Detailed protocols on sample collection and metabolomic quantification can be found elsewhere [19–21]. For this study, we included a subset of 170 directly measured metabolic biomarkers for subsequent analyses. Metabolites with less than 10% missing were included, and missing data for each metabolite were imputed using half of the minimum measured value. All biomarkers underwent skewness testing. Skewed distributions were log-transformed, and each biomarker was then standardized before statistical analysis. Table S1 provides details on the category and sample size of these blood and metabolic biomarkers.

### Ascertainment of covariates

Age (continuous), sex (female/male), and educational level (≥college university degree or <college or university degree) were assessed using a touch-screen questionnaire. The Townsend Deprivation Index (continuous), history of shift work (yes/no), physical activity (metabolic equivalent of task hours/week), smoking status (never/previous/current), alcohol drinker status (not current/less than three times a week/three or more times a week), and use of antihypertensive and cholesterol medications (yes/no) were also recorded. Body mass index (BMI) was categories as normal/underweight (<25 kg/m^2^), overweight (25≤ to <30 kg/m^2^), and obese (≥30 kg/m^2^). The healthy diet score, ranging from 0 to 5, was calculated based on daily consumption of vegetables (at least four tablespoons), fruits (at least three pieces), and fish (at least twice weekly), as well as the limited intake of unprocessed red meat and processed meat (no more than twice weekly). Higher scores indicate healthier dietary patterns [22]. Higher scores indicate healthier dietary patterns. Additional confounders included the season of accelerometer wear (spring/summer/autumn/winter), sleep efficiency (continuous), sleep duration (<7 h/day, 7–8 h/day, >8 h/day) as recorded by accelerometers, the first 10 principal components of ancestry, and genotype measurement batch were also included as confounding variables.

### Statistical analysis

**Fig. 1:**
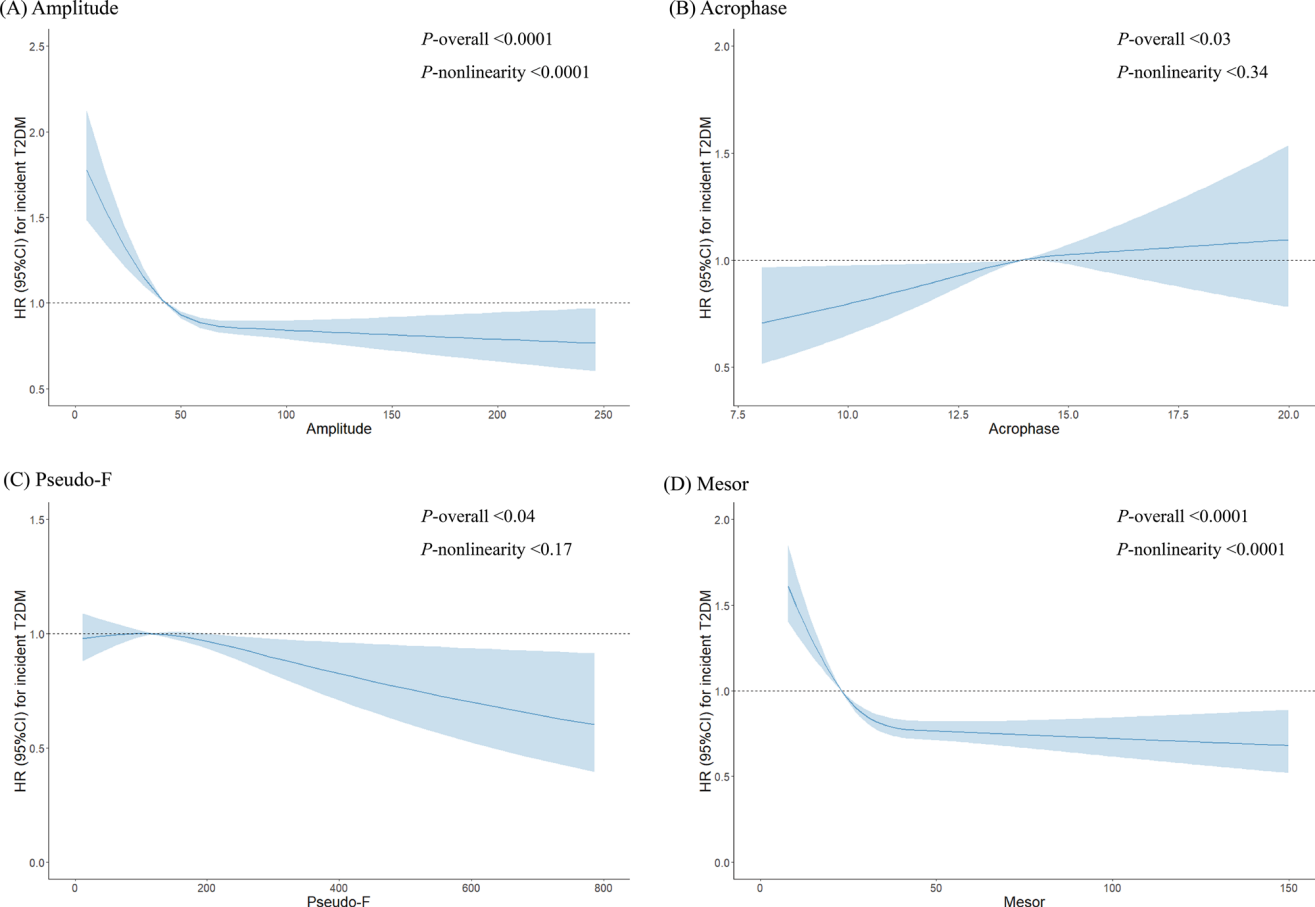
Restricted cubic spline models for the association between 4 subtypes of CRAR metrics and the risk of type 2 diabetes. **A** Amplitude, **B** Acrophase, **C** Pseudo-F, **D** Mesor. The shaded areas represent the 95% confidence intervals of the adjusted hazard ratios (HRs). The restricted cubic spline models are adjusted as per Model 4 in Table 2.

The baseline characteristics of the study participants were presented as means (standard deviation) for continuous variables and as percentages for categorical variables. Following previous methodologies [23, 24], we trichotomized circadian rest-activity metrics to address potential non-linear associations and to mitigate the impact of outliers. Cut-offs were established based on observed change points in the associations with T2D (Fig. 1). For metrics without non-linear associations, cut-offs were chosen to balance the sample sizes across groups. The metrics adjusted were amplitude (categorized as ≤35, >35 to ≤50, >50 counts/min), acrophase (categorized as ≤13:00, >13:00 to ≤15:00, >15:00 hh:mm), pseudo-F (categorized as ≤80, >80 to ≤160, >160), and mesor (categorized as ≤23, >23 to ≤30, >30 counts/min).

Cox proportional hazards regression models were used to estimate hazard ratios (HRs) and 95% confidence intervals (CIs) for the association between CRAR metrics and the risk of T2D and all-cause mortality among participants T2D. The assumption of proportional hazards was examined using Schoenfeld residuals, confirming no violations in our dataset. The model was adjusted for multiple covariates, including age, sex, BMI category, recruitment center, Townsend deprivation index, healthy diet score, education level, alcohol consumption, smoking status, shift work history, physical activity, season of accelerometer usage, sleep efficiency, sleep duration, and the use of antihypertensive and cholesterol medications. Adjustments were also made for the first 10 principal components of ancestry and genotype measurement batch in the genetic analysis. Additionally, to explore the dose-response association, restricted cubic spline (RCS) analyses with three knots (at the 5th, 50th, and 95th percentiles) were performed [25]. Joint analyses using Cox proportional hazards models were conducted to explore the association between CRAR metrics and T2D outcomes across various strata defined by genetic risk for T2D and MTNR1B polymorphisms. To assess potential multiplicative interactions, an interaction term of CRAR and genetic variants was included in the fully adjusted model.

Linear regression analysis was employed to identify blood and metabolic biomarkers associated with the CRAR. Standardized biochemical biomarkers were served as the dependent variables, while CRAR metrics was utilized as the independent variable. Models with adjustment variables identical to those used in Model 3 of Table 2 were utilized in the analysis. A Bonferroni corrected P < 0.000216 (0.05 divided by 231) was considered statistically significant. To assess whether the association between CRAR metrics and risk of T2D was mediated by the identified biochemical biomarkers, we conducted mediation analyses. Moreover, we also use the repeated measurements data of blood biomarkers and Phase 2 NMR metabolic biomarkers to verify our result.

To ensure the robustness of our findings, we conducted several sensitivity analyses. First, we excluded cases of T2D that occurred within the first year of follow-up to evaluate the potential influence of reverse causation bias on our results. Second, we conducted prespecified stratified analyses to explore how age (<65/ ≥ 65 years) and sex (female/male) might modify the effects. Third, we employed multiple imputation by chained equations to handle missing covariate data, rather than excluding these data points. Fourth, we excluded participants with history of shift work. Fifth, we additionally adjusted for mediating factors to examine whether the observed associations were attenuated. Sixth, we further adjusted for the duration of T2D in the analysis of all-cause mortality in the participants with T2D. Finally, we utilized the Fine and Gray models to account for the competing risks posed by deaths.

A two-sided P < 0.05 was considered statistically significant except for the Bonferroni multiple testing. All statistical analyses were conducted using SAS version 9.4 (SAS Institute Inc., Gary, NC, USA) and R software version 4.3.1.

## Results

### Basic characteristics

In the main analysis, a total of 74,157 participants were enrolled in this study and were observed for a median duration of 7.91 years, accumulating a total of 575,767 person-years of follow-up. Table 1 presents the characteristics of the participants categorized by amplitude. Those in the lower amplitude categories tended to be older and obese, possess a university degree, and have a higher Townsend deprivation index. They also exhibited shorter sleep durations and lower sleep efficiency. Furthermore, these participants were less likely to be non-smokers, consumed less alcohol, and had lower scores on the healthy diet index. The baseline characteristics of participants with T2D in the supplementary analysis are presented in Table S2.

**Table 1:** Baseline characteristics of the study participants (N = 74,165) ^a^.

Characteristics		Categories of amplitude			*P value* ^*b*^
	Total	High	Intermediate	Low	
No. of participants	74,165	25,226	26,854	22,085	
Age (years)	56.3 (7.76)	55.3 (7.67)	56.2 (7.67)	57.6 (7.78)	<0.0001
Sex (male, %)	43.4	46.4	37.9	46.6	<0.0001
Body mass index categories					<0.0001
Normal/Underweight (<25 kg/m^2^)	40.4	47.0	42.1	30.6	
Overweight (25–30 kg/m^2^)	41.7	40.3	41.6	43.6	
Obese (≥30 kg/m^2^)	17.9	12.7	16.3	25.8	
Townson depretive index	–1.92 (2.69)	–2.01 (2.64)	–1.98 (2.66)	–1.76 (2.79)	<0.0001
Recruitment regions					0.79
England	89.6	89.6	89.5	89.7	
Wales	6.60	6.63	6.73	6.40	
Scotland	3.80	3.81	3.74	3.86	
Education level (college or higher, %)	41.85	40.9	42.0	42.8	<0.001
PA (MET × hour/week)	42.5 (40.6)	52.1 (45.7)	41.1 (38.5)	32.9 (33.7)	<0.0001
Season of accelerometer wear					<0.001
Spring	22.6	23.5	22.3	21.7	
Summer	26.1	26.3	27.0	24.8	
Autumn	29.8	30.1	29.6	29.9	
Winter	21.5	20.1	21.0	23.6	
Smoking status (%)					<0.001
Current smoker	58.0	58.0	59.2	56.7	
Ex-smoker	35.5	35.9	35.0	35.7	
Non-smoker	6.46	6.16	5.85	7.55	
Alcohol consumption					<0.001
Not current	4.90	4.15	4.82	5.86	
Two or less times a week	44.5	42.6	44.6	46.6	
Three or more times a week	50.6	53.3	50.6	47.5	
Healthy diet score	3.51 (1.14)	3.56 (1.12)	3.56 (1.13)	3.40 (1.15)	<0.0001
Sleep efficiency	0.76 (0.07)	0.77 (0.07)	0.77 (0.07)	0.75 (0.08)	<0.0001
Sleep duration					0.20
< 7 h/day	33.1	32.9	32.9	33.7	
7–8 h/day	46.5	46.9	47.7	44.5	
> 8 h/day	20.4	20.2	19.4	21.9	
Shift work	21.9	23.6	21.0	20.9	<0.0001
Use of blood pressure-lowering medications (yes)	8.79	6.91	8.31	11.5	<0.0001
Use of cholesterol-lowering medications (yes)	12.1	9.52	11.2	16.1	<0.0001

*MET* metabolic equivalent, *PA* physical activity.

^a^Continuous variables are expressed as mean (standard deviation) and categorical variables are expressed as percentages.

^b^Chi-squared was used for categorical variables and one-way analysis of variance for continuous variables.

### T2D and mortality outcomes

Table 2 presents the association between CRAR metrics and the incidence of T2D. For amplitude, individuals in the low amplitude group had a 63% higher risk of developing T2D compared to those in the high amplitude group, as shown in Model 1. This association was attenuated upon adjustment for covariates. In the fully adjusted Model 3, which includes sleep parameter, the association between categories of amplitude and incident T2D remained significant, with the low amplitude group showing a 55% lower risk. Mesor also demonstrated a strong link to T2D (HR: 1.52; 95% CI: 1.35–1.70 for the high mesor group), persisting after adjustment for all covariates. However, only the worst level of acrophase and pseudo-F showed a statistically significant association with incident T2D. The fully adjusted nonlinear model revealed an L-shaped association between both amplitude and mesor and the incidence of T2D (all P for nonlinearity <0.0001) (Fig. 1).

**Table 2:** Association of circadian rest-activity with the risk of T2DM outcomes (N = 74,165) ^a^.

Subgroup	Circadian rest-activity characteristics			*P* for trend ^c^
**Amplitude**	High	Intermediate	Low	
No. of events	408	546	830	
Person years	197,667	209,456	168,644	
Incidence per 1000 PYs	2.06	2.61	4.92	
Model 1	1.00 (reference)	1.18 (1.04, 1.35) ^b^	1.63 (1.44, 1.83)	<0.0001
Model 2	1.00 (reference)	1.16 (1.02, 1.32)	1.50 (1.32, 1.69)	<0.0001
Model 3	1.00 (reference)	1.17 (1.02, 1.33)	1.45 (1.29, 1.64)	<0.0001
Model 4	1.00 (reference)	1.17 (1.03, 1.34)	1.48 (1.31, 1.67)	<0.0001
**Acrophase**	Advanced	Intermediate	Delayed	
No. of events	418	1087	279	
Person years	116,515	376,768	82,484	
Incidence per 1000 PYs	3.59	2.89	3.38	
Model 1	1.00 (reference)	1.00 (0.89, 1.12)	1.18 (1.01, 1.37)	0.07
Model 2	1.00 (reference)	1.06 (0.95, 1.19)	1.19 (1.02, 1.39)	0.03
Model 3	1.00 (reference)	1.08 (0.96, 1.21)	1.20 (1.03, 1.40)	0.02
Model 4	1.00 (reference)	1.10 (0.98, 1.24)	1.25 (1.07, 1.45)	<0.01
**Pseudo-F**	High	Intermediate	Low	
No. of events	541	557	686	
Person years	200,910	178,709	196,148	
Incidence per 1000 PYs	2.69	3.12	3.50	
Model 1	1.00 (reference)	1.07 (0.95, 1.20)	1.17 (1.04, 1.31)	<0.01
Model 2	1.00 (reference)	1.07 (0.95, 1.20)	1.16 (1.04, 1.30)	<0.01
Model 3	1.00 (reference)	1.06 (0.94, 1.19)	1.14 (1.02, 1.28)	0.02
Model 4	1.00 (reference)	1.08 (0.96, 1.22)	1.17 (1.04, 1.31)	<0.01
**Mesor**	High	Intermediate	Low	
No. of events	471	484	829	
Person years	221,716	182,312	171,740	
Incidence per 1000 PYs	2.12	2.65	4.83	
Model 1	1.00 (reference)	1.15 (1.01, 1.31)	1.65 (1.47, 1.85)	<0.0001
Model 2	1.00 (reference)	1.13 (1.00, 1.29)	1.54 (1.37, 1.73)	<0.0001
Model 3	1.00 (reference)	1.13 (1.00, 1.29)	1.52 (1.35, 1.70)	<0.0001
Model 4	1.00 (reference)	1.14 (1.00, 1.30)	1.55 (1.38, 1.74)	<0.0001

Model 1 was adjusted for age, sex, and BMI.

Model 2 was additionally adjusted for recruitment center, smoking status, drinking status, healthy diet score, educational level, Townsend deprivation index, shiftwork, physical activity, season of accelerometer wear, use of blood pressure-lowering medications, and use of cholesterol-lowering medications.

Model 3 was additionally adjusted for sleep efficiency and sleep duration.

Model 4 was additionally adjusted for T2DM-PRS, first 10 principal components of ancestry, and genotype measurement batch.

*BMI* body mass index, *PYs* person-years, *T2DM-PRS* type 2 diabetes mellitus-polygenic risk score.

^a^Obtained by using multivariable Cox regression model.

^b^Hazard ratios (95% confidence interval) (all such values).

^c^P for trend was calculated across quartiles using multivariable Cox regression models.

Further supplementary analysis shown in Fig. S2 indicates that participants with T2D and low amplitude have an increased risk of all-cause mortality, with an HR of 1.30 (95% CI: 1.03–1.65). Similarly, the low mesor group exhibited HR of 1.28 (95% CI: 1.03–1.59) for T2D, compared to the high mesor group in Model 3. However, no significant associations were observed between acrophase, pseudo-F, and all-cause mortality. Moreover, the RCS analysis revealed a significant non-linear trend in the association between both amplitude and mesor and all-cause mortality among participants with T2D (all P for nonlinearity <0.0001).

### T2D genetic risk analysis

**Fig. 2:**
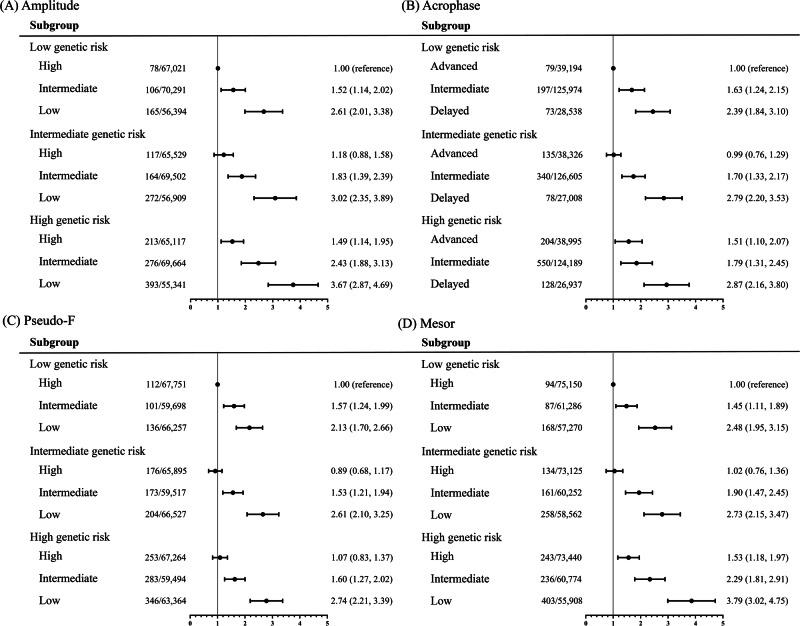
The association between genetic risk and 4 subtypes of CRAR metrics with type 2 diabetes. **A** Amplitude, **B** Acrophase, **C** Pseudo-F, **D** Mesor. All models are adjusted as per Model 4 in Table 2.

We further assessed the joint association of the CRAR parameters and PRS on the outcomes of T2D events. The adjusted cumulative incidences of T2D, stratified by CRAR parameters and genetic risk, are depicted in Fig. 2 and Table S4. Our analysis shows that participants with high genetic risk and the worst CRAR parameters had the highest risk of T2D events. Specifically, participants with high genetic risk and the worst CRAR parameters demonstrated a two- to three-fold increase risk in T2D, with an HR (95% CI) 3.67 (2.87, 4.69) for amplitude, 2.87 (2.16, 3.80) for acrophase, 2.74 (2.21, 3.39) for pseudo-F, and 3.79 (3.02, 4.75) for mesor, compared to those with low genetic risk and optimal CRAR parameters. In stratified analyses by genetic risk, the associations of CRAR parameters and T2D genetic risk were not modified by genetic susceptibility to T2D (all P for interaction >0.15). Additionally, there was no significant interaction between CRAR and the rs10830963 genotype concerning the risk of T2D (all P for interaction >0.41) (Table S5).

### Blood biochemistry analysis

**Fig. 3:**
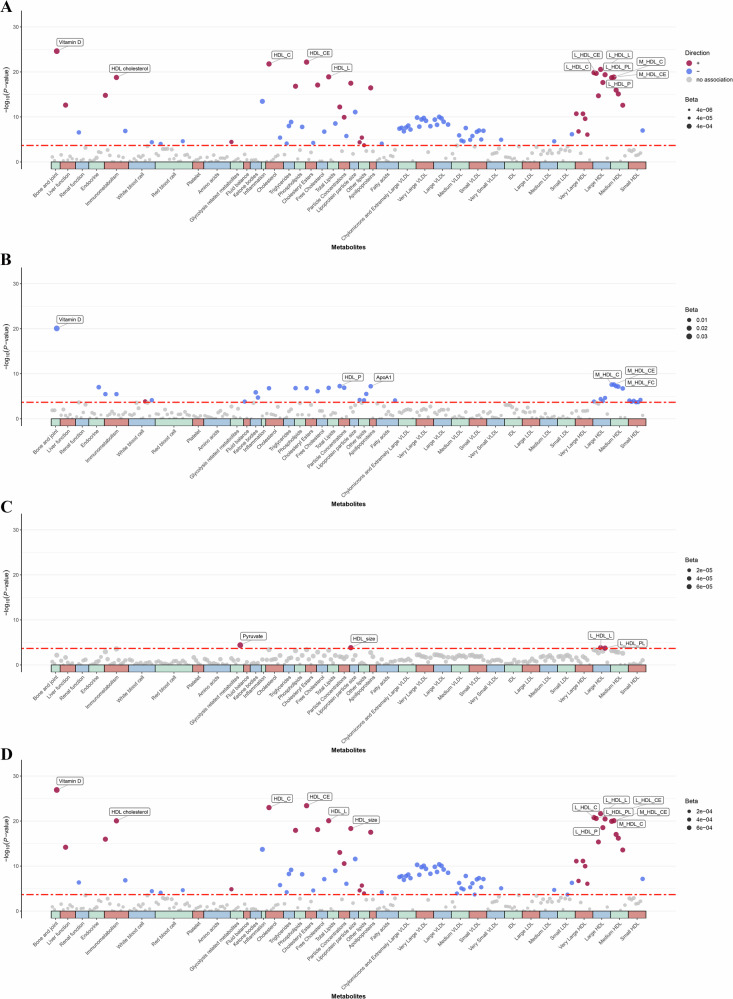
Distinctive association of blood and metabolic biomarker profiles with four subtypes of CRAR metrics. Manhattan plots illustrating the results of linear regression for (**A**) Amplitude, **B** Acrophase, **C** Pseudo-F, and (**D**) Mesor, based on the analysis of 231 biomarkers from 39 categories of blood and metabolic biomarkers. The height of each point represents the negative logarithm of the P value of the t tests, with the color bar indicating the different biomarker categories. The black dashed line indicates the Bonferroni threshold for multiple comparisons (α = 0.05), and text annotations mark the top 15% of biomarkers exhibiting significant differences after Bonferroni correction (P < 0.05/231). All models were adjusted the same as the Model 4 in the Table 2.

The linear regression analyses revealed that out of 231 blood and metabolomic biomarkers examined, 94 exhibited significant associations with amplitude following Bonferroni correction (P < 0.05/231). Among these, 21 were blood biomarkers and 73 were metabolomic biomarkers. Additionally, 44, 11, and 94 blood and metabolomic biomarkers were associated with acrophase, pseudo-F, and mesor, respectively **(**Fig. 3). We observed that Vitamin D exhibited the most pronounced effect size, and it also demonstrated the highest level of statistical significance among all the biomarkers analyzed. For amplitude, the effect size (β) was 6.23 × 10^–4^ with a P = 9.73 × 10^–44^, for acrophase, β = –0.03 with P = 8.66 × 10^–42^; and for mesor, β = 1.28 × 10^–3^ with P = 3.44 × 10^–48^. Additionally, regarding the pseudo-F parameter, both high-density lipoprotein (HDL) cholesterol (β = 7.88 × 10^–5^ with P = 1.19 × 10^–7^) and apolipoprotein A (β = 7.59 × 10^–5^ with P = 8.28 × 10^–7^) demonstrated very strong positive associations, which were also significantly correlated with other CRAR metrics. Besides, other HDL or low-density lipoprotein (LDL) related biomarkers were also exhibited strong correlations with the CRAR metrics.

**Fig. 4:**
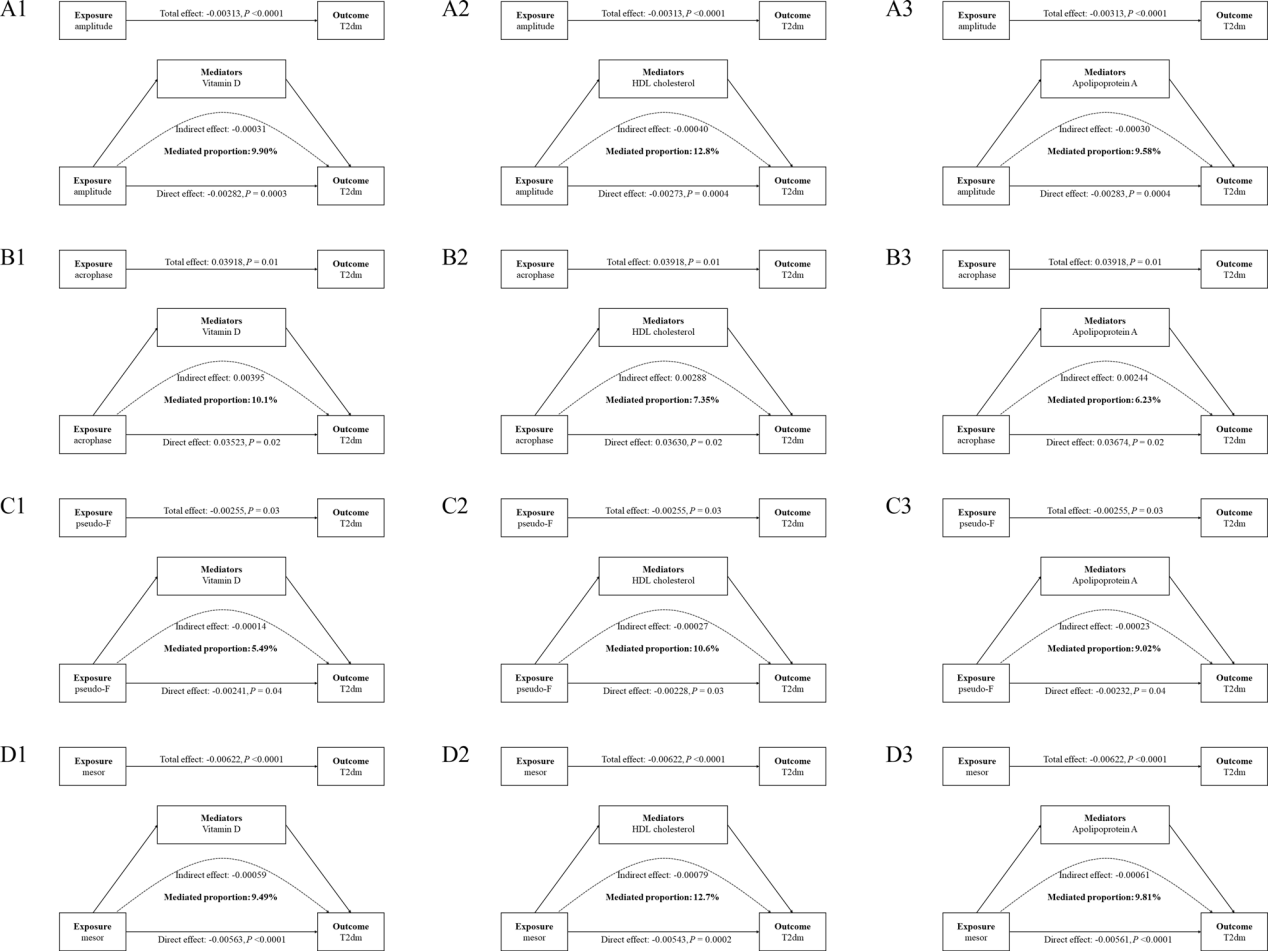
Associations of the four subtypes of CRAR metrics with the risk of type 2 diabetes mediated by three principal biomarkers (Vitamin D, HDL cholesterol, and apolipoprotein A). All models were adjusted the same as the Model 4 in the Table 2. **A1**-**A3** Amplitude, **B1**-**B3** Acrophase, **C1**-**C3** Pseudo-F, **D1**-**D3** Mesor.

Figure 4 illustrates the mediating role of blood and metabolomic biomarkers in the association between CRAR metrics and the risk of T2D. Specifically, we analyzed the mediation effects of three principal biomarkers: Vitamin D, HDL cholesterol, and apolipoprotein A. Vitamin D mediated 9.90% of the association for amplitude, 10.1% for acrophase, 5.49% for pseudo-F, and 9.49% for mesor. HDL cholesterol contributed to 12.8% of the mediation for amplitude, 7.35% for acrophase, 10.6% for pseudo-F, and 12.7% for mesor with respect to the risk of T2D. Apolipoprotein A mediated between 6.23% and 9.81% of the association across all CRAR metrics. In the supplementary analysis, we found that Vitamin D mediated the association between amplitude, mesor, and all-cause mortality among participants with T2D, accounting for 1.49% and 1.33% respectively. No mediating effects were observed for HDL cholesterol and apolipoprotein A between CRAR metrics and all-cause mortality.

In the linear regression analyses of repeated blood and metabolic biomarker assessments, it was found that Vitamin D and other biomarkers remained significantly associated with CRAR metrics (Table S10–13). Additionally, the mediation effects of principal biomarkers revealed that Vitamin D contributed to mediated effects of 1.68% for amplitude, 6.60% for acrophase, and 2.18% for mesor; whereas HDL cholesterol exhibited mediated effects of 4.52% for amplitude and 5.75% for mesor.

### Sensitivity analyses

In sensitivity analyses, the association between CRAR metrics and the risk of T2D remained consistent, even after excluding participants diagnosed with T2D within the first year of follow-up (Table S14). Additionally, stratified analyses by age and sex showed consistent associations across subgroups, with no significant interactions between CRAR metrics and these factors (all P for interaction >0.13) (Table S15, 16). Finally, the associations between CRAR metrics and T2D risk remained consistent across all sensitivity analyses, including multiple imputation, exclusion of shift workers, further adjustment for mediating factors, further adjustment for the duration of T2D, and competing risk models (Tables S17–21).

## Discussion

This large prospective cohort study found that abnormalities in CRAR—characterized by low amplitude, delayed acrophase, low mesor, and low pseudo-F—were associated with an increased risk of T2D. Additionally, low amplitude and low mesor were also associated with higher risks of all-cause mortality among participants with T2D. We found no evidence of interactions between CRAR metrics and T2D genetic risk. It is important to note that CRAR metrics are not only associated with plasma metabolites but also show a strong correlation with vitamin D, serving as a mediator for incident T2D and subsequent all-cause mortality in T2D, which offers insights into the underlying biological mechanisms.

Our findings contribute to the expanding body of literature indicating a significant connection between disrupted circadian rhythms and T2D. Previous cross-sectional study indicated that impaired daily regularity and increased fragmentation of rest-activity rhythms were associated with various metabolic outcomes, including obesity, metabolic syndrome, hypertension, T2D, and dyslipidemia [26]. Several studies have reported that night shift work, which is associated with similar circadian abnormalities [27], is also linked to a higher risk of T2D [4, 28, 29]. Additionally, another study revealed that imbalance rest-activity rhythm parameters (created by non-parametric method), such as lower levels of relative amplitude, shorter periods of the most active continuous 10-h interval, and longer periods of the least active continuous 5-h interval were associated with an increased risk of T2D [11]. Notably, only one study from the Osteoporotic Fractures in Men Study investigated the association between rest-activity rhythm characteristics and glycemic metabolism, which showed that multiple characteristics of rest-activity rhythms were associated with elevated fasting insulin levels and higher insulin resistance at baseline [30].

The biological mechanisms underlying the association between CRAR and the risk of T2D remain unclear. Our study aimed to depict the links between CRAR metrics, blood and metabolic biomarkers, and incident T2D risk. While CRAR metrics showed no significant association with plasma glucose levels, we found a correlation between the acrophase and HbA1c levels. Specifically, a delayed acrophase correlated with elevated HbA1c levels. This observation has been supported by findings from two independent cross-sectional studies, indicating that a later chronotype is linked with poorer glycemic control, including HbA1c levels, in individuals with prediabetes and T2D, regardless of sleep disturbances [31, 32].

Moreover, our results indicate that all four CRAR metrics (low amplitude, delayed acrophase, low mesor, and low pseudo-F) are associated with decreased levels of HDL-associated indicators, which also mediated the association between CRAR metrics and T2D risk. Animal studies suggest that exercising in the morning, during the active phase, may exert a more pronounced metabolic impact compared to nighttime activity during the rest phase, characterized by increased utilization of carbohydrates and ketone bodies, lipid and amino acid degradation [33]. HDL cholesterol is thought to improve insulin resistance by counteracting the effects of LDL cholesterol [34, 35]. Additionally, HDL has been implicated in potentially regulating glucose homeostasis through mechanisms such as insulin secretion, direct glucose uptake by muscle, and enhanced insulin sensitivity [36].

Interestingly, we discovered a robust correlation between CRAR metrics and serum Vitamin D, which also serve as a crucial mediator for the association between CRAR metrics and T2D incidence, as well as subsequent all-cause mortality. The biological plausibility of this association is supported by the endogenous synthesis of serum Vitamin D through skin exposure to ultraviolet B radiation [37]. Accordingly, optimal CRAR may lead to increased sunlight exposure, thereby contributing to higher serum Vitamin D levels. Conversely, unhealthy sleep behaviors such as excessive daytime sleepiness may be associated with reduced outside activities and sun exposure, consequently leading to lower levels of Vitamin D in the human body [38]. Furthermore, the association between higher serum Vitamin D concentrations and a reduced risk of incident T2D is influenced by overall sleep patterns, with daytime sleepiness playing a significant role [39]. Circadian misalignment disrupts endogenous melatonin levels, as studies indicate that morning circadian misalignment consistently delays dim-light melatonin offset [40], suggesting that the internal circadian clock continues to promote sleep and related functions [41]. However, a spill-over of melatonin into the next day may compete with vitamin D3 for binding to the vitamin D receptor [42, 43], potentially diminishing the availability of active vitamin D and hindering its physiological functions. Furthermore, Vitamin D may stimulate insulin release by regulating beta cell intracytoplasmic calcium concentration and activating the exocytosis mechanism, thereby reducing the risk of T2D [44]. Multiple studies have shown that vitamin D serves as a negative regulator of TNF-α and IL-6 release [45], which in turn affects adipose tissue and the immune system [46, 47], ultimately mitigating the T2D risk.

Since shift work is a known extreme form of circadian disruption and may increase the risk of T2D, we conducted additional analyses excluding individuals with a history of shift work to better assess the impact in the general population. The results showed consistent associations, indicating that higher daily rest-activity amplitudes and more optimal timing of rest-activity onset are linked to a lower risk of T2D. We also investigated how genetic predisposition interacts with CRAR metrics regarding the risk of T2D. However, we did not observe any statistically significant interaction between CRAR metrics and either T2D-PRS or polymorphisms in the MTNR1B gene within the study. Nevertheless, throughout the analysis stratified by PRS tertiles or genotyping of MTNR1B, individuals with CRAR abnormalities consistently exhibited a significantly higher risk of T2D. This evidence suggests that enhancing CRAR could provide benefits to individuals, even those with elevated genetic predispositions. From a clinical practice perspective, this study underscores the importance of CRAR in T2D risk. Optimizing CRAR through lifestyle interventions, such as improving sleep hygiene, regulating light exposure, and increasing physical activity, could help reduce T2D risk. These strategies, potentially influencing biomarkers like vitamin D, provide actionable approaches for T2D prevention and management.

The major strengths of this study include objective measurement of CRAR, the prospective and population-based study design, and a series of sensitivity analyses. Additionally, the novelty of our study lies in being the first to utilize biochemical biomarkers to investigate potential biological pathways linking CRAR to the risk of T2D. Nonetheless, the present study has several limitations. First, the CRAR metrics were assessed using a seven-day measurement at baseline, which was not updated during the extended follow-up period and changes in CRAR metrics over time were not captured. Previous evidence indicates that a seven-day monitoring period is commonly employed in activity monitoring studies and typically yields a high level of intra-class correlations in most populations [48]. Second, covariates such as lifestyle factors were not collected at the baseline accelerometer mail-out of the present study but during physical visits to the UK Biobank assessment centers. However, responses generally remained stable over time [49]. Third, while causal relationships cannot be established from this observational study, our mediation analysis offers indirect evidence supporting causality. Moreover, the results remained consistent even after excluding participants with events occurring during the first year of follow-up. Fourth, the study’s participants were exclusively of white ethnicity, potentially restricting the generalizability of the findings to a broader population.

## Conclusion

Accelerometer-measured CRAR abnormalities are linked to the future risk of incident T2D and subsequent all-cause mortality in people with T2D across all levels of genetic risk. Notably, serum vitamin D played a crucial role as a key mediator in this association. These findings underscore the critical role and potential benefits of enhancing CRAR as a strategy to reduce the risk of T2D.

## Supplementary information

Supplemental material

## Data Availability

UK Biobank resource data under application number 55177 was utilized for this research. The UK Biobank data is available on application (www.ukbiobank.ac.uk/).
