# Supplementary material for: The role of circadian rest-activity rhythm for the link between 25-hydroxyvitamin D and type 2 diabetes: a cohort study
Source: Nutr Diabetes. 2025 Oct 23;15:40. doi: 10.1038/s41387-025-00395-6 (PMC12549808; doi:10.1038/s41387-025-00395-6)

| **TABLE S1. Blood and metabolic biomarkers and abbreviations included the UK Biobank** | | | |
| --- | --- | --- | --- |
| **Blood and metabolic biomarkers** | **Type** | **Abbreviations** | **Field ID** |
| White blood cell (leukocyte) count | White blood cell | N/A | 30000 |
| Red blood cell (erythrocyte) count | Red blood cell | N/A | 30010 |
| Haemoglobin concentration | Red blood cell | N/A | 30020 |
| Haematocrit percentage | Red blood cell | N/A | 30030 |
| Mean corpuscular volume | Red blood cell | N/A | 30040 |
| Mean corpuscular haemoglobin | Red blood cell | N/A | 30050 |
| Mean corpuscular haemoglobin concentration | Red blood cell | N/A | 30060 |
| Red blood cell (erythrocyte) distribution width | Red blood cell | N/A | 30070 |
| Platelet count | Platelet | N/A | 30080 |
| Platelet crit | Platelet | N/A | 30090 |
| Mean platelet (thrombocyte) volume | Platelet | N/A | 30100 |
| Platelet distribution width | Platelet | N/A | 30110 |
| Lymphocyte count | White blood cell | N/A | 30120 |
| Monocyte count | White blood cell | N/A | 30130 |
| Neutrophill count | White blood cell | N/A | 30140 |
| Eosinophill count | White blood cell | N/A | 30150 |
| Basophill count | White blood cell | N/A | 30160 |
| Nucleated red blood cell count | Red blood cell | N/A | 30170 |
| Lymphocyte percentage | White blood cell | N/A | 30180 |
| Monocyte percentage | White blood cell | N/A | 30190 |
| Neutrophill percentage | White blood cell | N/A | 30200 |
| Eosinophill percentage | White blood cell | N/A | 30210 |
| Basophill percentage | White blood cell | N/A | 30220 |
| Nucleated red blood cell percentage | Red blood cell | N/A | 30230 |
| Reticulocyte percentage | Red blood cell | N/A | 30240 |
| Reticulocyte count | Red blood cell | N/A | 30250 |
| Mean reticulocyte volume | Red blood cell | N/A | 30260 |
| Mean sphered cell volume | Red blood cell | N/A | 30270 |
| Immature reticulocyte fraction | Red blood cell | N/A | 30280 |
| High light scatter reticulocyte percentage | Red blood cell | N/A | 30290 |
| High light scatter reticulocyte count | Red blood cell | N/A | 30300 |
| Albumin | Liver function | N/A | 30600 |
| Alkaline phosphatase | Bone and joint | N/A | 30610 |
| Alanine aminotransferase | Liver function | N/A | 30620 |
| Apolipoprotein A | Immunometabolism | N/A | 30630 |
| Apolipoprotein B | Immunometabolism | N/A | 30640 |
| Aspartate aminotransferase | Liver function | N/A | 30650 |
| Direct bilirubin | Liver function | N/A | 30660 |
| Urea | Renal function | N/A | 30670 |
| Calcium | Bone and joint | N/A | 30680 |
| Cholesterol | Immunometabolism | N/A | 30690 |
| Creatinine | Renal function | N/A | 30700 |
| C-reactive protein | Immunometabolism | N/A | 30710 |
| Cystatin C | Renal function | N/A | 30720 |
| Gamma glutamyltransferase | Liver function | N/A | 30730 |
| Glucose | Endocrine | N/A | 30740 |
| Glycated haemoglobin (HbA1c) | Immunometabolism | N/A | 30750 |
| HDL cholesterol | Immunometabolism | N/A | 30760 |
| IGF-1 | Endocrine | N/A | 30770 |
| LDL direct | Immunometabolism | N/A | 30780 |
| Lipoprotein A | Endocrine | N/A | 30790 |
| Oestradiol | Endocrine | N/A | 30800 |
| Phosphate | Renal function | N/A | 30810 |
| Rheumatoid factor | Immunometabolism | N/A | 30820 |
| SHBG | Endocrine | N/A | 30830 |
| Total bilirubin | Liver function | N/A | 30840 |
| Testosterone | Endocrine | N/A | 30850 |
| Total protein | Immunometabolism | N/A | 30860 |
| Triglycerides | Immunometabolism | N/A | 30870 |
| Urate | Renal function | N/A | 30880 |
| Vitamin D | Bone and joint | N/A | 30890 |
| Glucose-lactate | Glycolysis related metabolites | Glucose-lactate | 20280 |
| Spectrometer-corrected alanine | Amino acids | Spectrometer-corrected alanine | 20281 |
| Total Cholesterol | Cholesterol | Total_C | 23400 |
| Total Cholesterol Minus HDL-C | Cholesterol | Non_HDL_C | 23401 |
| Remnant Cholesterol (Non-HDL, Non-LDL -Cholesterol) | Cholesterol | Remnant_C | 23402 |
| VLDL Cholesterol | Cholesterol | VLDL Cholesterol | 23403 |
| Clinical LDL Cholesterol | Cholesterol | Clinical_LDL_C | 23404 |
| LDL Cholesterol | Cholesterol | LDL_C | 23405 |
| HDL Cholesterol | Cholesterol | HDL_C | 23406 |
| Total Triglycerides | Triglycerides | Total_TG | 23407 |
| Triglycerides in VLDL | Triglycerides | VLDL_TG | 23408 |
| Triglycerides in LDL | Triglycerides | LDL_TG | 23409 |
| Triglycerides in HDL | Triglycerides | HDL_TG | 23410 |
| Total Phospholipids in Lipoprotein Particles | Phospholipids | Total_PL | 23411 |
| Phospholipids in VLDL | Phospholipids | VLDL_PL | 23412 |
| Phospholipids in LDL | Phospholipids | LDL_PL | 23413 |
| Phospholipids in HDL | Phospholipids | HDL_PL | 23414 |
| Total Esterified Cholesterol | Cholesteryl Esters | Total_CE | 23415 |
| Cholesteryl Esters in VLDL | Cholesteryl Esters | VLDL_CE | 23416 |
| Cholesteryl Esters in LDL | Cholesteryl Esters | LDL_CE | 23417 |
| Cholesteryl Esters in HDL | Cholesteryl Esters | HDL_CE | 23418 |
| Total Free Cholesterol | Free Cholesterol | Total_FC | 23419 |
| Free Cholesterol in VLDL | Free Cholesterol | VLDL_FC | 23420 |
| Free Cholesterol in LDL | Free Cholesterol | LDL_FC | 23421 |
| Free Cholesterol in HDL | Free Cholesterol | HDL_FC | 23422 |
| Total Lipids in Lipoprotein Particles | Total Lipids | Total_L | 23423 |
| Total Lipids in VLDL | Total Lipids | VLDL_L | 23424 |
| Total Lipids in LDL | Total Lipids | LDL_L | 23425 |
| Total Lipids in HDL | Total Lipids | HDL_L | 23426 |
| Total Concentration of Lipoprotein Particles | Lipoprotein Particle Concentrations | Total_P | 23427 |
| Concentration of VLDL Particles | Lipoprotein Particle Concentrations | VLDL_P | 23428 |
| Concentration of LDL Particles | Lipoprotein Particle Concentrations | LDL_P | 23429 |
| Concentration of HDL Particles | Lipoprotein Particle Concentrations | HDL_P | 23430 |
| Average Diameter for VLDL Particles | Lipoprotein Particle Size | VLDL_size | 23431 |
| Average Diameter for LDL Particles | Lipoprotein Particle Size | LDL_size | 23432 |
| Average Diameter for HDL Particles | Lipoprotein Particle Size | HDL_size | 23433 |
| Phosphoglycerides | Other lipids | Phosphoglycerides | 23434 |
| Total Cholines | Other lipids | Cholines | 23436 |
| Phosphatidylcholines | Other lipids | Phosphatidylc | 23437 |
| Sphingomyelins | Other lipids | Sphingomyelins | 23438 |
| Apolipoprotein B | Apolipoproteins | ApoB | 23439 |
| Apolipoprotein A1 | Apolipoproteins | ApoA1 | 23440 |
| Total Fatty Acids | Fatty Acids | Total_FA | 23442 |
| Degree of Unsaturation | Fatty Acids | Unsaturation | 23443 |
| Omega-3 Fatty Acids | Fatty Acids | Omega_3 | 23444 |
| Omega-6 Fatty Acids | Fatty Acids | Omega_6 | 23445 |
| Polyunsaturated Fatty Acids | Fatty Acids | PUFA | 23446 |
| Monounsaturated Fatty Acids | Fatty Acids | MUFA | 23447 |
| Saturated Fatty Acids | Fatty Acids | SFA | 23448 |
| Linoleic Acid | Fatty Acids | LA | 23449 |
| Docosahexaenoic Acid | Fatty Acids | DHA | 23450 |
| Alanine | Amino acids | Alanine | 23460 |
| Glutamine | Amino acids | Glutamine | 23461 |
| Glycine | Amino acids | Glycine | 23462 |
| Histidine | Amino acids | Histidine | 23463 |
| Total Concentration of Branched-Chain Amino Acids (Leucine + Isoleucine + Valine) | Amino acids | Total_BCAA | 23464 |
| Isoleucine | Amino acids | Isoleucine | 23465 |
| Leucine | Amino acids | Leucine | 23466 |
| Valine | Amino acids | Valine | 23467 |
| Phenylalanine | Amino acids | Phenylalanine | 23468 |
| Tyrosine | Amino acids | Tyrosine | 23469 |
| Glucose | Glycolysis related metabolites | Glucose | 23470 |
| Lactate | Glycolysis related metabolites | Lactate | 23471 |
| Pyruvate | Glycolysis related metabolites | Pyruvate | 23472 |
| Citrate | Glycolysis related metabolites | Citrate | 23473 |
| 3-Hydroxybutyrate | Ketone bodies | bOHbutyrate | 23474 |
| Acetate | Ketone bodies | Acetate | 23475 |
| Acetoacetate | Ketone bodies | Acetoacetate | 23476 |
| Acetone | Ketone bodies | Acetone | 23477 |
| Creatinine | Fluid balance | Creatinine | 23478 |
| Albumin | Fluid balance | Albumin | 23479 |
| Glycoprotein Acetyls | Fluid balance | GlycA | 23480 |
| Concentration of Chylomicrons and Extremely Large VLDL Particles | Chylomicrons and Extremely Large VLDL | XXL_VLDL_P | 23481 |
| Total Lipids in Chylomicrons and Extremely Large VLDL | Chylomicrons and Extremely Large VLDL | XXL_VLDL_L | 23482 |
| Phospholipids in Chylomicrons and Extremely Large VLDL | Chylomicrons and Extremely Large VLDL | XXL_VLDL_PL | 23483 |
| Cholesterol in Chylomicrons and Extremely Large VLDL | Chylomicrons and Extremely Large VLDL | XXL_VLDL_C | 23484 |
| Cholesteryl Esters in Chylomicrons and Extremely Large VLDL | Chylomicrons and Extremely Large VLDL | XXL_VLDL_CE | 23485 |
| Free Cholesterol in Chylomicrons and Extremely Large VLDL | Chylomicrons and Extremely Large VLDL | XXL_VLDL_FC | 23486 |
| Triglycerides in Chylomicrons and Extremely Large VLDL | Chylomicrons and Extremely Large VLDL | XXL_VLDL_TG | 23487 |
| Concentration of Very Large VLDL Particles | Very Large VLDL | XL_VLDL_P | 23488 |
| Total Lipids in Very Large VLDL | Very Large VLDL | XL_VLDL_L | 23489 |
| Phospholipids in Very Large VLDL | Very Large VLDL | XL_VLDL_PL | 23490 |
| Cholesterol in Very Large VLDL | Very Large VLDL | XL_VLDL_C | 23491 |
| Cholesteryl Esters in Very Large VLDL | Very Large VLDL | XL_VLDL_CE | 23492 |
| Free Cholesterol in Very Large VLDL | Very Large VLDL | XL_VLDL_FC | 23493 |
| Triglycerides in Very Large VLDL | Very Large VLDL | XL_VLDL_TG | 23494 |
| Concentration of Large VLDL Particles | Large VLDL | L_VLDL_P | 23495 |
| Total Lipids in Large VLDL | Large VLDL | L_VLDL_L | 23496 |
| Phospholipids in Large VLDL | Large VLDL | L_VLDL_PL | 23497 |
| Cholesterol in Large VLDL | Large VLDL | L_VLDL_C | 23498 |
| Cholesteryl Esters in Large VLDL | Large VLDL | L_VLDL_CE | 23499 |
| Free Cholesterol in Large VLDL | Large VLDL | L_VLDL_FC | 23500 |
| Triglycerides in Large VLDL | Large VLDL | L_VLDL_TG | 23501 |
| Concentration of Medium VLDL Particles | Medium VLDL | M_VLDL_P | 23502 |
| Total Lipids in Medium VLDL | Medium VLDL | M_VLDL_L | 23503 |
| Phospholipids in Medium VLDL | Medium VLDL | M_VLDL_PL | 23504 |
| Cholesterol in Medium VLDL | Medium VLDL | M_VLDL_C | 23505 |
| Cholesteryl Esters in Medium VLDL | Medium VLDL | M_VLDL_CE | 23506 |
| Free Cholesterol in Medium VLDL | Medium VLDL | M_VLDL_FC | 23507 |
| Triglycerides in Medium VLDL | Medium VLDL | M_VLDL_TG | 23508 |
| Concentration of Small VLDL Particles | Small VLDL | S_VLDL_P | 23509 |
| Total Lipids in Small VLDL | Small VLDL | S_VLDL_L | 23510 |
| Phospholipids in Small VLDL | Small VLDL | S_VLDL_PL | 23511 |
| Cholesterol in Small VLDL | Small VLDL | S_VLDL_C | 23512 |
| Cholesteryl Esters in Small VLDL | Small VLDL | S_VLDL_CE | 23513 |
| Free Cholesterol in Small VLDL | Small VLDL | S_VLDL_FC | 23514 |
| Triglycerides in Small VLDL | Small VLDL | S_VLDL_TG | 23515 |
| Concentration of Very Small VLDL Particles | Very Small VLDL | XS_VLDL_P | 23516 |
| Total Lipids in Very Small VLDL | Very Small VLDL | XS_VLDL_L | 23517 |
| Phospholipids in Very Small VLDL | Very Small VLDL | XS_VLDL_PL | 23518 |
| Cholesterol in Very Small VLDL | Very Small VLDL | XS_VLDL_C | 23519 |
| Cholesteryl Esters in Very Small VLDL | Very Small VLDL | XS_VLDL_CE | 23520 |
| Free Cholesterol in Very Small VLDL | Very Small VLDL | XS_VLDL_FC | 23521 |
| Triglycerides in Very Small VLDL | Very Small VLDL | XS_VLDL_TG | 23522 |
| Concentration of IDL Particles | IDL | IDL_P | 23523 |
| Total Lipids in IDL | IDL | IDL_L | 23524 |
| Phospholipids in IDL | IDL | IDL_PL | 23525 |
| Cholesterol in IDL | IDL | IDL_C | 23526 |
| Cholesteryl Esters in IDL | IDL | IDL_CE | 23527 |
| Free Cholesterol in IDL | IDL | IDL_FC | 23528 |
| Triglycerides in IDL | IDL | IDL_TG | 23529 |
| Concentration of Large LDL Particles | Large LDL | L_LDL_P | 23530 |
| Total Lipids in Large LDL | Large LDL | L_LDL_L | 23531 |
| Phospholipids in Large LDL | Large LDL | L_LDL_PL | 23532 |
| Cholesterol in Large LDL | Large LDL | L_LDL_C | 23533 |
| Cholesteryl Esters in Large LDL | Large LDL | L_LDL_CE | 23534 |
| Free Cholesterol in Large LDL | Large LDL | L_LDL_FC | 23535 |
| Triglycerides in Large LDL | Large LDL | L_LDL_TG | 23536 |
| Concentration of Medium LDL Particles | Medium LDL | M_LDL_P | 23537 |
| Total Lipids in Medium LDL | Medium LDL | M_LDL_L | 23538 |
| Phospholipids in Medium LDL | Medium LDL | M_LDL_PL | 23539 |
| Cholesterol in Medium LDL | Medium LDL | M_LDL_C | 23540 |
| Cholesteryl Esters in Medium LDL | Medium LDL | M_LDL_CE | 23541 |
| Free Cholesterol in Medium LDL | Medium LDL | M_LDL_FC | 23542 |
| Triglycerides in Medium LDL | Medium LDL | M_LDL_TG | 23543 |
| Concentration of Small LDL Particles | Small LDL | S_LDL_P | 23544 |
| Total Lipids in Small LDL | Small LDL | S_LDL_L | 23545 |
| Phospholipids in Small LDL | Small LDL | S_LDL_PL | 23546 |
| Cholesterol in Small LDL | Small LDL | S_LDL_C | 23547 |
| Cholesteryl Esters in Small LDL | Small LDL | S_LDL_CE | 23548 |
| Free Cholesterol in Small LDL | Small LDL | S_LDL_FC | 23549 |
| Triglycerides in Small LDL | Small LDL | S_LDL_TG | 23550 |
| Concentration of Very Large HDL Particles | Very Large HDL | XL_HDL_P | 23551 |
| Total Lipids in Very Large HDL | Very Large HDL | XL_HDL_L | 23552 |
| Phospholipids in Very Large HDL | Very Large HDL | XL_HDL_PL | 23553 |
| Cholesterol in Very Large HDL | Very Large HDL | XL_HDL_C | 23554 |
| Cholesteryl Esters in Very Large HDL | Very Large HDL | XL_HDL_CE | 23555 |
| Free Cholesterol in Very Large HDL | Very Large HDL | XL_HDL_FC | 23556 |
| Triglycerides in Very Large HDL | Very Large HDL | XL_HDL_TG | 23557 |
| Concentration of Large HDL Particles | Large HDL | L_HDL_P | 23558 |
| Total Lipids in Large HDL | Large HDL | L_HDL_L | 23559 |
| Phospholipids in Large HDL | Large HDL | L_HDL_PL | 23560 |
| Cholesterol in Large HDL | Large HDL | L_HDL_C | 23561 |
| Cholesteryl Esters in Large HDL | Large HDL | L_HDL_CE | 23562 |
| Free Cholesterol in Large HDL | Large HDL | L_HDL_FC | 23563 |
| Triglycerides in Large HDL | Large HDL | L_HDL_TG | 23564 |
| Concentration of Medium HDL Particles | Medium HDL | M_HDL_P | 23565 |
| Total Lipids in Medium HDL | Medium HDL | M_HDL_L | 23566 |
| Phospholipids in Medium HDL | Medium HDL | M_HDL_PL | 23567 |
| Cholesterol in Medium HDL | Medium HDL | M_HDL_C | 23568 |
| Cholesteryl Esters in Medium HDL | Medium HDL | M_HDL_CE | 23569 |
| Free Cholesterol in Medium HDL | Medium HDL | M_HDL_FC | 23570 |
| Triglycerides in Medium HDL | Medium HDL | M_HDL_TG | 23571 |
| Concentration of Small HDL Particles | Small HDL | S_HDL_P | 23572 |
| Total Lipids in Small HDL | Small HDL | S_HDL_L | 23573 |
| Phospholipids in Small HDL | Small HDL | S_HDL_PL | 23574 |
| Cholesterol in Small HDL | Small HDL | S_HDL_C | 23575 |
| Cholesteryl Esters in Small HDL | Small HDL | S_HDL_CE | 23576 |
| Free Cholesterol in Small HDL | Small HDL | S_HDL_FC | 23577 |
| Triglycerides in Small HDL | Small HDL | S_HDL_TG | 23578 |

| **Table S2. Baseline Characteristics of the Study Participants with T2D ^a^** | | | | | |
| --- | --- | --- | --- | --- | --- |
| **Characteristics** |  | **Categories of amplitude** | | | ***P* value ^b^** |
|  | Total | High | Intermediate | Low |  |
| No. of participants | 4,551 | 949 | 2,263 | 4,551 |  |
| Age (years) | 59.4 (6.81) | 58.6 (6.92) | 58.8 (6.84) | 60.0 (6.68) | <0.0001 |
| Sex (male, %) | 62.4 | 67.7 | 59.1 | 62.1 | <0.001 |
| Body mass index categories |  |  |  |  | <0.0001 |
| Normal/Underweight (<25 kg/m^2^) | 10.6 | 13.8 | 13.2 | 7.8 |  |
| Overweight (25–30 kg/m^2^) | 36.9 | 42.4 | 37.8 | 34.1 |  |
| Obese (≥30 kg/m^2^) | 52.5 | 43.8 | 48.9 | 58.1 |  |
| Townson depretive index | -1.37 (2.94) | -1.47 (2.86) | -1.52 (2.89) | -1.23 (2.99) | <0.01 |
| Recruitment regions |  |  |  |  | 0.50 |
| England | 89.5 | 88.9 | 89.7 | 89.6 |  |
| Wales | 5.49 | 6.01 | 4.72 | 5.73 |  |
| Scotland | 4.99 | 5.06 | 5.56 | 4.63 |  |
| Education level (college or higher, %) | 30.54 | 26.8 | 31.4 | 31.6 | 0.02 |
| PA (MET × hour/week) | 38.0 (41.4) | 47.7 (47.8) | 41.9 (42.8) | 31.8 (36.4) | <0.0001 |
| Season of accelerometer wear |  |  |  |  | 0.03 |
| Spring | 21.5 | 22.9 | 22.2 | 20.6 |  |
| Summer | 26.2 | 25.7 | 27.2 | 25.9 |  |
| Autumn | 30.7 | 31.6 | 30.5 | 30.5 |  |
| Winter | 21.5 | 19.8 | 20.2 | 23.0 |  |
| Smoking status (%) |  |  |  |  | 0.81 |
| Current smoker | 44.0 | 42.4 | 45.5 | 43.9 |  |
| Ex-smoker | 47.0 | 48.1 | 46.6 | 46.8 |  |
| Non-smoker | 8.99 | 9.51 | 7.94 | 9.38 |  |
| Alcohol consumption |  |  |  |  | <0.001 |
| Not current | 8.22 | 6.22 | 7.38 | 9.54 |  |
| Two or less times a week | 52.5 | 47.9 | 53.6 | 53.9 |  |
| Three or more times a week | 39.3 | 45.9 | 39.0 | 36.6 |  |
| Healthy diet score | 3.29 (1.19) | 3.29 (1.18) | 3.37 (1.16) | 3.24 (1.20) | 0.67 |
| Sleep efficiency | 0.74 (0.08) | 0.75 (0.08) | 0.75 (0.08) | 0.73 (0.08) | <0.0001 |
| Sleep duration |  |  |  |  | 0.20 |
| < 7 h/day | 39.5 | 38.8 | 39.8 | 39.6 |  |
| 7–8 h/day | 40.7 | 41.7 | 42.0 | 39.6 |  |
| > 8 h/day | 19.8 | 19.5 | 18.2 | 20.8 |  |
| Shift work | 25.7 | 25.6 | 27.3 | 24.9 | 0.27 |
| Use of blood pressure-lowering medications (yes) | 13.5 | 11.7 | 12.9 | 14.6 | 0.02 |
| Use of cholesterol-lowering medications (yes) | 50.7 | 46.2 | 48.3 | 53.9 | <0.0001 |
| ^a^ Continuous variables are expressed as mean (standard deviation) and categorical variables are expressed as percentages. | | | | | |
| ^b^ Chi-squared was used for categorical variables and one-way analysis of variance for continuous variables. | | | | | |

| **Table S3. Association of circadian rest-activity with the risk of all-cause mortality among participants with T2D ^a^** | | | | |
| --- | --- | --- | --- | --- |
| **Subgroup** | **Circadian rest-activity characteristics** | | | ***P* for trend ^c^** |
| **Amplitude** | High | Intermediate | Low |  |
| No. of events | 94 | 119 | 318 |  |
| Person years | 7,444 | 10,236 | 17,431 |  |
| Incidence per 1000 PYs | 12.6 | 11.6 | 18.2 |  |
| Model 1 | 1.00 (reference) | 0.96 (0.73, 1.26) | 1.36 (1.08, 1.71) | <0.01 |
| Model 2 | 1.00 (reference) | 0.97 (0.74, 1.27) | 1.36 (1.08, 1.72) | <0.01 |
| Model 3 | 1.00 (reference) | 0.97 (0.74, 1.27) | 1.30 (1.03, 1.65) | <0.01 |
| **Acrophase** | Advanced | Intermediate | Delayed |  |
| No. of events | 142 | 291 | 98 |  |
| Person years | 8,199 | 21,114 | 5,798 |  |
| Incidence per 1000 PYs | 17.3 | 13.8 | 16.9 |  |
| Model 1 | 1.00 (reference) | 0.96 (0.78, 1.17) | 1.2 (0.93, 1.56) | 0.25 |
| Model 2 | 1.00 (reference) | 1.00 (0.81, 1.22) | 1.19 (0.92, 1.55) | 0.25 |
| Model 3 | 1.00 (reference) | 1.03 (0.84, 1.26) | 1.25 (0.96, 1.62) | 0.14 |
| **Pseudo-F** | High | Intermediate | Low |  |
| No. of events | 158 | 168 | 205 |  |
| Person years | 10,586 | 10,947 | 13,577 |  |
| Incidence per 1000 PYs | 14.9 | 15.3 | 15.1 |  |
| Model 1 | 1.00 (reference) | 1.01 (0.81, 1.25) | 1.08 (0.87, 1.33) | 0.48 |
| Model 2 | 1.00 (reference) | 1.01 (0.81, 1.26) | 1.07 (0.87, 1.32) | 0.53 |
| Model 3 | 1.00 (reference) | 1.00 (0.80, 1.24) | 1.04 (0.84, 1.28) | 0.70 |
| **Mesor** | High | Intermediate | Low |  |
| No. of events | 117 | 113 | 301 |  |
| Person years | 8,773 | 9,112 | 17,225 |  |
| Incidence per 1000 PYs | 13.3 | 12.4 | 17.5 |  |
| Model 1 | 1.00 (reference) | 0.95 (0.73, 1.23) | 1.29 (1.04, 1.60) | <0.01 |
| Model 2 | 1.00 (reference) | 0.95 (0.73, 1.23) | 1.28 (1.03, 1.59) | <0.01 |
| Model 3 | 1.00 (reference) | 0.95 (0.73, 1.23) | 1.28 (1.03, 1.59) | <0.01 |
| Abbreviations: BMI, body mass index; PYs, person-years, T2D, type 2 diabetes. | | | | |
| ^a^ Obtained by using multivariable Cox regression model. | | | | |
| ^b^ Hazard ratios (95% confidence interval) (all such values). | | | | |
| ^c^ P for trend was calculated across quartiles using multivariable Cox regression models. | | | | |
| Model 1 was adjusted for age, sex, and BMI. | | | | |
| Model 2 was additionally adjusted for recruitment center, smoking status, drinking status, healthy diet score, educational level, Townsend deprivation index, shiftwork, physical activity, season of accelerometer wear, use of blood pressure-lowering medications, and use of cholesterol-lowering medications. | | | | |
| Model 3 was additionally adjusted for sleep efficiency and sleep duration. | | | | |

| **Table S4. Subgroup analysis of association between circadian rest-activity and the risk of T2D by genetic risk (N = 74,165) ^a^** | | | | | |
| --- | --- | --- | --- | --- | --- |
| **Subgroup** | **Circadian rest-activity characteristics** | | | ***P* for trend ^c^** | ***P* for interaction** |
| **Amplitude** | High | Intermediate | Low |  | 0.55 |
| **Low genetic risk** |  |  |  |  |  |
| No. of events | 78 | 106 | 165 |  |  |
| Person years | 67,021 | 70,291 | 56,394 |  |  |
| Incidence per 1000 PYs | 1.16 | 1.51 | 2.93 |  |  |
| Minimally adjusted model | 1.00 (reference) | 1.18 (0.88, 1.59) ^b^ | 1.59 (1.21, 2.09) | <0.001 |  |
| Fully adjusted model | 1.00 (reference) | 1.17 (0.87, 1.57) | 1.40 (1.06, 1.85) | 0.02 |  |
| **Intermediate genetic risk** |  |  |  |  |  |
| No. of events | 117 | 164 | 272 |  |  |
| Person years | 65,529 | 69,502 | 56,909 |  |  |
| Incidence per 1000 PYs | 1.79 | 2.36 | 4.78 |  |  |
| Minimally adjusted model | 1.00 (reference) | 1.21 (0.96, 1.54) | 1.76 (1.41, 2.20) | <0.0001 |  |
| Fully adjusted model | 1.00 (reference) | 1.20 (0.95, 1.53) | 1.59 (1.27, 1.99) | <0.0001 |  |
| **High genetic risk** |  |  |  |  |  |
| No. of events | 213 | 276 | 393 |  |  |
| Person years | 65,117 | 69,664 | 55,341 |  |  |
| Incidence per 1000 PYs | 3.27 | 3.96 | 7.10 |  |  |
| Minimally adjusted model | 1.00 (reference) | 1.18 (0.98, 1.41) | 1.59 (1.34, 1.88) | <0.0001 |  |
| Fully adjusted model | 1.00 (reference) | 1.16 (0.97, 1.39) | 1.44 (1.21, 1.71) | <0.001 |  |
| **Acrophase** | Advanced | Intermediate | Delayed |  | 0.50 |
| **Low genetic risk** |  |  |  |  |  |
| No. of events | 79 | 197 | 73 |  |  |
| Person years | 39,194 | 125,974 | 28,538 |  |  |
| Incidence per 1000 PYs | 2.02 | 1.56 | 2.56 |  |  |
| Minimally adjusted model | 1.00 (reference) | 0.93 (0.72, 1.21) | 1.49 (1.08, 2.05) | 0.03 |  |
| Fully adjusted model | 1.00 (reference) | 0.98 (0.75, 1.27) | 1.48 (1.07, 2.04) | 0.03 |  |
| **Intermediate genetic risk** |  |  |  |  |  |
| No. of events | 135 | 340 | 78 |  |  |
| Person years | 38,326 | 126,605 | 27,008 |  |  |
| Incidence per 1000 PYs | 3.52 | 2.69 | 2.89 |  |  |
| Minimally adjusted model | 1.00 (reference) | 0.99 (0.81, 1.21) | 1.10 (0.83, 1.46) | 0.60 |  |
| Fully adjusted model | 1.00 (reference) | 1.05 (0.85, 1.28) | 1.12 (0.84, 1.49) | 0.44 |  |
| **High genetic risk** |  |  |  |  |  |
| No. of events | 204 | 550 | 128 |  |  |
| Person years | 38,995 | 124,189 | 26,937 |  |  |
| Incidence per 1000 PYs | 5.23 | 4.43 | 4.75 |  |  |
| Minimally adjusted model | 1.00 (reference) | 1.05 (0.89, 1.24) | 1.14 (0.91, 1.43) | 0.26 |  |
| Fully adjusted model | 1.00 (reference) | 1.17 (0.99, 1.37) | 1.20 (0.98, 1.50) | 0.08 |  |
| **Pseudo-F** | High | Intermediate | Low |  | 0.15 |
| **Low genetic risk** |  |  |  |  |  |
| No. of events | 112 | 101 | 136 |  |  |
| Person years | 67,751 | 59,698 | 66,257 |  |  |
| Incidence per 1000 PYs | 1.65 | 1.69 | 2.05 |  |  |
| Minimally adjusted model | 1.00 (reference) | 0.89 (0.68, 1.16) | 1.05 (0.81, 1.35) | 0.52 |  |
| Fully adjusted model | 1.00 (reference) | 0.89 (0.68, 1.16) | 1.05 (0.81, 1.35) | 0.68 |  |
| **Intermediate genetic risk** |  |  |  |  |  |
| No. of events | 176 | 173 | 204 |  |  |
| Person years | 65,895 | 59,517 | 66,527 |  |  |
| Incidence per 1000 PYs | 2.67 | 2.91 | 3.07 |  |  |
| Minimally adjusted model | 1.00 (reference) | 0.99 (0.81, 1.23) | 1.05 (0.86, 1.28) | 0.64 |  |
| Fully adjusted model | 1.00 (reference) | 0.98 (0.80, 1.21) | 1.03 (0.84, 1.27) | 0.75 |  |
| **High genetic risk** |  |  |  |  |  |
| No. of events | 253 | 283 | 346 |  |  |
| Person years | 67,264 | 59,494 | 63,364 |  |  |
| Incidence per 1000 PYs | 3.76 | 4.76 | 5.46 |  |  |
| Minimally adjusted model | 1.00 (reference) | 1.20 (1.02, 1.43) | 1.32 (1.13, 1.56) | <0.001 |  |
| Fully adjusted model | 1.00 (reference) | 1.23 (1.04, 1.46) | 1.29 (1.10, 1.52) | <0.01 |  |
| **Mesor** | High | Intermediate | Low |  | 0.94 |
| **Low genetic risk** |  |  |  |  |  |
| No. of events | 94 | 87 | 168 |  |  |
| Person years | 75,150 | 61,286 | 57,270 |  |  |
| Incidence per 1000 PYs | 1.25 | 1.42 | 2.93 |  |  |
| Minimally adjusted model | 1.00 (reference) | 1.01 (0.75, 1.35) | 1.58 (1.23, 2.05) | <0.001 |  |
| Fully adjusted model | 1.00 (reference) | 0.99 (0.74, 1.33) | 1.44 (1.11, 1.87) | <0.01 |  |
| **Intermediate genetic risk** |  |  |  |  |  |
| No. of events | 134 | 161 | 258 |  |  |
| Person years | 73,125 | 60,252 | 58,562 |  |  |
| Incidence per 1000 PYs | 1.83 | 2.67 | 4.41 |  |  |
| Minimally adjusted model | 1.00 (reference) | 1.32 (1.05, 1.66) | 1.72 (1.39, 2.12) | <0.0001 |  |
| Fully adjusted model | 1.00 (reference) | 1.31 (1.04, 1.65) | 1.58 (1.27, 1.95) | <0.0001 |  |
| **High genetic risk** |  |  |  |  |  |
| No. of events | 243 | 236 | 403 |  |  |
| Person years | 73,440 | 60,774 | 55,908 |  |  |
| Incidence per 1000 PYs | 3.31 | 3.88 | 7.21 |  |  |
| Minimally adjusted model | 1.00 (reference) | 1.12 (0.93, 1.34) | 1.67 (1.43, 1.97) | <0.0001 |  |
| Fully adjusted model | 1.00 (reference) | 1.11 (0.93, 1.33) | 1.57 (1.33, 1.85) | <0.0001 |  |
| Abbreviations: BMI, body mass index; PYs, person-years; T2D, type 2 diabetes mellitus. | | | | | |
| ^a^ Obtained by using multivariable Cox regression model. | | | | | |
| ^b^ Hazard ratios (95% confidence interval) (all such values). | | | | | |
| ^c^ P for trend was calculated across quartiles using multivariable Cox regression models. | | | | | |
| Minimally adjusted model was adjusted for age, sex, and BMI. | | | | | |
| Fully adjusted model was additionally adjusted for recruitment center, smoking status, drinking status, healthy diet score, educational level, Townsend deprivation index, shiftwork, physical activity, season of accelerometer wear, use of blood pressure-lowering medications, use of cholesterol-lowering medications, sleep efficiency, sleep duration, first 10 principal components of ancestry, and genotype measurement batch. | | | | | |

| **Table S5. Subgroup analysis of association between circadian rest-activity and the risk of T2D by rs10830963 genotype (N = 74,165) ^a^** | | | | | |
| --- | --- | --- | --- | --- | --- |
| **Subgroup** | **Circadian rest-activity characteristics** | | | ***P* for trend ^c^** | ***P* for interaction** |
| **Amplitude** | High | Intermediate | Low |  | 0.41 |
| **rs10830963 genotype CC** |  |  |  |  |  |
| No. of events | 200 | 275 | 446 |  |  |
| Person years | 102,720 | 109,934 | 89,676 |  |  |
| Incidence per 1000 PYs | 1.95 | 2.50 | 4.97 |  |  |
| Minimally adjusted model | 1.00 (reference) | 1.21 (1.01, 1.45) ^b^ | 1.77 (1.49, 2.10) | <0.001 |  |
| Fully adjusted model | 1.00 (reference) | 1.16 (0.97, 1.40) | 1.61 (1.36, 1.91) | <0.01 |  |
| **rs10830963 genotype CG** |  |  |  |  |  |
| No. of events | 182 | 234 | 319 |  |  |
| Person years | 80,142 | 83,808 | 66,071 |  |  |
| Incidence per 1000 PYs | 2.27 | 2.79 | 4.83 |  |  |
| Minimally adjusted model | 1.00 (reference) | 1.15 (0.95, 1.40) | 1.43 (1.19, 1.72) | <0.0001 |  |
| Fully adjusted model | 1.00 (reference) | 1.17 (0.97, 1.42) | 1.30 (1.08, 1.57) | <0.01 |  |
| **rs10830963 genotype GG** |  |  |  |  |  |
| No. of events | 26 | 37 | 65 |  |  |
| Person years | 14,805 | 15,715 | 12,897 |  |  |
| Incidence per 1000 PYs | 1.76 | 2.35 | 5.04 |  |  |
| Minimally adjusted model | 1.00 (reference) | 1.23 (0.74, 2.04) | 1.88 (1.18, 2.98) | <0.01 |  |
| Fully adjusted model | 1.00 (reference) | 1.29 (0.77, 2.14) | 1.68 (1.05, 2.70) | 0.03 |  |
| **Acrophase** | Advanced | Intermediate | Delayed |  | 0.95 |
| **rs10830963 genotype CC** |  |  |  |  |  |
| No. of events | 212 | 561 | 148 |  |  |
| Person years | 60,153 | 199,357 | 42,819 |  |  |
| Incidence per 1000 PYs | 3.52 | 2.81 | 3.46 |  |  |
| Minimally adjusted model | 1.00 (reference) | 1.01 (0.86, 1.18) | 1.25 (1.01, 1.55) | 0.07 |  |
| Fully adjusted model | 1.00 (reference) | 1.11 (0.94, 1.30) | 1.32 (1.07, 1.64) | 0.01 |  |
| **rs10830963 genotype CG** |  |  |  |  |  |
| No. of events | 182 | 441 | 112 |  |  |
| Person years | 47,792 | 148,921 | 33,308 |  |  |
| Incidence per 1000 PYs | 3.81 | 2.96 | 3.36 |  |  |
| Minimally adjusted model | 1.00 (reference) | 0.95 (0.80, 1.13) | 1.07 (0.84, 1.36) | 0.73 |  |
| Fully adjusted model | 1.00 (reference) | 1.05 (0.88, 1.26) | 1.16 (0.91, 1.47) | 0.25 |  |
| **rs10830963 genotype GG** |  |  |  |  |  |
| No. of events | 24 | 85 | 19 |  |  |
| Person years | 8,570 | 28,490 | 6,357 |  |  |
| Incidence per 1000 PYs | 2.80 | 2.98 | 2.99 |  |  |
| Minimally adjusted model | 1.00 (reference) | 1.32 (0.84, 2.09) | 1.42 (0.77, 2.61) | 0.22 |  |
| Fully adjusted model | 1.00 (reference) | 1.48 (0.93, 2.37) | 1.37 (0.74, 2.55) | 0.24 |  |
| **Pseudo-F** | High | Intermediate | Low |  | 0.96 |
| **rs10830963 genotype CC** |  |  |  |  |  |
| No. of events | 273 | 285 | 363 |  |  |
| Person years | 104,880 | 94,391 | 103,058 |  |  |
| Incidence per 1000 PYs | 2.60 | 3.02 | 3.52 |  |  |
| Minimally adjusted model | 1.00 (reference) | 1.06 (0.90, 1.25) | 1.19 (1.02, 1.40) | 0.03 |  |
| Fully adjusted model | 1.00 (reference) | 1.10 (0.93, 1.30) | 1.19 (1.02, 1.40) | 0.03 |  |
| **rs10830963 genotype CG** |  |  |  |  |  |
| No. of events | 232 | 233 | 270 |  |  |
| Person years | 80,733 | 70,594 | 78,694 |  |  |
| Incidence per 1000 PYs | 2.87 | 3.30 | 3.43 |  |  |
| Minimally adjusted model | 1.00 (reference) | 1.05 (0.88, 1.26) | 1.09 (0.91, 1.30) | 0.34 |  |
| Fully adjusted model | 1.00 (reference) | 1.05 (0.87, 1.26) | 1.08 (0.91, 1.29) | 0.38 |  |
| **rs10830963 genotype GG** |  |  |  |  |  |
| No. of events | 36 | 39 | 53 |  |  |
| Person years | 15,296 | 13,725 | 14,396 |  |  |
| Incidence per 1000 PYs | 2.35 | 2.84 | 3.68 |  |  |
| Minimally adjusted model | 1.00 (reference) | 1.19 (0.75, 1.87) | 1.48 (0.97, 2.27) | 0.07 |  |
| Fully adjusted model | 1.00 (reference) | 1.11 (0.70, 1.76) | 1.45 (0.94, 2.23) | 0.08 |  |
| **Mesor** | High | Intermediate | Low |  | 0.65 |
| **rs10830963 genotype CC** |  |  |  |  |  |
| No. of events | 229 | 260 | 432 |  |  |
| Person years | 115,658 | 95,655 | 91,016 |  |  |
| Incidence per 1000 PYs | 1.98 | 2.72 | 4.75 |  |  |
| Minimally adjusted model | 1.00 (reference) | 1.27 (1.06, 1.52) | 1.77 (1.50, 2.08) | <0.001 |  |
| Fully adjusted model | 1.00 (reference) | 1.24 (1.04, 1.49) | 1.67 (1.42, 1.97) | <0.01 |  |
| **rs10830963 genotype CG** |  |  |  |  |  |
| No. of events | 210 | 192 | 333 |  |  |
| Person years | 89,687 | 72,428 | 67,906 |  |  |
| Incidence per 1000 PYs | 2.34 | 2.65 | 4.90 |  |  |
| Minimally adjusted model | 1.00 (reference) | 1.03 (0.85, 1.25) | 1.51 (1.26, 1.79) | <0.0001 |  |
| Fully adjusted model | 1.00 (reference) | 1.04 (0.86, 1.27) | 1.41 (1.18, 1.68) | <0.0001 |  |
| **rs10830963 genotype GG** |  |  |  |  |  |
| No. of events | 32 | 32 | 64 |  |  |
| Person years | 16,371 | 14,229 | 12,817 |  |  |
| Incidence per 1000 PYs | 1.95 | 2.25 | 4.99 |  |  |
| Minimally adjusted model | 1.00 (reference) | 1.04 (0.64, 1.71) | 1.80 (1.17, 2.77) | <0.01 |  |
| Fully adjusted model | 1.00 (reference) | 1.07 (0.65, 1.76) | 1.60 (1.04, 2.48) | 0.03 |  |
| Abbreviations: BMI, body mass index; PYs, person-years; T2D, type 2 diabetes mellitus. | | | | | |
| ^a^ Obtained by using multivariable Cox regression model. | | | | | |
| ^b^ Hazard ratios (95% confidence interval) (all such values). | | | | | |
| ^c^ P for trend was calculated across quartiles using multivariable Cox regression models. | | | | | |
| Minimally adjusted model was adjusted for age, sex, and BMI. | | | | | |
| Fully adjusted model was additionally adjusted for recruitment center, smoking status, drinking status, healthy diet score, educational level, Townsend deprivation index, shiftwork, physical activity, season of accelerometer wear, use of blood pressure-lowering medications, use of cholesterol-lowering medications, sleep efficiency, sleep duration, first 10 principal components of ancestry, and genotype measurement batch. | | | | | |

| **Table S6. Associations of blood and metabolomic biomarkers with amplitude.** | | | | | |
| --- | --- | --- | --- | --- | --- |
| **Field ID** | **Beta** | **P Value** | **Sample size** | **Biomarker** | **Group** |
| 20280 | -0.0001359 | 0.0153059 | 49747 | Glucose-lactate | Glycolysis related metabolites |
| 20281 | 7.57E-05 | 0.2069011 | 49747 | Spectrometer-corrected alanine | Amino acids |
| 23400 | 0.00013963 | 0.0089579 | 49747 | Total_C | Cholesterol |
| 23401 | -4.10E-05 | 0.457213 | 49747 | Non_HDL_C | Cholesterol |
| 23402 | -7.95E-05 | 0.1496083 | 49747 | Remnant_C | Cholesterol |
| 23403 | -0.0002478 | 1.22E-05 | 49747 | VLDL Cholesterol | Cholesterol |
| 23404 | 6.59E-06 | 0.9043502 | 49747 | Clinical_LDL_C | Cholesterol |
| 23405 | -7.87E-06 | 0.8881793 | 49747 | LDL_C | Cholesterol |
| 23406 | 0.0005116 | 8.14E-24 | 49747 | HDL_C | Cholesterol |
| 23407 | -0.0003192 | 1.05E-08 | 49747 | Total_TG | Triglycerides |
| 23408 | -0.0003365 | 1.14E-09 | 49747 | VLDL_TG | Triglycerides |
| 23409 | -0.0002251 | 7.26E-05 | 49747 | LDL_TG | Triglycerides |
| 23410 | -0.0001342 | 0.0203849 | 49747 | HDL_TG | Triglycerides |
| 23411 | 0.0001834 | 0.0007908 | 49747 | Total_PL | Phospholipids |
| 23412 | -0.0003121 | 3.30E-08 | 49747 | VLDL_PL | Phospholipids |
| 23413 | -4.78E-05 | 0.3960445 | 49747 | LDL_PL | Phospholipids |
| 23414 | 0.0004551 | 2.81E-18 | 49747 | HDL_PL | Phospholipids |
| 23415 | 0.00016875 | 0.0015383 | 49747 | Total_CE | Cholesteryl Esters |
| 23416 | -0.0002094 | 0.0002055 | 49747 | VLDL_CE | Cholesteryl Esters |
| 23417 | -3.93E-05 | 0.4865212 | 49747 | LDL_CE | Cholesteryl Esters |
| 23418 | 0.00051877 | 3.00E-24 | 49747 | HDL_CE | Cholesteryl Esters |
| 23419 | 5.31E-05 | 0.3273824 | 49747 | Total_FC | Free Cholesterol |
| 23420 | -0.0002839 | 5.81E-07 | 49747 | VLDL_FC | Free Cholesterol |
| 23421 | 5.62E-05 | 0.3089524 | 49747 | LDL_FC | Free Cholesterol |
| 23422 | 0.00045845 | 6.87E-19 | 49747 | HDL_FC | Free Cholesterol |
| 23423 | 3.07E-05 | 0.5829681 | 49747 | Total_L | Total Lipids |
| 23424 | -0.0003293 | 4.61E-09 | 49747 | VLDL_L | Total Lipids |
| 23425 | -2.97E-05 | 0.6002343 | 49747 | LDL_L | Total Lipids |
| 23426 | 0.00047692 | 2.29E-20 | 49747 | HDL_L | Total Lipids |
| 23427 | 0.00037591 | 7.39E-12 | 49747 | Total_P | Particle Concentrations |
| 23428 | -0.0002623 | 4.30E-06 | 49747 | VLDL_P | Particle Concentrations |
| 23429 | -0.0001406 | 0.0123925 | 49747 | LDL_P | Particle Concentrations |
| 23430 | 0.00040865 | 6.85E-14 | 49747 | HDL_P | Particle Concentrations |
| 23431 | -0.0003716 | 3.59E-12 | 49747 | VLDL_size | Lipoprotein particle size |
| 23432 | 0.00016633 | 0.0034594 | 49747 | LDL_size | Lipoprotein particle size |
| 23433 | 0.00045797 | 6.26E-19 | 49747 | HDL_size | Lipoprotein particle size |
| 23434 | 0.00021913 | 7.11E-05 | 49747 | Phosphoglycerides | Other lipids |
| 23436 | 0.00027822 | 4.97E-07 | 49747 | Cholines | Other lipids |
| 23437 | 0.00026535 | 1.05E-06 | 49747 | Phosphatidylc | Other lipids |
| 23438 | 0.00017929 | 0.0008148 | 49747 | Sphingomyelins | Other lipids |
| 23439 | -0.0001377 | 0.0136102 | 49747 | ApoB | Apolipoproteins |
| 23440 | 0.00045773 | 4.58E-18 | 49747 | ApoA1 | Apolipoproteins |
| 23442 | -0.0001009 | 0.0831219 | 49747 | Total_FA | Fatty acids |
| 23443 | 0.00019344 | 0.0005186 | 49747 | Unsaturation | Fatty acids |
| 23444 | -8.02E-05 | 0.1679839 | 49747 | Omega_3 | Fatty acids |
| 23445 | 6.46E-05 | 0.262294 | 49747 | Omega_6 | Fatty acids |
| 23446 | 3.87E-05 | 0.5029514 | 49747 | PUFA | Fatty acids |
| 23447 | -0.0002223 | 8.77E-05 | 49747 | MUFA | Fatty acids |
| 23448 | -0.0001119 | 0.0493313 | 49747 | SFA | Fatty acids |
| 23449 | 8.88E-05 | 0.1192533 | 49747 | LA | Fatty acids |
| 23450 | 5.68E-05 | 0.3197762 | 49747 | DHA | Fatty acids |
| 23460 | 1.03E-05 | 0.8649842 | 49747 | Alanine | Amino acids |
| 23461 | 0.00012396 | 0.038292 | 49747 | Glutamine | Amino acids |
| 23462 | 2.62E-05 | 0.6494573 | 49747 | Glycine | Amino acids |
| 23463 | -1.62E-05 | 0.7892114 | 49747 | Histidine | Amino acids |
| 23464 | -8.73E-05 | 0.1331206 | 49747 | Total_BCAA | Amino acids |
| 23465 | -0.0001339 | 0.0277579 | 49747 | Isoleucine | Amino acids |
| 23466 | -3.82E-05 | 0.5174938 | 49747 | Leucine | Amino acids |
| 23467 | -0.0001137 | 0.0497192 | 49747 | Valine | Amino acids |
| 23468 | 3.08E-05 | 0.5824893 | 49747 | Phenylalanine | Amino acids |
| 23469 | -8.08E-05 | 0.1797583 | 49747 | Tyrosine | Amino acids |
| 23470 | -9.53E-05 | 0.0941713 | 49747 | Glucose | Glycolysis related metabolites |
| 23471 | -8.79E-05 | 0.148583 | 49747 | Lactate | Glycolysis related metabolites |
| 23472 | 8.45E-05 | 0.1774357 | 49747 | Pyruvate | Glycolysis related metabolites |
| 23473 | 0.00023731 | 7.90E-05 | 49747 | Citrate | Glycolysis related metabolites |
| 23474 | -2.71E-06 | 0.9637099 | 49747 | bOHbutyrate | Ketone bodies |
| 23475 | -4.70E-05 | 0.433578 | 49747 | Acetate | Ketone bodies |
| 23476 | -5.47E-05 | 0.3479099 | 49747 | Acetoacetate | Ketone bodies |
| 23477 | 6.34E-05 | 0.2808285 | 49747 | Acetone | Ketone bodies |
| 23478 | -5.80E-05 | 0.3523957 | 49747 | Creatinine | Fluid balance |
| 23479 | 0.00014274 | 0.0161101 | 49747 | Albumin | Fluid balance |
| 23480 | -0.0004243 | 3.53E-14 | 49747 | GlycA | Inflammation |
| 23481 | -0.0003203 | 3.98E-09 | 49747 | XXL_VLDL_P | Chylomicrons and Extremely Large VLDL |
| 23482 | -0.0003122 | 9.44E-09 | 49747 | XXL_VLDL_L | Chylomicrons and Extremely Large VLDL |
| 23483 | -0.0003026 | 2.52E-08 | 49747 | XXL_VLDL_PL | Chylomicrons and Extremely Large VLDL |
| 23484 | -0.0003058 | 2.52E-08 | 49747 | XXL_VLDL_C | Chylomicrons and Extremely Large VLDL |
| 23485 | -0.000305 | 3.14E-08 | 49747 | XXL_VLDL_CE | Chylomicrons and Extremely Large VLDL |
| 23486 | -0.0002939 | 8.27E-08 | 49747 | XXL_VLDL_FC | Chylomicrons and Extremely Large VLDL |
| 23487 | -3.86E-05 | 0.5419816 | 49747 | XXL_VLDL_TG | Chylomicrons and Extremely Large VLDL |
| 23488 | -0.0003354 | 8.27E-10 | 49747 | XL_VLDL_P | Very Large VLDL |
| 23489 | -0.0003498 | 1.61E-10 | 49747 | XL_VLDL_L | Very Large VLDL |
| 23490 | 0.00017153 | 0.0107181 | 49747 | XL_VLDL_PL | Very Large VLDL |
| 23491 | -0.0003518 | 2.44E-10 | 49747 | XL_VLDL_C | Very Large VLDL |
| 23492 | -0.0003302 | 4.28E-08 | 49747 | XL_VLDL_CE | Very Large VLDL |
| 23493 | -0.0002561 | 4.68E-10 | 49747 | XL_VLDL_FC | Very Large VLDL |
| 23494 | -0.0003183 | 7.01E-09 | 49747 | XL_VLDL_TG | Very Large VLDL |
| 23495 | -0.0003337 | 1.51E-09 | 49747 | L_VLDL_P | Large VLDL |
| 23496 | -0.0003485 | 3.30E-10 | 49747 | L_VLDL_L | Large VLDL |
| 23497 | -0.0001016 | 0.1288961 | 49747 | L_VLDL_PL | Large VLDL |
| 23498 | -0.0003442 | 9.80E-10 | 49747 | L_VLDL_C | Large VLDL |
| 23499 | -0.0003251 | 1.34E-08 | 49747 | L_VLDL_CE | Large VLDL |
| 23500 | -0.0003555 | 1.35E-10 | 49747 | L_VLDL_FC | Large VLDL |
| 23501 | -0.0003221 | 6.39E-09 | 49747 | L_VLDL_TG | Large VLDL |
| 23502 | -0.000229 | 5.83E-05 | 49747 | M_VLDL_P | Medium VLDL |
| 23503 | -0.0002623 | 4.15E-06 | 49747 | M_VLDL_L | Medium VLDL |
| 23504 | -0.0002225 | 9.55E-05 | 49747 | M_VLDL_PL | Medium VLDL |
| 23505 | -0.000112 | 0.0452855 | 49747 | M_VLDL_C | Medium VLDL |
| 23506 | -5.48E-05 | 0.3231965 | 49747 | M_VLDL_CE | Medium VLDL |
| 23507 | -0.0001874 | 0.0009825 | 49747 | M_VLDL_FC | Medium VLDL |
| 23508 | -0.0003055 | 5.77E-08 | 49747 | M_VLDL_TG | Medium VLDL |
| 23509 | -0.0002956 | 2.60E-07 | 49747 | S_VLDL_P | Small VLDL |
| 23510 | -0.0002889 | 5.14E-07 | 49747 | S_VLDL_L | Small VLDL |
| 23511 | -0.0002375 | 3.70E-05 | 49747 | S_VLDL_PL | Small VLDL |
| 23512 | -0.0002342 | 4.47E-05 | 49747 | S_VLDL_C | Small VLDL |
| 23513 | -0.0002616 | 5.93E-06 | 49747 | S_VLDL_CE | Small VLDL |
| 23514 | -0.0001823 | 0.0013976 | 49747 | S_VLDL_FC | Small VLDL |
| 23515 | -0.0002996 | 1.24E-07 | 49747 | S_VLDL_TG | Small VLDL |
| 23516 | -0.0001352 | 0.0167065 | 49747 | XS_VLDL_P | Very Small VLDL |
| 23517 | -0.0001296 | 0.0218087 | 49747 | XS_VLDL_L | Very Small VLDL |
| 23518 | -0.0001747 | 0.0023021 | 49747 | XS_VLDL_PL | Very Small VLDL |
| 23519 | -2.86E-05 | 0.5991816 | 49747 | XS_VLDL_C | Very Small VLDL |
| 23520 | 1.04E-05 | 0.846058 | 49747 | XS_VLDL_CE | Very Small VLDL |
| 23521 | -0.0001192 | 0.0339609 | 49747 | XS_VLDL_FC | Very Small VLDL |
| 23522 | -0.0002496 | 1.26E-05 | 49747 | XS_VLDL_TG | Very Small VLDL |
| 23523 | -5.89E-05 | 0.2817372 | 49747 | IDL_P | IDL |
| 23524 | 8.37E-05 | 0.1128502 | 49747 | IDL_L | IDL |
| 23525 | 4.94E-05 | 0.3541006 | 49747 | IDL_PL | IDL |
| 23526 | 0.00011688 | 0.025688 | 49747 | IDL_C | IDL |
| 23527 | 0.00011701 | 0.0259832 | 49747 | IDL_CE | IDL |
| 23528 | 0.00010842 | 0.0409872 | 49747 | IDL_FC | IDL |
| 23529 | -0.0002046 | 0.0003624 | 49747 | IDL_TG | IDL |
| 23530 | -0.0001192 | 0.0338639 | 49747 | L_LDL_P | Large LDL |
| 23531 | 2.83E-05 | 0.6117599 | 49747 | L_LDL_L | Large LDL |
| 23532 | 2.53E-05 | 0.6511959 | 49747 | L_LDL_PL | Large LDL |
| 23533 | 4.76E-05 | 0.392144 | 49747 | L_LDL_C | Large LDL |
| 23534 | 2.69E-05 | 0.6320223 | 49747 | L_LDL_CE | Large LDL |
| 23535 | 9.38E-05 | 0.085951 | 49747 | L_LDL_FC | Large LDL |
| 23536 | -0.0002009 | 0.0004381 | 49747 | L_LDL_TG | Large LDL |
| 23537 | -0.0001555 | 0.0062389 | 49747 | M_LDL_P | Medium LDL |
| 23538 | -0.0001253 | 0.028274 | 49747 | M_LDL_L | Medium LDL |
| 23539 | -0.0001221 | 0.0334789 | 49747 | M_LDL_PL | Medium LDL |
| 23540 | -0.0001118 | 0.0499411 | 49747 | M_LDL_C | Medium LDL |
| 23541 | -0.0001514 | 0.0080372 | 49747 | M_LDL_CE | Medium LDL |
| 23542 | -7.55E-06 | 0.8940171 | 49747 | M_LDL_FC | Medium LDL |
| 23543 | -0.0002335 | 3.37E-05 | 49747 | M_LDL_TG | Medium LDL |
| 23544 | -0.0001842 | 0.0010684 | 49747 | S_LDL_P | Small LDL |
| 23545 | -0.0001512 | 0.0077337 | 49747 | S_LDL_L | Small LDL |
| 23546 | -0.0001463 | 0.0092672 | 49747 | S_LDL_PL | Small LDL |
| 23547 | -0.0001279 | 0.0233729 | 49747 | S_LDL_C | Small LDL |
| 23548 | -0.0001582 | 0.0053255 | 49747 | S_LDL_CE | Small LDL |
| 23549 | -2.01E-05 | 0.6984546 | 49747 | S_LDL_FC | Small LDL |
| 23550 | -0.0002773 | 5.69E-07 | 49747 | S_LDL_TG | Small LDL |
| 23551 | 0.00035132 | 5.44E-11 | 49747 | XL_HDL_P | Very Large HDL |
| 23552 | 0.00036466 | 4.82E-12 | 49747 | XL_HDL_L | Very Large HDL |
| 23553 | 0.00028763 | 1.29E-07 | 49747 | XL_HDL_PL | Very Large HDL |
| 23554 | 0.00036117 | 4.78E-12 | 49747 | XL_HDL_C | Very Large HDL |
| 23555 | 0.000309 | 4.18E-08 | 49747 | XL_HDL_CE | Very Large HDL |
| 23556 | 0.00017246 | 0.0075107 | 49747 | XL_HDL_FC | Very Large HDL |
| 23557 | -5.96E-05 | 0.3527876 | 49747 | XL_HDL_TG | Very Large HDL |
| 23558 | 0.00046657 | 2.11E-19 | 49747 | L_HDL_P | Large HDL |
| 23559 | 0.00048712 | 4.36E-22 | 49747 | L_HDL_L | Large HDL |
| 23560 | 0.00048004 | 6.74E-21 | 49747 | L_HDL_PL | Large HDL |
| 23561 | 0.00048244 | 1.39E-21 | 49747 | L_HDL_C | Large HDL |
| 23562 | 0.00048439 | 1.75E-21 | 49747 | L_HDL_CE | Large HDL |
| 23563 | 0.00041728 | 1.19E-16 | 49747 | L_HDL_FC | Large HDL |
| 23564 | 9.10E-05 | 0.139942 | 49747 | L_HDL_TG | Large HDL |
| 23565 | 4.37E-05 | 0.5559457 | 49747 | M_HDL_P | Medium HDL |
| 23566 | 0.00043819 | 1.92E-16 | 49747 | M_HDL_L | Medium HDL |
| 23567 | 0.00040024 | 1.11E-13 | 49747 | M_HDL_PL | Medium HDL |
| 23568 | 0.00048708 | 2.79E-20 | 49747 | M_HDL_C | Medium HDL |
| 23569 | 0.00048858 | 2.51E-20 | 49747 | M_HDL_CE | Medium HDL |
| 23570 | 0.00044964 | 2.02E-17 | 49747 | M_HDL_FC | Medium HDL |
| 23571 | -0.0001229 | 0.0341325 | 49747 | M_HDL_TG | Medium HDL |
| 23572 | 0.00014469 | 0.0140156 | 49747 | S_HDL_P | Small HDL |
| 23573 | 0.00013044 | 0.0245244 | 49747 | S_HDL_L | Small HDL |
| 23574 | 0.00015446 | 0.0071227 | 49747 | S_HDL_PL | Small HDL |
| 23575 | 0.00019231 | 0.0010747 | 49747 | S_HDL_C | Small HDL |
| 23576 | 0.00018231 | 0.0021605 | 49747 | S_HDL_CE | Small HDL |
| 23577 | 0.00018137 | 0.0016485 | 49747 | S_HDL_FC | Small HDL |
| 23578 | -0.0002976 | 7.77E-08 | 49747 | S_HDL_TG | Small HDL |
| 30000 | -0.0002652 | 2.02E-09 | 88326 | White blood cell (leukocyte) count | White blood cell |
| 30010 | -0.0001894 | 9.36E-07 | 88328 | Red blood cell (erythrocyte) count | Red blood cell |
| 30020 | -8.07E-05 | 0.0250285 | 88327 | Haemoglobin concentration | Red blood cell |
| 30030 | -8.35E-05 | 0.0244652 | 88328 | Haematocrit percentage | Red blood cell |
| 30040 | 0.00019451 | 6.51E-06 | 88328 | Mean corpuscular volume | Red blood cell |
| 30050 | 0.00016757 | 8.97E-05 | 88326 | Mean corpuscular haemoglobin | Red blood cell |
| 30060 | 1.33E-05 | 0.7750242 | 88326 | Mean corpuscular haemoglobin concentration | Red blood cell |
| 30070 | -9.31E-05 | 0.0371181 | 88328 | Red blood cell (erythrocyte) distribution width | Red blood cell |
| 30080 | -9.57E-05 | 0.0305049 | 88328 | Platelet count | Platelet |
| 30090 | -9.97E-05 | 0.0223072 | 88327 | Platelet crit | Platelet |
| 30100 | 3.89E-05 | 0.4064365 | 88327 | Mean platelet (thrombocyte) volume | Platelet |
| 30110 | -5.42E-05 | 0.2421389 | 88327 | Platelet distribution width | Platelet |
| 30120 | -0.0001039 | 0.0199211 | 88148 | Lymphocyte count | White blood cell |
| 30130 | -0.0001858 | 1.97E-05 | 88148 | Monocyte count | White blood cell |
| 30140 | -0.000238 | 1.13E-07 | 88148 | Neutrophill count | White blood cell |
| 30150 | -0.0001174 | 0.0103758 | 88148 | Eosinophill count | White blood cell |
| 30160 | -4.52E-05 | 0.3109337 | 88148 | Basophill count | White blood cell |
| 30170 | -3.84E-05 | 0.3795985 | 88147 | Nucleated red blood cell count | Red blood cell |
| 30180 | 0.00013693 | 0.0026641 | 88150 | Lymphocyte percentage | White blood cell |
| 30190 | -1.34E-05 | 0.763685 | 88150 | Monocyte percentage | White blood cell |
| 30200 | -7.70E-05 | 0.1304003 | 88150 | Neutrophill percentage | White blood cell |
| 30210 | -5.54E-05 | 0.237771 | 88150 | Eosinophill percentage | White blood cell |
| 30220 | -2.50E-07 | 0.9956209 | 88150 | Basophill percentage | White blood cell |
| 30230 | -2.83E-05 | 0.5042526 | 88146 | Nucleated red blood cell percentage | Red blood cell |
| 30240 | -9.82E-05 | 0.0143914 | 86572 | Reticulocyte percentage | Red blood cell |
| 30250 | -0.0001346 | 0.0014155 | 86572 | Reticulocyte count | Red blood cell |
| 30260 | 0.00010511 | 0.0232096 | 86572 | Mean reticulocyte volume | Red blood cell |
| 30270 | 0.00018928 | 2.83E-05 | 86572 | Mean sphered cell volume | Red blood cell |
| 30280 | -0.0002223 | 7.28E-07 | 86572 | Immature reticulocyte fraction | Red blood cell |
| 30290 | -0.0001318 | 3.75E-06 | 86572 | High light scatter reticulocyte percentage | Red blood cell |
| 30300 | -0.0002243 | 6.86E-08 | 86572 | High light scatter reticulocyte count | Red blood cell |
| 30600 | 9.45E-05 | 0.0467232 | 79398 | Albumin | Liver function |
| 30610 | -0.0001326 | 0.0013078 | 86722 | Alkaline phosphatase | Bone and joint |
| 30620 | -0.0001025 | 0.0121543 | 86702 | Alanine aminotransferase | Liver function |
| 30630 | 0.00050681 | 7.32E-34 | 78914 | Apolipoprotein A | Immunometabolism |
| 30640 | -0.000189 | 1.01E-05 | 86362 | Apolipoprotein B | Immunometabolism |
| 30650 | 0.00038765 | 7.14E-20 | 86422 | Aspartate aminotransferase | Liver function |
| 30660 | 0.00013547 | 0.0023277 | 74354 | Direct bilirubin | Liver function |
| 30670 | 0.00024125 | 8.02E-09 | 86666 | Urea | Renal function |
| 30680 | 9.05E-05 | 0.0570111 | 79386 | Calcium | Bone and joint |
| 30690 | 1.72E-05 | 0.6758917 | 86722 | Cholesterol | Immunometabolism |
| 30700 | -3.23E-05 | 0.3114425 | 86682 | Creatinine | Renal function |
| 30710 | -0.0001541 | 0.0001807 | 86541 | C-reactive protein | Immunometabolism |
| 30720 | -0.0002206 | 7.12E-11 | 86717 | Cystatin C | Renal function |
| 30730 | -6.43E-05 | 0.0912259 | 86691 | Gamma glutamyltransferase | Liver function |
| 30740 | -9.30E-05 | 0.0204543 | 79318 | Glucose | Endocrine |
| 30750 | -0.0001058 | 0.0035271 | 86095 | Glycated haemoglobin (HbA1c) | Immunometabolism |
| 30760 | 0.0005307 | 1.30E-39 | 79375 | HDL cholesterol | Immunometabolism |
| 30770 | -6.43E-07 | 0.9883955 | 86256 | IGF-1 | Endocrine |
| 30780 | -0.0001085 | 0.0091084 | 86574 | LDL direct | Immunometabolism |
| 30790 | 5.40E-05 | 0.2914645 | 69533 | Lipoprotein A | Endocrine |
| 30800 | 0.00024823 | 0.0229824 | 15127 | Oestradiol | Endocrine |
| 30810 | 7.85E-05 | 0.0908458 | 79236 | Phosphate | Renal function |
| 30820 | -0.0001125 | 0.4937593 | 7398 | Rheumatoid factor | Immunometabolism |
| 30830 | 0.00018686 | 1.18E-05 | 78611 | SHBG | Endocrine |
| 30840 | 0.00014503 | 0.0016694 | 86377 | Total bilirubin | Liver function |
| 30850 | 0.00010893 | 5.41E-08 | 78316 | Testosterone | Endocrine |
| 30860 | -5.37E-05 | 0.2728133 | 79336 | Total protein | Immunometabolism |
| 30870 | -0.000299 | 2.60E-13 | 86664 | Triglycerides | Immunometabolism |
| 30880 | -5.14E-05 | 0.1434752 | 86638 | Urate | Renal function |
| 30890 | 0.00062365 | 9.73E-44 | 83048 | Vitamin D | Bone and joint |

| **Table S7. Associations of Blood and Metabolomic Biomarkers with acrophase.** | | | | | |
| --- | --- | --- | --- | --- | --- |
| **Field ID** | **Beta** | **P Value** | **Sample size** | **Biomarker** | **Group** |
| 20280 | 0.00700204 | 0.0137838 | 49747 | Glucose-lactate | Glycolysis related metabolites |
| 20281 | 9.98E-03 | 0.0010283 | 49747 | Spectrometer-corrected alanine | Amino acids |
| 23400 | -0.0081297 | 0.0026971 | 49747 | Total_C | Cholesterol |
| 23401 | -3.83E-03 | 0.1701402 | 49747 | Non_HDL_C | Cholesterol |
| 23402 | -3.33E-03 | 0.233805 | 49747 | Remnant_C | Cholesterol |
| 23403 | 0.00213344 | 4.58E-01 | 49747 | VLDL Cholesterol | Cholesterol |
| 23404 | -4.87E-03 | 0.0802448 | 49747 | Clinical_LDL_C | Cholesterol |
| 23405 | -4.39E-03 | 0.1215904 | 49747 | LDL_C | Cholesterol |
| 23406 | -0.0142253 | 3.51E-08 | 49747 | HDL_C | Cholesterol |
| 23407 | 0.00752454 | 7.82E-03 | 49747 | Total_TG | Triglycerides |
| 23408 | 0.00833587 | 2.95E-03 | 49747 | VLDL_TG | Triglycerides |
| 23409 | 0.00463247 | 1.08E-01 | 49747 | LDL_TG | Triglycerides |
| 23410 | 0.00033305 | 0.9096277 | 49747 | HDL_TG | Triglycerides |
| 23411 | -0.0096445 | 0.0005023 | 49747 | Total_PL | Phospholipids |
| 23412 | 0.00504504 | 7.83E-02 | 49747 | VLDL_PL | Phospholipids |
| 23413 | -3.84E-03 | 0.1788401 | 49747 | LDL_PL | Phospholipids |
| 23414 | -0.0143318 | 6.23E-08 | 49747 | HDL_PL | Phospholipids |
| 23415 | -0.0089823 | 0.0008875 | 49747 | Total_CE | Cholesteryl Esters |
| 23416 | 0.00065187 | 0.8197634 | 49747 | VLDL_CE | Cholesteryl Esters |
| 23417 | -3.82E-03 | 0.1825398 | 49747 | LDL_CE | Cholesteryl Esters |
| 23418 | -0.0145719 | 1.86E-08 | 49747 | HDL_CE | Cholesteryl Esters |
| 23419 | -5.98E-03 | 0.0297327 | 49747 | Total_FC | Free Cholesterol |
| 23420 | 0.00305492 | 2.89E-01 | 49747 | VLDL_FC | Free Cholesterol |
| 23421 | -6.59E-03 | 0.0185908 | 49747 | LDL_FC | Free Cholesterol |
| 23422 | -0.0130416 | 6.45E-07 | 49747 | HDL_FC | Free Cholesterol |
| 23423 | -4.87E-03 | 0.0859698 | 49747 | Total_L | Total Lipids |
| 23424 | 0.00628643 | 2.74E-02 | 49747 | VLDL_L | Total Lipids |
| 23425 | -3.60E-03 | 0.2107499 | 49747 | LDL_L | Total Lipids |
| 23426 | -0.0141407 | 6.50E-08 | 49747 | HDL_L | Total Lipids |
| 23427 | -0.0152569 | 4.23E-08 | 49747 | Total_P | Particle Concentrations |
| 23428 | 0.00388409 | 1.80E-01 | 49747 | VLDL_P | Particle Concentrations |
| 23429 | -0.0004175 | 0.8835973 | 49747 | LDL_P | Particle Concentrations |
| 23430 | -0.0158395 | 1.04E-08 | 49747 | HDL_P | Particle Concentrations |
| 23431 | 0.00884491 | 1.11E-03 | 49747 | VLDL_size | Lipoprotein particle size |
| 23432 | -0.0071974 | 0.0126196 | 49747 | LDL_size | Lipoprotein particle size |
| 23433 | -0.007573 | 3.77E-03 | 49747 | HDL_size | Lipoprotein particle size |
| 23434 | -0.0104798 | 1.80E-04 | 49747 | Phosphoglycerides | Other lipids |
| 23436 | -0.0100556 | 3.41E-04 | 49747 | Cholines | Other lipids |
| 23437 | -0.0094525 | 6.08E-04 | 49747 | Phosphatidylc | Other lipids |
| 23438 | -0.0121019 | 8.38E-06 | 49747 | Sphingomyelins | Other lipids |
| 23439 | -0.0008684 | 0.7589828 | 49747 | ApoB | Apolipoproteins |
| 23440 | -0.0151766 | 1.50E-08 | 49747 | ApoA1 | Apolipoproteins |
| 23442 | -0.0022922 | 0.437662 | 49747 | Total_FA | Fatty acids |
| 23443 | -0.0128765 | 5.23E-06 | 49747 | Unsaturation | Fatty acids |
| 23444 | -8.99E-03 | 0.00231 | 49747 | Omega_3 | Fatty acids |
| 23445 | -4.71E-03 | 0.1069901 | 49747 | Omega_6 | Fatty acids |
| 23446 | -6.47E-03 | 0.027213 | 49747 | PUFA | Fatty acids |
| 23447 | 0.00237289 | 4.09E-01 | 49747 | MUFA | Fatty acids |
| 23448 | -0.0026802 | 0.3533054 | 49747 | SFA | Fatty acids |
| 23449 | -4.03E-04 | 0.8892226 | 49747 | LA | Fatty acids |
| 23450 | -1.05E-02 | 0.0002993 | 49747 | DHA | Fatty acids |
| 23460 | 5.48E-03 | 0.0746262 | 49747 | Alanine | Amino acids |
| 23461 | 0.00149386 | 0.6225653 | 49747 | Glutamine | Amino acids |
| 23462 | 2.79E-03 | 0.3412301 | 49747 | Glycine | Amino acids |
| 23463 | -2.57E-03 | 0.4036242 | 49747 | Histidine | Amino acids |
| 23464 | 4.53E-03 | 0.1244603 | 49747 | Total_BCAA | Amino acids |
| 23465 | 0.00247945 | 0.4216585 | 49747 | Isoleucine | Amino acids |
| 23466 | 2.30E-03 | 0.4415528 | 49747 | Leucine | Amino acids |
| 23467 | 0.00659795 | 0.0247054 | 49747 | Valine | Amino acids |
| 23468 | 6.33E-03 | 0.0257909 | 49747 | Phenylalanine | Amino acids |
| 23469 | 4.91E-03 | 0.1080251 | 49747 | Tyrosine | Amino acids |
| 23470 | 1.04E-02 | 0.0002968 | 49747 | Glucose | Glycolysis related metabolites |
| 23471 | -6.77E-03 | 0.0282125 | 49747 | Lactate | Glycolysis related metabolites |
| 23472 | -2.44E-04 | 0.9389194 | 49747 | Pyruvate | Glycolysis related metabolites |
| 23473 | -0.007833 | 1.02E-02 | 49747 | Citrate | Glycolysis related metabolites |
| 23474 | -1.25E-02 | 0.0000321 | 49747 | bOHbutyrate | Ketone bodies |
| 23475 | 6.87E-04 | 0.8214363 | 49747 | Acetate | Ketone bodies |
| 23476 | -1.12E-02 | 0.0001609 | 49747 | Acetoacetate | Ketone bodies |
| 23477 | -1.55E-02 | 2.16E-07 | 49747 | Acetone | Ketone bodies |
| 23478 | -9.09E-04 | 0.7739488 | 49747 | Creatinine | Fluid balance |
| 23479 | -0.0119672 | 0.0000695 | 49747 | Albumin | Fluid balance |
| 23480 | 0.00498208 | 7.95E-02 | 49747 | GlycA | Inflammation |
| 23481 | 0.00834861 | 2.50E-03 | 49747 | XXL_VLDL_P | Chylomicrons and Extremely Large VLDL |
| 23482 | 0.00805276 | 3.51E-03 | 49747 | XXL_VLDL_L | Chylomicrons and Extremely Large VLDL |
| 23483 | 0.00773176 | 5.01E-03 | 49747 | XXL_VLDL_PL | Chylomicrons and Extremely Large VLDL |
| 23484 | 0.00669195 | 1.62E-02 | 49747 | XXL_VLDL_C | Chylomicrons and Extremely Large VLDL |
| 23485 | 0.00564092 | 4.37E-02 | 49747 | XXL_VLDL_CE | Chylomicrons and Extremely Large VLDL |
| 23486 | 0.00703951 | 1.13E-02 | 49747 | XXL_VLDL_FC | Chylomicrons and Extremely Large VLDL |
| 23487 | 8.49E-04 | 0.7915135 | 49747 | XXL_VLDL_TG | Chylomicrons and Extremely Large VLDL |
| 23488 | 0.00801013 | 3.85E-03 | 49747 | XL_VLDL_P | Very Large VLDL |
| 23489 | 0.00851119 | 2.16E-03 | 49747 | XL_VLDL_L | Very Large VLDL |
| 23490 | -0.0030739 | 0.3672879 | 49747 | XL_VLDL_PL | Very Large VLDL |
| 23491 | 0.00660592 | 1.91E-02 | 49747 | XL_VLDL_C | Very Large VLDL |
| 23492 | 0.00427354 | 1.62E-01 | 49747 | XL_VLDL_CE | Very Large VLDL |
| 23493 | 0.00563406 | 6.91E-03 | 49747 | XL_VLDL_FC | Very Large VLDL |
| 23494 | 0.00952652 | 6.33E-04 | 49747 | XL_VLDL_TG | Very Large VLDL |
| 23495 | 0.00787154 | 4.96E-03 | 49747 | L_VLDL_P | Large VLDL |
| 23496 | 0.00793297 | 4.80E-03 | 49747 | L_VLDL_L | Large VLDL |
| 23497 | 0.00715807 | 0.0349735 | 49747 | L_VLDL_PL | Large VLDL |
| 23498 | 0.00630177 | 2.74E-02 | 49747 | L_VLDL_C | Large VLDL |
| 23499 | 0.00503819 | 8.27E-02 | 49747 | L_VLDL_CE | Large VLDL |
| 23500 | 0.0073761 | 8.64E-03 | 49747 | L_VLDL_FC | Large VLDL |
| 23501 | 0.00807112 | 4.13E-03 | 49747 | L_VLDL_TG | Large VLDL |
| 23502 | 0.0024272 | 4.01E-01 | 49747 | M_VLDL_P | Medium VLDL |
| 23503 | 0.00401267 | 1.65E-01 | 49747 | M_VLDL_L | Medium VLDL |
| 23504 | 0.0020558 | 4.77E-01 | 49747 | M_VLDL_PL | Medium VLDL |
| 23505 | -0.0012366 | 0.6629597 | 49747 | M_VLDL_C | Medium VLDL |
| 23506 | -3.19E-04 | 0.9099036 | 49747 | M_VLDL_CE | Medium VLDL |
| 23507 | 0.00096212 | 0.7387521 | 49747 | M_VLDL_FC | Medium VLDL |
| 23508 | 0.00669919 | 1.90E-02 | 49747 | M_VLDL_TG | Medium VLDL |
| 23509 | 0.00525849 | 7.09E-02 | 49747 | S_VLDL_P | Small VLDL |
| 23510 | 0.0046495 | 1.11E-01 | 49747 | S_VLDL_L | Small VLDL |
| 23511 | 0.00242879 | 4.05E-01 | 49747 | S_VLDL_PL | Small VLDL |
| 23512 | 0.00161396 | 5.79E-01 | 49747 | S_VLDL_C | Small VLDL |
| 23513 | 0.00244881 | 4.03E-01 | 49747 | S_VLDL_CE | Small VLDL |
| 23514 | 0.00047089 | 0.8707436 | 49747 | S_VLDL_FC | Small VLDL |
| 23515 | 0.00730481 | 1.11E-02 | 49747 | S_VLDL_TG | Small VLDL |
| 23516 | -0.0001508 | 0.9580371 | 49747 | XS_VLDL_P | Very Small VLDL |
| 23517 | -0.0008053 | 0.778758 | 49747 | XS_VLDL_L | Very Small VLDL |
| 23518 | 0.00125811 | 0.6652458 | 49747 | XS_VLDL_PL | Very Small VLDL |
| 23519 | -4.32E-03 | 0.1174296 | 49747 | XS_VLDL_C | Very Small VLDL |
| 23520 | -5.46E-03 | 0.0443418 | 49747 | XS_VLDL_CE | Very Small VLDL |
| 23521 | -0.0007592 | 0.7900424 | 49747 | XS_VLDL_FC | Very Small VLDL |
| 23522 | 0.00589139 | 4.22E-02 | 49747 | XS_VLDL_TG | Very Small VLDL |
| 23523 | -4.75E-03 | 0.0868325 | 49747 | IDL_P | IDL |
| 23524 | -7.66E-03 | 0.0042278 | 49747 | IDL_L | IDL |
| 23525 | -6.68E-03 | 0.013545 | 49747 | IDL_PL | IDL |
| 23526 | -0.0088967 | 0.0008134 | 49747 | IDL_C | IDL |
| 23527 | -0.0088584 | 0.0008895 | 49747 | IDL_CE | IDL |
| 23528 | -0.0086306 | 0.0013385 | 49747 | IDL_FC | IDL |
| 23529 | 0.00381704 | 0.1896183 | 49747 | IDL_TG | IDL |
| 23530 | -0.0011734 | 0.6803808 | 49747 | L_LDL_P | Large LDL |
| 23531 | -5.57E-03 | 0.048788 | 49747 | L_LDL_L | Large LDL |
| 23532 | -6.45E-03 | 0.0227824 | 49747 | L_LDL_PL | Large LDL |
| 23533 | -6.22E-03 | 0.027261 | 49747 | L_LDL_C | Large LDL |
| 23534 | -5.41E-03 | 0.0574953 | 49747 | L_LDL_CE | Large LDL |
| 23535 | -8.36E-03 | 0.0025637 | 49747 | L_LDL_FC | Large LDL |
| 23536 | 0.00370275 | 0.201345 | 49747 | L_LDL_TG | Large LDL |
| 23537 | -0.0000171 | 0.995257 | 49747 | M_LDL_P | Medium LDL |
| 23538 | -0.0015227 | 0.5992169 | 49747 | M_LDL_L | Medium LDL |
| 23539 | -0.0018281 | 0.5303288 | 49747 | M_LDL_PL | Medium LDL |
| 23540 | -0.0021015 | 0.4676096 | 49747 | M_LDL_C | Medium LDL |
| 23541 | -0.0010591 | 0.714764 | 49747 | M_LDL_CE | Medium LDL |
| 23542 | -4.63E-03 | 0.1068066 | 49747 | M_LDL_FC | Medium LDL |
| 23543 | 0.00531528 | 6.28E-02 | 49747 | M_LDL_TG | Medium LDL |
| 23544 | 0.00241092 | 0.3984732 | 49747 | S_LDL_P | Small LDL |
| 23545 | 0.00110507 | 0.701086 | 49747 | S_LDL_L | Small LDL |
| 23546 | 0.00155902 | 0.5846534 | 49747 | S_LDL_PL | Small LDL |
| 23547 | -0.0008162 | 0.7754109 | 49747 | S_LDL_C | Small LDL |
| 23548 | -0.000251 | 0.9305504 | 49747 | S_LDL_CE | Small LDL |
| 23549 | -2.03E-03 | 0.4401751 | 49747 | S_LDL_FC | Small LDL |
| 23550 | 0.00709347 | 1.17E-02 | 49747 | S_LDL_TG | Small LDL |
| 23551 | -0.0056582 | 3.73E-02 | 49747 | XL_HDL_P | Very Large HDL |
| 23552 | -0.0058071 | 3.01E-02 | 49747 | XL_HDL_L | Very Large HDL |
| 23553 | -0.007253 | 8.67E-03 | 49747 | XL_HDL_PL | Very Large HDL |
| 23554 | -0.0058164 | 2.82E-02 | 49747 | XL_HDL_C | Very Large HDL |
| 23555 | -0.0032411 | 2.57E-01 | 49747 | XL_HDL_CE | Very Large HDL |
| 23556 | 0.00093189 | 0.7758104 | 49747 | XL_HDL_FC | Very Large HDL |
| 23557 | 1.32E-03 | 0.6847667 | 49747 | XL_HDL_TG | Very Large HDL |
| 23558 | -0.0102059 | 1.03E-04 | 49747 | L_HDL_P | Large HDL |
| 23559 | -0.0107889 | 2.47E-05 | 49747 | L_HDL_L | Large HDL |
| 23560 | -0.0111374 | 1.80E-05 | 49747 | L_HDL_PL | Large HDL |
| 23561 | -0.0100717 | 8.63E-05 | 49747 | L_HDL_C | Large HDL |
| 23562 | -0.0099422 | 1.18E-04 | 49747 | L_HDL_CE | Large HDL |
| 23563 | -0.0090199 | 4.17E-04 | 49747 | L_HDL_FC | Large HDL |
| 23564 | -4.31E-03 | 0.1678633 | 49747 | L_HDL_TG | Large HDL |
| 23565 | -3.00E-03 | 0.4254563 | 49747 | M_HDL_P | Medium HDL |
| 23566 | -0.0153235 | 1.42E-08 | 49747 | M_HDL_L | Medium HDL |
| 23567 | -0.0150444 | 3.73E-08 | 49747 | M_HDL_PL | Medium HDL |
| 23568 | -0.0160934 | 1.87E-09 | 49747 | M_HDL_C | Medium HDL |
| 23569 | -0.0159446 | 2.81E-09 | 49747 | M_HDL_CE | Medium HDL |
| 23570 | -0.0152415 | 1.39E-08 | 49747 | M_HDL_FC | Medium HDL |
| 23571 | -0.0011975 | 0.6840311 | 49747 | M_HDL_TG | Medium HDL |
| 23572 | -0.0120553 | 0.0000543 | 49747 | S_HDL_P | Small HDL |
| 23573 | -0.011599 | 0.0000806 | 49747 | S_HDL_L | Small HDL |
| 23574 | -0.0125459 | 0.0000164 | 49747 | S_HDL_PL | Small HDL |
| 23575 | -0.0127752 | 0.0000184 | 49747 | S_HDL_C | Small HDL |
| 23576 | -0.0128621 | 0.0000199 | 49747 | S_HDL_CE | Small HDL |
| 23577 | -0.0110771 | 0.0001508 | 49747 | S_HDL_FC | Small HDL |
| 23578 | 0.00491583 | 8.02E-02 | 49747 | S_HDL_TG | Small HDL |
| 30000 | -0.0081608 | 2.08E-04 | 88326 | White blood cell (leukocyte) count | White blood cell |
| 30010 | -0.0003891 | 8.39E-01 | 88328 | Red blood cell (erythrocyte) count | Red blood cell |
| 30020 | -3.97E-03 | 0.0266069 | 88327 | Haemoglobin concentration | Red blood cell |
| 30030 | -3.66E-03 | 0.0475941 | 88328 | Haematocrit percentage | Red blood cell |
| 30040 | -0.0055786 | 9.32E-03 | 88328 | Mean corpuscular volume | Red blood cell |
| 30050 | -0.0053347 | 1.22E-02 | 88326 | Mean corpuscular haemoglobin | Red blood cell |
| 30060 | -1.46E-03 | 0.5279845 | 88326 | Mean corpuscular haemoglobin concentration | Red blood cell |
| 30070 | 4.44E-03 | 0.045387 | 88328 | Red blood cell (erythrocyte) distribution width | Red blood cell |
| 30080 | -3.49E-03 | 0.1121951 | 88328 | Platelet count | Platelet |
| 30090 | -3.74E-03 | 0.0850804 | 88327 | Platelet crit | Platelet |
| 30100 | 2.71E-03 | 0.244669 | 88327 | Mean platelet (thrombocyte) volume | Platelet |
| 30110 | 4.44E-03 | 0.0539804 | 88327 | Platelet distribution width | Platelet |
| 30120 | -0.0044097 | 0.0469669 | 88148 | Lymphocyte count | White blood cell |
| 30130 | 0.00476792 | 2.77E-02 | 88148 | Monocyte count | White blood cell |
| 30140 | -0.0094476 | 2.31E-05 | 88148 | Neutrophill count | White blood cell |
| 30150 | 0.00827044 | 0.0002825 | 88148 | Eosinophill count | White blood cell |
| 30160 | -3.48E-03 | 0.1171516 | 88148 | Basophill count | White blood cell |
| 30170 | -1.71E-03 | 0.4322613 | 88147 | Nucleated red blood cell count | Red blood cell |
| 30180 | 0.00160377 | 0.4794127 | 88150 | Lymphocyte percentage | White blood cell |
| 30190 | 1.16E-02 | 1.87E-07 | 88150 | Monocyte percentage | White blood cell |
| 30200 | -4.61E-03 | 0.0686094 | 88150 | Neutrophill percentage | White blood cell |
| 30210 | 1.37E-02 | 4.09E-09 | 88150 | Eosinophill percentage | White blood cell |
| 30220 | -3.18E-03 | 0.1613315 | 88150 | Basophill percentage | White blood cell |
| 30230 | -1.46E-03 | 0.4867354 | 88146 | Nucleated red blood cell percentage | Red blood cell |
| 30240 | -1.29E-03 | 0.5162649 | 86572 | Reticulocyte percentage | Red blood cell |
| 30250 | -0.0014604 | 0.4849127 | 86572 | Reticulocyte count | Red blood cell |
| 30260 | 0.00076408 | 0.7393147 | 86572 | Mean reticulocyte volume | Red blood cell |
| 30270 | -0.0010411 | 6.42E-01 | 86572 | Mean sphered cell volume | Red blood cell |
| 30280 | 0.00933147 | 2.75E-05 | 86572 | Immature reticulocyte fraction | Red blood cell |
| 30290 | 0.00282517 | 4.56E-02 | 86572 | High light scatter reticulocyte percentage | Red blood cell |
| 30300 | 0.00380683 | 6.48E-02 | 86572 | High light scatter reticulocyte count | Red blood cell |
| 30600 | -1.53E-02 | 2.43E-10 | 79398 | Albumin | Liver function |
| 30610 | 0.00584456 | 0.0051444 | 86722 | Alkaline phosphatase | Bone and joint |
| 30620 | 0.00443116 | 0.0323703 | 86702 | Alanine aminotransferase | Liver function |
| 30630 | -0.013944 | 5.45E-11 | 78914 | Apolipoprotein A | Immunometabolism |
| 30640 | -0.0000978 | 9.64E-01 | 86362 | Apolipoprotein B | Immunometabolism |
| 30650 | -0.005636 | 8.66E-03 | 86422 | Aspartate aminotransferase | Liver function |
| 30660 | -0.0028602 | 0.2183648 | 74354 | Direct bilirubin | Liver function |
| 30670 | -0.0100533 | 2.07E-06 | 86666 | Urea | Renal function |
| 30680 | -8.39E-03 | 0.0005426 | 79386 | Calcium | Bone and joint |
| 30690 | -4.11E-03 | 0.0484571 | 86722 | Cholesterol | Immunometabolism |
| 30700 | 1.15E-04 | 0.9430996 | 86682 | Creatinine | Renal function |
| 30710 | 0.00582727 | 0.0051617 | 86541 | C-reactive protein | Immunometabolism |
| 30720 | 0.00833449 | 1.15E-06 | 86717 | Cystatin C | Renal function |
| 30730 | 4.36E-03 | 0.023534 | 86691 | Gamma glutamyltransferase | Liver function |
| 30740 | 7.55E-03 | 0.0002265 | 79318 | Glucose | Endocrine |
| 30750 | 0.01236004 | 1.15E-11 | 86095 | Glycated haemoglobin (HbA1c) | Immunometabolism |
| 30760 | -0.013099 | 1.87E-10 | 79375 | HDL cholesterol | Immunometabolism |
| 30770 | -7.74E-03 | 0.0005571 | 86256 | IGF-1 | Endocrine |
| 30780 | -0.001488 | 0.4799373 | 86574 | LDL direct | Immunometabolism |
| 30790 | -2.78E-03 | 0.2955529 | 69533 | Lipoprotein A | Endocrine |
| 30800 | -0.006336 | 0.2730369 | 15127 | Oestradiol | Endocrine |
| 30810 | -8.66E-04 | 0.7144659 | 79236 | Phosphate | Renal function |
| 30820 | -0.0066464 | 0.4357383 | 7398 | Rheumatoid factor | Immunometabolism |
| 30830 | -0.013365 | 7.30E-10 | 78611 | SHBG | Endocrine |
| 30840 | -0.0043377 | 0.0627758 | 86377 | Total bilirubin | Liver function |
| 30850 | 0.00041211 | 6.88E-01 | 78316 | Testosterone | Endocrine |
| 30860 | -6.95E-03 | 0.005424 | 79336 | Total protein | Immunometabolism |
| 30870 | 0.00760408 | 2.42E-04 | 86664 | Triglycerides | Immunometabolism |
| 30880 | 2.75E-03 | 0.1218445 | 86638 | Urate | Renal function |
| 30890 | -0.0312149 | 8.66E-42 | 83048 | Vitamin D | Bone and joint |

| **Table S8. Associations of Blood and Metabolomic Biomarkers with pseudo-F.** | | | | | |
| --- | --- | --- | --- | --- | --- |
| **Field ID** | **Beta** | **P Value** | **Sample size** | **Biomarker** | **Group** |
| 20280 | -0.0000261 | 0.1990302 | 49747 | Glucose-lactate | Glycolysis related metabolites |
| 20281 | 4.25E-05 | 0.0507959 | 49747 | Spectrometer-corrected alanine | Amino acids |
| 23400 | -6.67E-06 | 0.7308353 | 49747 | Total_C | Cholesterol |
| 23401 | -3.57E-05 | 0.0744148 | 49747 | Non_HDL_C | Cholesterol |
| 23402 | -3.51E-05 | 0.0794627 | 49747 | Remnant_C | Cholesterol |
| 23403 | -0.0000529 | 1.00E-02 | 49747 | VLDL Cholesterol | Cholesterol |
| 23404 | -3.26E-05 | 0.1014192 | 49747 | Clinical_LDL_C | Cholesterol |
| 23405 | -3.26E-05 | 0.1090274 | 49747 | LDL_C | Cholesterol |
| 23406 | 0.0000697 | 1.62E-04 | 49747 | HDL_C | Cholesterol |
| 23407 | -0.000042 | 3.81E-02 | 49747 | Total_TG | Triglycerides |
| 23408 | -0.0000445 | 2.65E-02 | 49747 | VLDL_TG | Triglycerides |
| 23409 | -0.0000311 | 1.31E-01 | 49747 | LDL_TG | Triglycerides |
| 23410 | -3.49E-06 | 0.8678174 | 49747 | HDL_TG | Triglycerides |
| 23411 | 0.0000119 | 0.5479528 | 49747 | Total_PL | Phospholipids |
| 23412 | -0.0000527 | 1.03E-02 | 49747 | VLDL_PL | Phospholipids |
| 23413 | -3.74E-05 | 0.0673004 | 49747 | LDL_PL | Phospholipids |
| 23414 | 0.0000693 | 2.56E-04 | 49747 | HDL_PL | Phospholipids |
| 23415 | -2.27E-06 | 0.9066183 | 49747 | Total_CE | Cholesteryl Esters |
| 23416 | -0.0000498 | 0.0149184 | 49747 | VLDL_CE | Cholesteryl Esters |
| 23417 | -3.73E-05 | 0.0689646 | 49747 | LDL_CE | Cholesteryl Esters |
| 23418 | 0.0000704 | 1.47E-04 | 49747 | HDL_CE | Cholesteryl Esters |
| 23419 | -1.90E-05 | 0.3337217 | 49747 | Total_FC | Free Cholesterol |
| 23420 | -0.0000535 | 9.53E-03 | 49747 | VLDL_FC | Free Cholesterol |
| 23421 | -2.32E-05 | 0.2479414 | 49747 | LDL_FC | Free Cholesterol |
| 23422 | 0.0000632 | 7.45E-04 | 49747 | HDL_FC | Free Cholesterol |
| 23423 | -1.50E-05 | 0.4613086 | 49747 | Total_L | Total Lipids |
| 23424 | -0.0000537 | 8.51E-03 | 49747 | VLDL_L | Total Lipids |
| 23425 | -3.93E-05 | 0.0563844 | 49747 | LDL_L | Total Lipids |
| 23426 | 0.0000696 | 2.02E-04 | 49747 | HDL_L | Total Lipids |
| 23427 | 0.0000443 | 2.60E-02 | 49747 | Total_P | Particle Concentrations |
| 23428 | -0.0000491 | 1.77E-02 | 49747 | VLDL_P | Particle Concentrations |
| 23429 | -0.0000469 | 0.0214113 | 49747 | LDL_P | Particle Concentrations |
| 23430 | 0.0000555 | 5.08E-03 | 49747 | HDL_P | Particle Concentrations |
| 23431 | -0.000052 | 7.39E-03 | 49747 | VLDL_size | Lipoprotein particle size |
| 23432 | 0.0000368 | 0.0749876 | 49747 | LDL_size | Lipoprotein particle size |
| 23433 | 0.000072 | 1.20E-04 | 49747 | HDL_size | Lipoprotein particle size |
| 23434 | 0.0000199 | 3.21E-01 | 49747 | Phosphoglycerides | Other lipids |
| 23436 | 0.000021 | 2.95E-01 | 49747 | Cholines | Other lipids |
| 23437 | 0.0000255 | 1.97E-01 | 49747 | Phosphatidylc | Other lipids |
| 23438 | 0.00000562 | 0.7723968 | 49747 | Sphingomyelins | Other lipids |
| 23439 | -0.0000478 | 0.0183887 | 49747 | ApoB | Apolipoproteins |
| 23440 | 0.0000658 | 6.06E-04 | 49747 | ApoA1 | Apolipoproteins |
| 23442 | -0.0000244 | 0.2484258 | 49747 | Total_FA | Fatty acids |
| 23443 | 0.000039 | 0.0541688 | 49747 | Unsaturation | Fatty acids |
| 23444 | 9.86E-06 | 0.6407966 | 49747 | Omega_3 | Fatty acids |
| 23445 | -5.82E-06 | 0.780678 | 49747 | Omega_6 | Fatty acids |
| 23446 | -1.89E-06 | 0.9281361 | 49747 | PUFA | Fatty acids |
| 23447 | -0.0000372 | 7.04E-02 | 49747 | MUFA | Fatty acids |
| 23448 | -0.0000244 | 0.2383417 | 49747 | SFA | Fatty acids |
| 23449 | -4.48E-06 | 0.8286259 | 49747 | LA | Fatty acids |
| 23450 | 2.52E-05 | 0.2244679 | 49747 | DHA | Fatty acids |
| 23460 | 4.30E-05 | 0.0510094 | 49747 | Alanine | Amino acids |
| 23461 | -0.0000343 | 0.114635 | 49747 | Glutamine | Amino acids |
| 23462 | -1.93E-05 | 0.3577457 | 49747 | Glycine | Amino acids |
| 23463 | -1.30E-05 | 0.5542863 | 49747 | Histidine | Amino acids |
| 23464 | -8.98E-06 | 0.6705361 | 49747 | Total_BCAA | Amino acids |
| 23465 | -0.0000149 | 0.4985187 | 49747 | Isoleucine | Amino acids |
| 23466 | -8.43E-06 | 0.6936872 | 49747 | Leucine | Amino acids |
| 23467 | -0.0000075 | 0.7213335 | 49747 | Valine | Amino acids |
| 23468 | 1.21E-05 | 0.5523622 | 49747 | Phenylalanine | Amino acids |
| 23469 | -3.73E-07 | 0.986366 | 49747 | Tyrosine | Amino acids |
| 23470 | -4.61E-05 | 0.0255701 | 49747 | Glucose | Glycolysis related metabolites |
| 23471 | 4.05E-05 | 0.0665749 | 49747 | Lactate | Glycolysis related metabolites |
| 23472 | 1.08E-04 | 2.25E-06 | 49747 | Pyruvate | Glycolysis related metabolites |
| 23473 | 0.0000447 | 4.07E-02 | 49747 | Citrate | Glycolysis related metabolites |
| 23474 | 1.92E-05 | 0.3734795 | 49747 | bOHbutyrate | Ketone bodies |
| 23475 | -1.75E-05 | 0.4208145 | 49747 | Acetate | Ketone bodies |
| 23476 | -2.14E-05 | 0.3112546 | 49747 | Acetoacetate | Ketone bodies |
| 23477 | 1.69E-05 | 0.428274 | 49747 | Acetone | Ketone bodies |
| 23478 | -2.55E-05 | 0.2607753 | 49747 | Creatinine | Fluid balance |
| 23479 | -6.71E-07 | 0.9751346 | 49747 | Albumin | Fluid balance |
| 23480 | -0.0000379 | 6.24E-02 | 49747 | GlycA | Inflammation |
| 23481 | -0.000039 | 4.82E-02 | 49747 | XXL_VLDL_P | Chylomicrons and Extremely Large VLDL |
| 23482 | -0.0000337 | 8.76E-02 | 49747 | XXL_VLDL_L | Chylomicrons and Extremely Large VLDL |
| 23483 | -0.0000317 | 1.08E-01 | 49747 | XXL_VLDL_PL | Chylomicrons and Extremely Large VLDL |
| 23484 | -0.0000328 | 9.98E-02 | 49747 | XXL_VLDL_C | Chylomicrons and Extremely Large VLDL |
| 23485 | -0.0000321 | 1.08E-01 | 49747 | XXL_VLDL_CE | Chylomicrons and Extremely Large VLDL |
| 23486 | -0.0000306 | 1.24E-01 | 49747 | XXL_VLDL_FC | Chylomicrons and Extremely Large VLDL |
| 23487 | -4.22E-06 | 0.8543182 | 49747 | XXL_VLDL_TG | Chylomicrons and Extremely Large VLDL |
| 23488 | -0.0000446 | 2.46E-02 | 49747 | XL_VLDL_P | Very Large VLDL |
| 23489 | -0.000048 | 1.57E-02 | 49747 | XL_VLDL_L | Very Large VLDL |
| 23490 | 0.0000293 | 0.2293273 | 49747 | XL_VLDL_PL | Very Large VLDL |
| 23491 | -0.0000541 | 7.32E-03 | 49747 | XL_VLDL_C | Very Large VLDL |
| 23492 | -0.0000579 | 8.16E-03 | 49747 | XL_VLDL_CE | Very Large VLDL |
| 23493 | -0.0000353 | 1.79E-02 | 49747 | XL_VLDL_FC | Very Large VLDL |
| 23494 | -0.0000407 | 4.13E-02 | 49747 | XL_VLDL_TG | Very Large VLDL |
| 23495 | -0.0000471 | 1.87E-02 | 49747 | L_VLDL_P | Large VLDL |
| 23496 | -0.0000517 | 1.02E-02 | 49747 | L_VLDL_L | Large VLDL |
| 23497 | -0.0000316 | 0.1929394 | 49747 | L_VLDL_PL | Large VLDL |
| 23498 | -0.0000561 | 6.11E-03 | 49747 | L_VLDL_C | Large VLDL |
| 23499 | -0.000056 | 7.01E-03 | 49747 | L_VLDL_CE | Large VLDL |
| 23500 | -0.0000536 | 7.61E-03 | 49747 | L_VLDL_FC | Large VLDL |
| 23501 | -0.0000461 | 2.21E-02 | 49747 | L_VLDL_TG | Large VLDL |
| 23502 | -0.0000501 | 1.54E-02 | 49747 | M_VLDL_P | Medium VLDL |
| 23503 | -0.0000539 | 9.19E-03 | 49747 | M_VLDL_L | Medium VLDL |
| 23504 | -0.000052 | 1.21E-02 | 49747 | M_VLDL_PL | Medium VLDL |
| 23505 | -0.0000454 | 0.02531 | 49747 | M_VLDL_C | Medium VLDL |
| 23506 | -3.47E-05 | 0.0849255 | 49747 | M_VLDL_CE | Medium VLDL |
| 23507 | -0.0000481 | 0.0198184 | 49747 | M_VLDL_FC | Medium VLDL |
| 23508 | -0.0000467 | 2.22E-02 | 49747 | M_VLDL_TG | Medium VLDL |
| 23509 | -0.0000495 | 1.76E-02 | 49747 | S_VLDL_P | Small VLDL |
| 23510 | -0.0000505 | 1.56E-02 | 49747 | S_VLDL_L | Small VLDL |
| 23511 | -0.0000516 | 1.35E-02 | 49747 | S_VLDL_PL | Small VLDL |
| 23512 | -0.0000538 | 9.74E-03 | 49747 | S_VLDL_C | Small VLDL |
| 23513 | -0.0000555 | 8.15E-03 | 49747 | S_VLDL_CE | Small VLDL |
| 23514 | -0.0000487 | 0.0187006 | 49747 | S_VLDL_FC | Small VLDL |
| 23515 | -0.0000358 | 8.22E-02 | 49747 | S_VLDL_TG | Small VLDL |
| 23516 | -0.0000358 | 0.0806606 | 49747 | XS_VLDL_P | Very Small VLDL |
| 23517 | -0.0000326 | 0.1124043 | 49747 | XS_VLDL_L | Very Small VLDL |
| 23518 | -0.0000369 | 0.0764953 | 49747 | XS_VLDL_PL | Very Small VLDL |
| 23519 | -2.56E-05 | 0.1940683 | 49747 | XS_VLDL_C | Very Small VLDL |
| 23520 | -2.02E-05 | 0.2976211 | 49747 | XS_VLDL_CE | Very Small VLDL |
| 23521 | -0.0000329 | 0.1066604 | 49747 | XS_VLDL_FC | Very Small VLDL |
| 23522 | -0.0000297 | 1.52E-01 | 49747 | XS_VLDL_TG | Very Small VLDL |
| 23523 | -3.44E-05 | 0.0829228 | 49747 | IDL_P | IDL |
| 23524 | -1.44E-05 | 0.4526497 | 49747 | IDL_L | IDL |
| 23525 | -1.89E-05 | 0.3290752 | 49747 | IDL_PL | IDL |
| 23526 | -0.0000107 | 0.5751522 | 49747 | IDL_C | IDL |
| 23527 | -9.68E-06 | 0.611805 | 49747 | IDL_CE | IDL |
| 23528 | -0.0000115 | 0.5487065 | 49747 | IDL_FC | IDL |
| 23529 | -0.0000269 | 0.1966682 | 49747 | IDL_TG | IDL |
| 23530 | -0.000041 | 0.044182 | 49747 | L_LDL_P | Large LDL |
| 23531 | -2.57E-05 | 0.2042975 | 49747 | L_LDL_L | Large LDL |
| 23532 | -2.41E-05 | 0.235581 | 49747 | L_LDL_PL | Large LDL |
| 23533 | -2.26E-05 | 0.2622275 | 49747 | L_LDL_C | Large LDL |
| 23534 | -2.52E-05 | 0.2165823 | 49747 | L_LDL_CE | Large LDL |
| 23535 | -1.50E-05 | 0.450556 | 49747 | L_LDL_FC | Large LDL |
| 23536 | -0.0000285 | 0.1698872 | 49747 | L_LDL_TG | Large LDL |
| 23537 | -0.0000523 | 0.0112401 | 49747 | M_LDL_P | Medium LDL |
| 23538 | -0.0000478 | 0.021139 | 49747 | M_LDL_L | Medium LDL |
| 23539 | -0.0000448 | 0.0315162 | 49747 | M_LDL_PL | Medium LDL |
| 23540 | -0.0000459 | 0.0266886 | 49747 | M_LDL_C | Medium LDL |
| 23541 | -0.0000494 | 0.0171693 | 49747 | M_LDL_CE | Medium LDL |
| 23542 | -3.03E-05 | 0.1404167 | 49747 | M_LDL_FC | Medium LDL |
| 23543 | -0.0000321 | 1.16E-01 | 49747 | M_LDL_TG | Medium LDL |
| 23544 | -0.0000548 | 0.0073149 | 49747 | S_LDL_P | Small LDL |
| 23545 | -0.0000543 | 0.0084252 | 49747 | S_LDL_L | Small LDL |
| 23546 | -0.0000527 | 0.0097662 | 49747 | S_LDL_PL | Small LDL |
| 23547 | -0.0000489 | 0.0169149 | 49747 | S_LDL_C | Small LDL |
| 23548 | -0.0000476 | 0.0209605 | 49747 | S_LDL_CE | Small LDL |
| 23549 | -3.55E-05 | 0.0594844 | 49747 | S_LDL_FC | Small LDL |
| 23550 | -0.000035 | 8.21E-02 | 49747 | S_LDL_TG | Small LDL |
| 23551 | 0.0000423 | 2.97E-02 | 49747 | XL_HDL_P | Very Large HDL |
| 23552 | 0.000046 | 1.63E-02 | 49747 | XL_HDL_L | Very Large HDL |
| 23553 | 0.0000441 | 2.59E-02 | 49747 | XL_HDL_PL | Very Large HDL |
| 23554 | 0.0000398 | 3.58E-02 | 49747 | XL_HDL_C | Very Large HDL |
| 23555 | 0.0000334 | 1.02E-01 | 49747 | XL_HDL_CE | Very Large HDL |
| 23556 | 0.0000188 | 0.4231092 | 49747 | XL_HDL_FC | Very Large HDL |
| 23557 | -4.30E-06 | 0.8536643 | 49747 | XL_HDL_TG | Very Large HDL |
| 23558 | 0.0000643 | 6.35E-04 | 49747 | L_HDL_P | Large HDL |
| 23559 | 0.0000707 | 1.14E-04 | 49747 | L_HDL_L | Large HDL |
| 23560 | 0.0000723 | 1.01E-04 | 49747 | L_HDL_PL | Large HDL |
| 23561 | 0.0000646 | 4.30E-04 | 49747 | L_HDL_C | Large HDL |
| 23562 | 0.0000648 | 4.51E-04 | 49747 | L_HDL_CE | Large HDL |
| 23563 | 0.0000577 | 1.60E-03 | 49747 | L_HDL_FC | Large HDL |
| 23564 | 2.78E-05 | 0.2138877 | 49747 | L_HDL_TG | Large HDL |
| 23565 | 8.63E-06 | 0.7489385 | 49747 | M_HDL_P | Medium HDL |
| 23566 | 0.0000677 | 4.67E-04 | 49747 | M_HDL_L | Medium HDL |
| 23567 | 0.0000652 | 8.55E-04 | 49747 | M_HDL_PL | Medium HDL |
| 23568 | 0.0000718 | 1.78E-04 | 49747 | M_HDL_C | Medium HDL |
| 23569 | 0.0000719 | 1.80E-04 | 49747 | M_HDL_CE | Medium HDL |
| 23570 | 0.0000674 | 4.51E-04 | 49747 | M_HDL_FC | Medium HDL |
| 23571 | -5.27E-07 | 0.9800392 | 49747 | M_HDL_TG | Medium HDL |
| 23572 | 0.0000136 | 0.5233596 | 49747 | S_HDL_P | Small HDL |
| 23573 | 0.0000175 | 0.4064286 | 49747 | S_HDL_L | Small HDL |
| 23574 | 0.0000253 | 0.2253689 | 49747 | S_HDL_PL | Small HDL |
| 23575 | 0.0000186 | 0.3845692 | 49747 | S_HDL_C | Small HDL |
| 23576 | 0.0000179 | 0.4056705 | 49747 | S_HDL_CE | Small HDL |
| 23577 | 0.0000182 | 0.3855799 | 49747 | S_HDL_FC | Small HDL |
| 23578 | -0.0000327 | 1.04E-01 | 49747 | S_HDL_TG | Small HDL |
| 30000 | 0.00000851 | 5.94E-01 | 88326 | White blood cell (leukocyte) count | White blood cell |
| 30010 | -0.0000298 | 3.25E-02 | 88328 | Red blood cell (erythrocyte) count | Red blood cell |
| 30020 | -2.35E-05 | 0.0706709 | 88327 | Haemoglobin concentration | Red blood cell |
| 30030 | -1.72E-05 | 0.1987879 | 88328 | Haematocrit percentage | Red blood cell |
| 30040 | 0.0000245 | 1.16E-01 | 88328 | Mean corpuscular volume | Red blood cell |
| 30050 | 0.0000123 | 4.26E-01 | 88326 | Mean corpuscular haemoglobin | Red blood cell |
| 30060 | -1.81E-05 | 0.2800714 | 88326 | Mean corpuscular haemoglobin concentration | Red blood cell |
| 30070 | 3.41E-05 | 0.0344475 | 88328 | Red blood cell (erythrocyte) distribution width | Red blood cell |
| 30080 | -4.59E-05 | 0.0040865 | 88328 | Platelet count | Platelet |
| 30090 | -3.09E-05 | 0.0503229 | 88327 | Platelet crit | Platelet |
| 30100 | 4.92E-05 | 0.0036869 | 88327 | Mean platelet (thrombocyte) volume | Platelet |
| 30110 | 9.79E-06 | 0.5583992 | 88327 | Platelet distribution width | Platelet |
| 30120 | -0.0000137 | 0.395234 | 88148 | Lymphocyte count | White blood cell |
| 30130 | -5.78E-06 | 7.13E-01 | 88148 | Monocyte count | White blood cell |
| 30140 | 0.0000231 | 1.54E-01 | 88148 | Neutrophill count | White blood cell |
| 30150 | -0.0000181 | 0.2734318 | 88148 | Eosinophill count | White blood cell |
| 30160 | 2.38E-05 | 0.1395043 | 88148 | Basophill count | White blood cell |
| 30170 | 7.63E-06 | 0.6289023 | 88147 | Nucleated red blood cell count | Red blood cell |
| 30180 | -0.0000225 | 0.1716553 | 88150 | Lymphocyte percentage | White blood cell |
| 30190 | -2.17E-05 | 0.1781683 | 88150 | Monocyte percentage | White blood cell |
| 30200 | 2.26E-05 | 0.2195533 | 88150 | Neutrophill percentage | White blood cell |
| 30210 | -3.30E-05 | 0.0513109 | 88150 | Eosinophill percentage | White blood cell |
| 30220 | 1.94E-05 | 0.2381544 | 88150 | Basophill percentage | White blood cell |
| 30230 | 4.67E-06 | 0.7598035 | 88146 | Nucleated red blood cell percentage | Red blood cell |
| 30240 | -6.92E-06 | 0.6307766 | 86572 | Reticulocyte percentage | Red blood cell |
| 30250 | -0.0000131 | 0.3870792 | 86572 | Reticulocyte count | Red blood cell |
| 30260 | 0.0000197 | 0.2364559 | 86572 | Mean reticulocyte volume | Red blood cell |
| 30270 | 0.0000427 | 8.42E-03 | 86572 | Mean sphered cell volume | Red blood cell |
| 30280 | -0.0000354 | 2.79E-02 | 86572 | Immature reticulocyte fraction | Red blood cell |
| 30290 | -0.0000184 | 7.14E-02 | 86572 | High light scatter reticulocyte percentage | Red blood cell |
| 30300 | -0.0000329 | 2.75E-02 | 86572 | High light scatter reticulocyte count | Red blood cell |
| 30600 | 1.33E-05 | 0.4485051 | 79398 | Albumin | Liver function |
| 30610 | -0.0000408 | 0.0070078 | 86722 | Alkaline phosphatase | Bone and joint |
| 30620 | -0.0000203 | 0.1759948 | 86702 | Alanine aminotransferase | Liver function |
| 30630 | 0.0000759 | 8.28E-07 | 78914 | Apolipoprotein A | Immunometabolism |
| 30640 | -0.0000507 | 1.25E-03 | 86362 | Apolipoprotein B | Immunometabolism |
| 30650 | 0.0000461 | 3.06E-03 | 86422 | Aspartate aminotransferase | Liver function |
| 30660 | 0.0000403 | 0.0149493 | 74354 | Direct bilirubin | Liver function |
| 30670 | 0.0000502 | 1.09E-03 | 86666 | Urea | Renal function |
| 30680 | -3.40E-06 | 0.8463423 | 79386 | Calcium | Bone and joint |
| 30690 | -1.99E-05 | 0.1863246 | 86722 | Cholesterol | Immunometabolism |
| 30700 | -3.55E-06 | 0.7615645 | 86682 | Creatinine | Renal function |
| 30710 | -0.0000126 | 0.4032644 | 86541 | C-reactive protein | Immunometabolism |
| 30720 | -3.36E-06 | 7.87E-01 | 86717 | Cystatin C | Renal function |
| 30730 | 4.56E-05 | 0.0010769 | 86691 | Gamma glutamyltransferase | Liver function |
| 30740 | -1.53E-05 | 0.3013272 | 79318 | Glucose | Endocrine |
| 30750 | -4.35E-06 | 0.7416025 | 86095 | Glycated haemoglobin (HbA1c) | Immunometabolism |
| 30760 | 0.0000788 | 1.19E-07 | 79375 | HDL cholesterol | Immunometabolism |
| 30770 | 9.22E-06 | 0.569917 | 86256 | IGF-1 | Endocrine |
| 30780 | -0.0000411 | 0.0070973 | 86574 | LDL direct | Immunometabolism |
| 30790 | -1.80E-05 | 0.3351294 | 69533 | Lipoprotein A | Endocrine |
| 30800 | 0.00010017 | 0.0204668 | 15127 | Oestradiol | Endocrine |
| 30810 | 5.10E-06 | 0.7658297 | 79236 | Phosphate | Renal function |
| 30820 | -0.0000171 | 0.7883659 | 7398 | Rheumatoid factor | Immunometabolism |
| 30830 | 0.0000554 | 4.41E-04 | 78611 | SHBG | Endocrine |
| 30840 | 0.0000288 | 0.0879609 | 86377 | Total bilirubin | Liver function |
| 30850 | 0.0000255 | 5.49E-04 | 78316 | Testosterone | Endocrine |
| 30860 | 1.91E-05 | 0.2915603 | 79336 | Total protein | Immunometabolism |
| 30870 | -0.0000302 | 4.43E-02 | 86664 | Triglycerides | Immunometabolism |
| 30880 | 1.56E-05 | 0.2260143 | 86638 | Urate | Renal function |
| 30890 | 0.0000609 | 2.30E-04 | 83048 | Vitamin D | Bone and joint |

| **Table S9. Associations of Blood and Metabolomic Biomarkers with mesor.** | | | | | |
| --- | --- | --- | --- | --- | --- |
| **Field ID** | **Beta** | **P Value** | **Sample size** | **Biomarker** | **Group** |
| 20280 | -0.0002759 | 0.0091772 | 49747 | Glucose-lactate | Glycolysis related metabolites |
| 20281 | 1.53E-04 | 0.1761651 | 49747 | Spectrometer-corrected alanine | Amino acids |
| 23400 | 0.00026703 | 0.0081419 | 49747 | Total_C | Cholesterol |
| 23401 | -8.50E-05 | 0.4139563 | 49747 | Non_HDL_C | Cholesterol |
| 23402 | -1.59E-04 | 0.1279513 | 49747 | Remnant_C | Cholesterol |
| 23403 | -0.0004836 | 6.16E-06 | 49747 | VLDL Cholesterol | Cholesterol |
| 23404 | 6.86E-06 | 0.9472424 | 49747 | Clinical_LDL_C | Cholesterol |
| 23405 | -1.93E-05 | 0.855154 | 49747 | LDL_C | Cholesterol |
| 23406 | 0.00099402 | 4.19E-25 | 49747 | HDL_C | Cholesterol |
| 23407 | -0.0006122 | 6.19E-09 | 49747 | Total_TG | Triglycerides |
| 23408 | -0.0006449 | 6.53E-10 | 49747 | VLDL_TG | Triglycerides |
| 23409 | -0.0004308 | 5.84E-05 | 49747 | LDL_TG | Triglycerides |
| 23410 | -0.0002517 | 0.0212646 | 49747 | HDL_TG | Triglycerides |
| 23411 | 0.00035683 | 0.0005466 | 49747 | Total_PL | Phospholipids |
| 23412 | -0.0006021 | 1.68E-08 | 49747 | VLDL_PL | Phospholipids |
| 23413 | -9.52E-05 | 0.3708618 | 49747 | LDL_PL | Phospholipids |
| 23414 | 0.00088883 | 1.97E-19 | 49747 | HDL_PL | Phospholipids |
| 23415 | 0.00032351 | 0.0013062 | 49747 | Total_CE | Cholesteryl Esters |
| 23416 | -0.0004108 | 0.0001154 | 49747 | VLDL_CE | Cholesteryl Esters |
| 23417 | -7.81E-05 | 0.4642692 | 49747 | LDL_CE | Cholesteryl Esters |
| 23418 | 0.00100806 | 1.44E-25 | 49747 | HDL_CE | Cholesteryl Esters |
| 23419 | 9.95E-05 | 0.331695 | 49747 | Total_FC | Free Cholesterol |
| 23420 | -0.0005503 | 2.94E-07 | 49747 | VLDL_FC | Free Cholesterol |
| 23421 | 1.03E-04 | 0.3222138 | 49747 | LDL_FC | Free Cholesterol |
| 23422 | 0.00089151 | 6.32E-20 | 49747 | HDL_FC | Free Cholesterol |
| 23423 | 6.05E-05 | 0.5670876 | 49747 | Total_L | Total Lipids |
| 23424 | -0.0006366 | 1.99E-09 | 49747 | VLDL_L | Total Lipids |
| 23425 | -6.61E-05 | 0.537274 | 49747 | LDL_L | Total Lipids |
| 23426 | 0.00092941 | 1.41E-21 | 49747 | HDL_L | Total Lipids |
| 23427 | 0.00073436 | 1.40E-12 | 49747 | Total_P | Particle Concentrations |
| 23428 | -0.000508 | 2.44E-06 | 49747 | VLDL_P | Particle Concentrations |
| 23429 | -0.0002764 | 0.0092405 | 49747 | LDL_P | Particle Concentrations |
| 23430 | 0.00080073 | 7.82E-15 | 49747 | HDL_P | Particle Concentrations |
| 23431 | -0.0007167 | 1.26E-12 | 49747 | VLDL_size | Lipoprotein particle size |
| 23432 | 0.0003113 | 0.0037685 | 49747 | LDL_size | Lipoprotein particle size |
| 23433 | 0.00088285 | 1.19E-19 | 49747 | HDL_size | Lipoprotein particle size |
| 23434 | 0.00042905 | 3.83E-05 | 49747 | Phosphoglycerides | Other lipids |
| 23436 | 0.00054329 | 2.02E-07 | 49747 | Cholines | Other lipids |
| 23437 | 0.00051579 | 5.08E-07 | 49747 | Phosphatidylc | Other lipids |
| 23438 | 0.00034724 | 0.0005982 | 49747 | Sphingomyelins | Other lipids |
| 23439 | -0.0002718 | 0.0099147 | 49747 | ApoB | Apolipoproteins |
| 23440 | 0.00089409 | 3.28E-19 | 49747 | ApoA1 | Apolipoproteins |
| 23442 | -0.0001936 | 0.0782935 | 49747 | Total_FA | Fatty acids |
| 23443 | 0.00037823 | 0.000327 | 49747 | Unsaturation | Fatty acids |
| 23444 | -1.37E-04 | 0.2123574 | 49747 | Omega_3 | Fatty acids |
| 23445 | 1.25E-04 | 0.2515848 | 49747 | Omega_6 | Fatty acids |
| 23446 | 7.86E-05 | 0.4713099 | 49747 | PUFA | Fatty acids |
| 23447 | -0.000426 | 6.89E-05 | 49747 | MUFA | Fatty acids |
| 23448 | -0.0002102 | 0.0506641 | 49747 | SFA | Fatty acids |
| 23449 | 1.67E-04 | 0.1200487 | 49747 | LA | Fatty acids |
| 23450 | 1.24E-04 | 0.2504376 | 49747 | DHA | Fatty acids |
| 23460 | 3.46E-05 | 0.7625272 | 49747 | Alanine | Amino acids |
| 23461 | 0.00022187 | 0.0496426 | 49747 | Glutamine | Amino acids |
| 23462 | 5.42E-05 | 0.6189776 | 49747 | Glycine | Amino acids |
| 23463 | -1.85E-05 | 0.8716525 | 49747 | Histidine | Amino acids |
| 23464 | -1.92E-04 | 0.0800031 | 49747 | Total_BCAA | Amino acids |
| 23465 | -0.000275 | 0.0166923 | 49747 | Isoleucine | Amino acids |
| 23466 | -9.09E-05 | 0.4142322 | 49747 | Leucine | Amino acids |
| 23467 | -0.0002403 | 0.0280524 | 49747 | Valine | Amino acids |
| 23468 | 6.99E-05 | 0.5087047 | 49747 | Phenylalanine | Amino acids |
| 23469 | -1.25E-04 | 0.2707689 | 49747 | Tyrosine | Amino acids |
| 23470 | -1.93E-04 | 0.0720705 | 49747 | Glucose | Glycolysis related metabolites |
| 23471 | -1.81E-04 | 0.1162106 | 49747 | Lactate | Glycolysis related metabolites |
| 23472 | 1.31E-04 | 0.2679383 | 49747 | Pyruvate | Glycolysis related metabolites |
| 23473 | 0.00047086 | 3.38E-05 | 49747 | Citrate | Glycolysis related metabolites |
| 23474 | -3.42E-06 | 0.9757539 | 49747 | bOHbutyrate | Ketone bodies |
| 23475 | -9.69E-05 | 0.3924999 | 49747 | Acetate | Ketone bodies |
| 23476 | -1.21E-04 | 0.2724627 | 49747 | Acetoacetate | Ketone bodies |
| 23477 | 1.08E-04 | 0.3307606 | 49747 | Acetone | Ketone bodies |
| 23478 | -1.07E-04 | 0.3654535 | 49747 | Creatinine | Fluid balance |
| 23479 | 0.00025741 | 0.0215966 | 49747 | Albumin | Fluid balance |
| 23480 | -0.000808 | 2.18E-14 | 49747 | GlycA | Inflammation |
| 23481 | -0.0006073 | 3.50E-09 | 49747 | XXL_VLDL_P | Chylomicrons and Extremely Large VLDL |
| 23482 | -0.0005947 | 7.08E-09 | 49747 | XXL_VLDL_L | Chylomicrons and Extremely Large VLDL |
| 23483 | -0.0005757 | 2.00E-08 | 49747 | XXL_VLDL_PL | Chylomicrons and Extremely Large VLDL |
| 23484 | -0.0005836 | 1.81E-08 | 49747 | XXL_VLDL_C | Chylomicrons and Extremely Large VLDL |
| 23485 | -0.000581 | 2.41E-08 | 49747 | XXL_VLDL_CE | Chylomicrons and Extremely Large VLDL |
| 23486 | -0.0005599 | 6.39E-08 | 49747 | XXL_VLDL_FC | Chylomicrons and Extremely Large VLDL |
| 23487 | -8.48E-05 | 0.478327 | 49747 | XXL_VLDL_TG | Chylomicrons and Extremely Large VLDL |
| 23488 | -0.0006411 | 5.21E-10 | 49747 | XL_VLDL_P | Very Large VLDL |
| 23489 | -0.0006702 | 8.78E-11 | 49747 | XL_VLDL_L | Very Large VLDL |
| 23490 | 0.00035252 | 0.0054983 | 49747 | XL_VLDL_PL | Very Large VLDL |
| 23491 | -0.0006772 | 1.10E-10 | 49747 | XL_VLDL_C | Very Large VLDL |
| 23492 | -0.0006286 | 3.33E-08 | 49747 | XL_VLDL_CE | Very Large VLDL |
| 23493 | -0.0004908 | 2.63E-10 | 49747 | XL_VLDL_FC | Very Large VLDL |
| 23494 | -0.0006126 | 3.61E-09 | 49747 | XL_VLDL_TG | Very Large VLDL |
| 23495 | -0.00064 | 8.50E-10 | 49747 | L_VLDL_P | Large VLDL |
| 23496 | -0.0006696 | 1.63E-10 | 49747 | L_VLDL_L | Large VLDL |
| 23497 | -0.0002125 | 0.0928207 | 49747 | L_VLDL_PL | Large VLDL |
| 23498 | -0.0006638 | 4.37E-10 | 49747 | L_VLDL_C | Large VLDL |
| 23499 | -0.0006304 | 5.48E-09 | 49747 | L_VLDL_CE | Large VLDL |
| 23500 | -0.0006833 | 6.42E-11 | 49747 | L_VLDL_FC | Large VLDL |
| 23501 | -0.0006154 | 4.29E-09 | 49747 | L_VLDL_TG | Large VLDL |
| 23502 | -0.0004454 | 3.47E-05 | 49747 | M_VLDL_P | Medium VLDL |
| 23503 | -0.0005089 | 2.25E-06 | 49747 | M_VLDL_L | Medium VLDL |
| 23504 | -0.0004313 | 6.22E-05 | 49747 | M_VLDL_PL | Medium VLDL |
| 23505 | -0.0002261 | 0.0324113 | 49747 | M_VLDL_C | Medium VLDL |
| 23506 | -1.21E-04 | 0.2480803 | 49747 | M_VLDL_CE | Medium VLDL |
| 23507 | -0.0003673 | 0.0006292 | 49747 | M_VLDL_FC | Medium VLDL |
| 23508 | -0.0005856 | 3.65E-08 | 49747 | M_VLDL_TG | Medium VLDL |
| 23509 | -0.0005709 | 1.39E-07 | 49747 | S_VLDL_P | Small VLDL |
| 23510 | -0.0005589 | 2.72E-07 | 49747 | S_VLDL_L | Small VLDL |
| 23511 | -0.0004622 | 2.12E-05 | 49747 | S_VLDL_PL | Small VLDL |
| 23512 | -0.000458 | 2.37E-05 | 49747 | S_VLDL_C | Small VLDL |
| 23513 | -0.0005103 | 2.89E-06 | 49747 | S_VLDL_CE | Small VLDL |
| 23514 | -0.0003578 | 0.0009012 | 49747 | S_VLDL_FC | Small VLDL |
| 23515 | -0.0005717 | 9.27E-08 | 49747 | S_VLDL_TG | Small VLDL |
| 23516 | -0.0002669 | 0.0123822 | 49747 | XS_VLDL_P | Very Small VLDL |
| 23517 | -0.0002563 | 0.0163404 | 49747 | XS_VLDL_L | Very Small VLDL |
| 23518 | -0.0003448 | 0.0014498 | 49747 | XS_VLDL_PL | Very Small VLDL |
| 23519 | -6.29E-05 | 0.540146 | 49747 | XS_VLDL_C | Very Small VLDL |
| 23520 | 1.26E-05 | 0.9011834 | 49747 | XS_VLDL_CE | Very Small VLDL |
| 23521 | -0.0002374 | 0.0253567 | 49747 | XS_VLDL_FC | Very Small VLDL |
| 23522 | -0.0004784 | 9.39E-06 | 49747 | XS_VLDL_TG | Very Small VLDL |
| 23523 | -1.19E-04 | 0.2494185 | 49747 | IDL_P | IDL |
| 23524 | 1.56E-04 | 0.1181717 | 49747 | IDL_L | IDL |
| 23525 | 8.83E-05 | 0.3808814 | 49747 | IDL_PL | IDL |
| 23526 | 0.00022067 | 0.0257535 | 49747 | IDL_C | IDL |
| 23527 | 0.00022167 | 0.0255503 | 49747 | IDL_CE | IDL |
| 23528 | 0.00020299 | 0.0427929 | 49747 | IDL_FC | IDL |
| 23529 | -0.0003923 | 0.0002949 | 49747 | IDL_TG | IDL |
| 23530 | -0.0002366 | 0.0257532 | 49747 | L_LDL_P | Large LDL |
| 23531 | 5.01E-05 | 0.6339265 | 49747 | L_LDL_L | Large LDL |
| 23532 | 4.46E-05 | 0.6726301 | 49747 | L_LDL_PL | Large LDL |
| 23533 | 8.88E-05 | 0.3977714 | 49747 | L_LDL_C | Large LDL |
| 23534 | 4.95E-05 | 0.6406147 | 49747 | L_LDL_CE | Large LDL |
| 23535 | 1.76E-04 | 0.0875594 | 49747 | L_LDL_FC | Large LDL |
| 23536 | -0.0003848 | 0.0003635 | 49747 | L_LDL_TG | Large LDL |
| 23537 | -0.0003018 | 0.0049538 | 49747 | M_LDL_P | Medium LDL |
| 23538 | -0.0002434 | 0.0240928 | 49747 | M_LDL_L | Medium LDL |
| 23539 | -0.0002367 | 0.029105 | 49747 | M_LDL_PL | Medium LDL |
| 23540 | -0.0002167 | 0.044281 | 49747 | M_LDL_C | Medium LDL |
| 23541 | -0.0002927 | 0.0066822 | 49747 | M_LDL_CE | Medium LDL |
| 23542 | -1.82E-05 | 0.8645571 | 49747 | M_LDL_FC | Medium LDL |
| 23543 | -0.0004473 | 2.61E-05 | 49747 | M_LDL_TG | Medium LDL |
| 23544 | -0.0003593 | 0.0007284 | 49747 | S_LDL_P | Small LDL |
| 23545 | -0.0002973 | 0.0055486 | 49747 | S_LDL_L | Small LDL |
| 23546 | -0.0002873 | 0.0068324 | 49747 | S_LDL_PL | Small LDL |
| 23547 | -0.0002499 | 0.0189799 | 49747 | S_LDL_C | Small LDL |
| 23548 | -0.0003058 | 0.0043582 | 49747 | S_LDL_CE | Small LDL |
| 23549 | -4.79E-05 | 0.6252803 | 49747 | S_LDL_FC | Small LDL |
| 23550 | -0.0005284 | 4.53E-07 | 49747 | S_LDL_TG | Small LDL |
| 23551 | 0.00067381 | 2.76E-11 | 49747 | XL_HDL_P | Very Large HDL |
| 23552 | 0.00069916 | 2.31E-12 | 49747 | XL_HDL_L | Very Large HDL |
| 23553 | 0.00054217 | 1.37E-07 | 49747 | XL_HDL_PL | Very Large HDL |
| 23554 | 0.00069089 | 2.57E-12 | 49747 | XL_HDL_C | Very Large HDL |
| 23555 | 0.00057851 | 5.50E-08 | 49747 | XL_HDL_CE | Very Large HDL |
| 23556 | 0.00032134 | 0.0083656 | 49747 | XL_HDL_FC | Very Large HDL |
| 23557 | -1.18E-04 | 0.3302193 | 49747 | XL_HDL_TG | Very Large HDL |
| 23558 | 0.00090118 | 3.25E-20 | 49747 | L_HDL_P | Large HDL |
| 23559 | 0.00094311 | 4.02E-23 | 49747 | L_HDL_L | Large HDL |
| 23560 | 0.0009301 | 6.62E-22 | 49747 | L_HDL_PL | Large HDL |
| 23561 | 0.00093067 | 1.93E-22 | 49747 | L_HDL_C | Large HDL |
| 23562 | 0.00093377 | 2.62E-22 | 49747 | L_HDL_CE | Large HDL |
| 23563 | 0.00080376 | 2.98E-17 | 49747 | L_HDL_FC | Large HDL |
| 23564 | 1.81E-04 | 0.1208746 | 49747 | L_HDL_TG | Large HDL |
| 23565 | 8.15E-05 | 0.5613206 | 49747 | M_HDL_P | Medium HDL |
| 23566 | 0.00085928 | 1.33E-17 | 49747 | M_HDL_L | Medium HDL |
| 23567 | 0.0007872 | 1.05E-14 | 49747 | M_HDL_PL | Medium HDL |
| 23568 | 0.00095291 | 1.22E-21 | 49747 | M_HDL_C | Medium HDL |
| 23569 | 0.00095471 | 1.21E-21 | 49747 | M_HDL_CE | Medium HDL |
| 23570 | 0.00087882 | 1.53E-18 | 49747 | M_HDL_FC | Medium HDL |
| 23571 | -0.0002283 | 0.0372337 | 49747 | M_HDL_TG | Medium HDL |
| 23572 | 0.00029268 | 0.008513 | 49747 | S_HDL_P | Small HDL |
| 23573 | 0.00026705 | 0.0147954 | 49747 | S_HDL_L | Small HDL |
| 23574 | 0.00031407 | 0.00377 | 49747 | S_HDL_PL | Small HDL |
| 23575 | 0.00038561 | 0.0005177 | 49747 | S_HDL_C | Small HDL |
| 23576 | 0.0003648 | 0.0011576 | 49747 | S_HDL_CE | Small HDL |
| 23577 | 0.0003636 | 0.0008371 | 49747 | S_HDL_FC | Small HDL |
| 23578 | -0.0005672 | 5.93E-08 | 49747 | S_HDL_TG | Small HDL |
| 30000 | -0.0005055 | 1.33E-09 | 88326 | White blood cell (leukocyte) count | White blood cell |
| 30010 | -0.0003568 | 9.40E-07 | 88328 | Red blood cell (erythrocyte) count | Red blood cell |
| 30020 | -1.57E-04 | 0.0205286 | 88327 | Haemoglobin concentration | Red blood cell |
| 30030 | -1.59E-04 | 0.023117 | 88328 | Haematocrit percentage | Red blood cell |
| 30040 | 0.00036332 | 7.87E-06 | 88328 | Mean corpuscular volume | Red blood cell |
| 30050 | 0.00030928 | 1.25E-04 | 88326 | Mean corpuscular haemoglobin | Red blood cell |
| 30060 | 1.63E-05 | 0.852454 | 88326 | Mean corpuscular haemoglobin concentration | Red blood cell |
| 30070 | -1.63E-04 | 0.0533086 | 88328 | Red blood cell (erythrocyte) distribution width | Red blood cell |
| 30080 | -1.97E-04 | 0.0181089 | 88328 | Platelet count | Platelet |
| 30090 | -2.09E-04 | 0.011132 | 88327 | Platelet crit | Platelet |
| 30100 | 6.95E-05 | 0.4310849 | 88327 | Mean platelet (thrombocyte) volume | Platelet |
| 30110 | -1.14E-04 | 0.1899864 | 88327 | Platelet distribution width | Platelet |
| 30120 | -0.0001971 | 0.0190861 | 88148 | Lymphocyte count | White blood cell |
| 30130 | -0.000366 | 8.20E-06 | 88148 | Monocyte count | White blood cell |
| 30140 | -0.000454 | 7.99E-08 | 88148 | Neutrophill count | White blood cell |
| 30150 | -0.0002193 | 0.0110422 | 88148 | Eosinophill count | White blood cell |
| 30160 | -8.42E-05 | 0.3163966 | 88148 | Basophill count | White blood cell |
| 30170 | -5.46E-05 | 0.5075582 | 88147 | Nucleated red blood cell count | Red blood cell |
| 30180 | 0.00026191 | 0.0023017 | 88150 | Lymphocyte percentage | White blood cell |
| 30190 | -4.16E-05 | 0.6214462 | 88150 | Monocyte percentage | White blood cell |
| 30200 | -1.60E-04 | 0.0947506 | 88150 | Neutrophill percentage | White blood cell |
| 30210 | -1.03E-04 | 0.2426788 | 88150 | Eosinophill percentage | White blood cell |
| 30220 | 1.19E-05 | 0.8903612 | 88150 | Basophill percentage | White blood cell |
| 30230 | -3.49E-05 | 0.6616111 | 88146 | Nucleated red blood cell percentage | Red blood cell |
| 30240 | -2.00E-04 | 0.0081543 | 86572 | Reticulocyte percentage | Red blood cell |
| 30250 | -0.0002693 | 0.000704 | 86572 | Reticulocyte count | Red blood cell |
| 30260 | 0.0002277 | 0.0090892 | 86572 | Mean reticulocyte volume | Red blood cell |
| 30270 | 0.0003894 | 4.89E-06 | 86572 | Mean sphered cell volume | Red blood cell |
| 30280 | -0.0004214 | 6.31E-07 | 86572 | Immature reticulocyte fraction | Red blood cell |
| 30290 | -0.0002606 | 1.23E-06 | 86572 | High light scatter reticulocyte percentage | Red blood cell |
| 30300 | -0.0004402 | 1.94E-08 | 86572 | High light scatter reticulocyte count | Red blood cell |
| 30600 | 1.91E-04 | 0.033263 | 79398 | Albumin | Liver function |
| 30610 | -0.0002306 | 0.0030375 | 86722 | Alkaline phosphatase | Bone and joint |
| 30620 | -0.0001944 | 0.0117457 | 86702 | Alanine aminotransferase | Liver function |
| 30630 | 0.00100284 | 4.49E-37 | 78914 | Apolipoprotein A | Immunometabolism |
| 30640 | -0.0003708 | 4.41E-06 | 86362 | Apolipoprotein B | Immunometabolism |
| 30650 | 0.00077661 | 3.27E-22 | 86422 | Aspartate aminotransferase | Liver function |
| 30660 | 0.00028271 | 0.0007633 | 74354 | Direct bilirubin | Liver function |
| 30670 | 0.00047901 | 1.27E-09 | 86666 | Urea | Renal function |
| 30680 | 1.81E-04 | 0.0434101 | 79386 | Calcium | Bone and joint |
| 30690 | 3.72E-05 | 0.6316099 | 86722 | Cholesterol | Immunometabolism |
| 30700 | -4.64E-05 | 0.4404771 | 86682 | Creatinine | Renal function |
| 30710 | -0.0003016 | 0.000102 | 86541 | C-reactive protein | Immunometabolism |
| 30720 | -0.0004111 | 1.20E-10 | 86717 | Cystatin C | Renal function |
| 30730 | -1.24E-04 | 0.0840262 | 86691 | Gamma glutamyltransferase | Liver function |
| 30740 | -1.89E-04 | 0.0125651 | 79318 | Glucose | Endocrine |
| 30750 | -0.0002004 | 0.0033461 | 86095 | Glycated haemoglobin (HbA1c) | Immunometabolism |
| 30760 | 0.00104837 | 2.99E-43 | 79375 | HDL cholesterol | Immunometabolism |
| 30770 | 1.97E-05 | 0.8133953 | 86256 | IGF-1 | Endocrine |
| 30780 | -0.0002122 | 0.0068431 | 86574 | LDL direct | Immunometabolism |
| 30790 | 1.27E-04 | 0.1881321 | 69533 | Lipoprotein A | Endocrine |
| 30800 | 0.00045163 | 0.0268948 | 15127 | Oestradiol | Endocrine |
| 30810 | 1.66E-04 | 0.0583158 | 79236 | Phosphate | Renal function |
| 30820 | -0.000189 | 0.5422953 | 7398 | Rheumatoid factor | Immunometabolism |
| 30830 | 0.00038205 | 2.05E-06 | 78611 | SHBG | Endocrine |
| 30840 | 0.00030738 | 0.0004128 | 86377 | Total bilirubin | Liver function |
| 30850 | 0.00021316 | 1.72E-08 | 78316 | Testosterone | Endocrine |
| 30860 | -8.72E-05 | 0.3456229 | 79336 | Total protein | Immunometabolism |
| 30870 | -0.0005729 | 1.11E-13 | 86664 | Triglycerides | Immunometabolism |
| 30880 | -9.31E-05 | 0.1603866 | 86638 | Urate | Renal function |
| 30890 | 0.00123762 | 3.34E-48 | 83048 | Vitamin D | Bone and joint |

| **Table S10. Associations of repeated measurements blood and metabolomic biomarkers with amplitude.** | | | | | |
| --- | --- | --- | --- | --- | --- |
| **Field ID** | **Beta** | **P Value** | **Sample size** | **Biomarker** | **Group** |
| 20280 | -0.0002778 | 1.07E-01 | 5671 | Glucose-lactate | Glycolysis related metabolites |
| 20281 | -2.724E-05 | 8.93E-01 | 5671 | Spectrometer-corrected alanine | Amino acids |
| 23400 | 0.00043007 | 2.16E-02 | 5671 | Total_C | Cholesterol |
| 23401 | 0.00013291 | 4.99E-01 | 5671 | Non_HDL_C | Cholesterol |
| 23402 | 2.2397E-05 | 9.09E-01 | 5671 | Remnant_C | Cholesterol |
| 23403 | -0.0002695 | 1.86E-01 | 5671 | VLDL Cholesterol | Cholesterol |
| 23404 | 0.00021768 | 2.67E-01 | 5671 | Clinical_LDL_C | Cholesterol |
| 23405 | 0.00023248 | 2.40E-01 | 5671 | LDL_C | Cholesterol |
| 23406 | 0.00092362 | 2.71E-07 | 5671 | HDL_C | Cholesterol |
| 23407 | -0.0004516 | 2.78E-02 | 5671 | Total_TG | Triglycerides |
| 23408 | -0.0004597 | 2.39E-02 | 5671 | VLDL_TG | Triglycerides |
| 23409 | -0.0003786 | 7.00E-02 | 5671 | LDL_TG | Triglycerides |
| 23410 | -0.0002317 | 2.72E-01 | 5671 | HDL_TG | Triglycerides |
| 23411 | 0.00047752 | 1.12E-02 | 5671 | Total_PL | Phospholipids |
| 23412 | -0.0004087 | 4.81E-02 | 5671 | VLDL_PL | Phospholipids |
| 23413 | 0.00016491 | 4.07E-01 | 5671 | LDL_PL | Phospholipids |
| 23414 | 0.00083168 | 6.07E-06 | 5671 | HDL_PL | Phospholipids |
| 23415 | 0.0004822 | 9.67E-03 | 5671 | Total_CE | Cholesteryl Esters |
| 23416 | -0.0002024 | 3.17E-01 | 5671 | VLDL_CE | Cholesteryl Esters |
| 23417 | 0.00019439 | 3.30E-01 | 5671 | LDL_CE | Cholesteryl Esters |
| 23418 | 0.00094589 | 1.79E-07 | 5671 | HDL_CE | Cholesteryl Esters |
| 23419 | 0.00028935 | 1.27E-01 | 5671 | Total_FC | Free Cholesterol |
| 23420 | -0.0003531 | 8.71E-02 | 5671 | VLDL_FC | Free Cholesterol |
| 23421 | 0.00032566 | 9.28E-02 | 5671 | LDL_FC | Free Cholesterol |
| 23422 | 0.00080587 | 6.90E-06 | 5671 | HDL_FC | Free Cholesterol |
| 23423 | 0.00024738 | 2.08E-01 | 5671 | Total_L | Total Lipids |
| 23424 | -0.0004282 | 3.75E-02 | 5671 | VLDL_L | Total Lipids |
| 23425 | 0.00018646 | 3.48E-01 | 5671 | LDL_L | Total Lipids |
| 23426 | 0.00087097 | 1.47E-06 | 5671 | HDL_L | Total Lipids |
| 23427 | 0.00084267 | 1.10E-05 | 5671 | Total_P | Particle Concentrations |
| 23428 | -0.0003194 | 1.24E-01 | 5671 | VLDL_P | Particle Concentrations |
| 23429 | -1.751E-05 | 9.30E-01 | 5671 | LDL_P | Particle Concentrations |
| 23430 | 0.00089146 | 3.87E-06 | 5671 | HDL_P | Particle Concentrations |
| 23431 | -0.0004736 | 1.43E-02 | 5671 | VLDL_size | Lipoprotein particle size |
| 23432 | 0.00034937 | 8.03E-02 | 5671 | LDL_size | Lipoprotein particle size |
| 23433 | 0.00061819 | 5.41E-04 | 5671 | HDL_size | Lipoprotein particle size |
| 23434 | 0.00051536 | 7.16E-03 | 5671 | Phosphoglycerides | Other lipids |
| 23436 | 0.00057031 | 2.49E-03 | 5671 | Cholines | Other lipids |
| 23437 | 0.00061143 | 1.23E-03 | 5671 | Phosphatidylc | Other lipids |
| 23438 | 0.00037581 | 4.12E-02 | 5671 | Sphingomyelins | Other lipids |
| 23439 | -4.07E-05 | 8.38E-01 | 5671 | ApoB | Apolipoproteins |
| 23440 | 0.00087884 | 2.29E-06 | 5671 | ApoA1 | Apolipoproteins |
| 23442 | -3.041E-07 | 9.99E-01 | 5671 | Total_FA | Fatty acids |
| 23443 | 0.00024407 | 2.26E-01 | 5671 | Unsaturation | Fatty acids |
| 23444 | -0.0003607 | 7.32E-02 | 5671 | Omega_3 | Fatty acids |
| 23445 | 0.00044499 | 2.39E-02 | 5671 | Omega_6 | Fatty acids |
| 23446 | 0.00027528 | 1.62E-01 | 5671 | PUFA | Fatty acids |
| 23447 | -0.0002393 | 2.47E-01 | 5671 | MUFA | Fatty acids |
| 23448 | -5.854E-05 | 7.74E-01 | 5671 | SFA | Fatty acids |
| 23449 | 0.00050346 | 1.04E-02 | 5671 | LA | Fatty acids |
| 23450 | -0.0001766 | 3.69E-01 | 5671 | DHA | Fatty acids |
| 23460 | -0.0001636 | 4.29E-01 | 5671 | Alanine | Amino acids |
| 23461 | 0.00027657 | 1.81E-01 | 5671 | Glutamine | Amino acids |
| 23462 | 0.00013385 | 5.07E-01 | 5671 | Glycine | Amino acids |
| 23463 | 5.3358E-06 | 9.78E-01 | 5671 | Histidine | Amino acids |
| 23464 | -0.0001006 | 6.22E-01 | 5671 | Total_BCAA | Amino acids |
| 23465 | -0.0001892 | 3.55E-01 | 5671 | Isoleucine | Amino acids |
| 23466 | 4.9394E-05 | 8.07E-01 | 5671 | Leucine | Amino acids |
| 23467 | -0.0001295 | 5.25E-01 | 5671 | Valine | Amino acids |
| 23468 | 9.2988E-05 | 6.70E-01 | 5671 | Phenylalanine | Amino acids |
| 23469 | -0.0002248 | 2.68E-01 | 5671 | Tyrosine | Amino acids |
| 23470 | 0.00011997 | 4.78E-01 | 5671 | Glucose | Glycolysis related metabolites |
| 23471 | -0.0012176 | 2.66E-09 | 5671 | Lactate | Glycolysis related metabolites |
| 23472 | -0.001069 | 2.24E-07 | 5671 | Pyruvate | Glycolysis related metabolites |
| 23473 | 0.00016873 | 4.26E-01 | 5671 | Citrate | Glycolysis related metabolites |
| 23474 | -0.000184 | 3.86E-01 | 5671 | bOHbutyrate | Ketone bodies |
| 23475 | -0.0002451 | 2.98E-01 | 5671 | Acetate | Ketone bodies |
| 23476 | -1.916E-05 | 9.25E-01 | 5671 | Acetoacetate | Ketone bodies |
| 23477 | 4.7064E-05 | 8.25E-01 | 5671 | Acetone | Ketone bodies |
| 23478 | -1.967E-05 | 9.29E-01 | 5671 | Creatinine | Fluid balance |
| 23479 | 0.00014264 | 4.91E-01 | 5671 | Albumin | Fluid balance |
| 23480 | -0.0006801 | 6.77E-04 | 5671 | GlycA | Inflammation |
| 23481 | -0.0004993 | 1.28E-02 | 5671 | XXL_VLDL_P | Chylomicrons and Extremely Large VLDL |
| 23482 | -0.0005246 | 8.73E-03 | 5671 | XXL_VLDL_L | Chylomicrons and Extremely Large VLDL |
| 23483 | -0.0005192 | 9.49E-03 | 5671 | XXL_VLDL_PL | Chylomicrons and Extremely Large VLDL |
| 23484 | -0.0004955 | 1.41E-02 | 5671 | XXL_VLDL_C | Chylomicrons and Extremely Large VLDL |
| 23485 | -0.0004814 | 1.76E-02 | 5671 | XXL_VLDL_CE | Chylomicrons and Extremely Large VLDL |
| 23486 | -0.0004949 | 1.39E-02 | 5671 | XXL_VLDL_FC | Chylomicrons and Extremely Large VLDL |
| 23487 | -0.0005034 | 1.18E-02 | 5671 | XXL_VLDL_TG | Chylomicrons and Extremely Large VLDL |
| 23488 | -0.0005114 | 1.17E-02 | 5671 | XL_VLDL_P | Very Large VLDL |
| 23489 | -0.000515 | 1.09E-02 | 5671 | XL_VLDL_L | Very Large VLDL |
| 23490 | -0.0005326 | 8.71E-03 | 5671 | XL_VLDL_PL | Very Large VLDL |
| 23491 | -0.0004855 | 1.81E-02 | 5671 | XL_VLDL_C | Very Large VLDL |
| 23492 | -0.0004346 | 3.52E-02 | 5671 | XL_VLDL_CE | Very Large VLDL |
| 23493 | -0.0005188 | 1.10E-02 | 5671 | XL_VLDL_FC | Very Large VLDL |
| 23494 | -0.0004933 | 1.42E-02 | 5671 | XL_VLDL_TG | Very Large VLDL |
| 23495 | -0.0004718 | 2.14E-02 | 5671 | L_VLDL_P | Large VLDL |
| 23496 | -0.0004587 | 2.51E-02 | 5671 | L_VLDL_L | Large VLDL |
| 23497 | -0.0005018 | 1.41E-02 | 5671 | L_VLDL_PL | Large VLDL |
| 23498 | -0.0004646 | 2.48E-02 | 5671 | L_VLDL_C | Large VLDL |
| 23499 | -0.0004172 | 4.53E-02 | 5671 | L_VLDL_CE | Large VLDL |
| 23500 | -0.0004925 | 1.62E-02 | 5671 | L_VLDL_FC | Large VLDL |
| 23501 | -0.0004108 | 0.044487 | 5671 | L_VLDL_TG | Large VLDL |
| 23502 | -0.0001848 | 0.3699293 | 5671 | M_VLDL_P | Medium VLDL |
| 23503 | -0.000239 | 0.2478241 | 5671 | M_VLDL_L | Medium VLDL |
| 23504 | -0.0001749 | 0.3934738 | 5671 | M_VLDL_PL | Medium VLDL |
| 23505 | 1.0922E-05 | 0.9559709 | 5671 | M_VLDL_C | Medium VLDL |
| 23506 | 0.00011521 | 0.5516284 | 5671 | M_VLDL_CE | Medium VLDL |
| 23507 | -0.0001265 | 0.5335282 | 5671 | M_VLDL_FC | Medium VLDL |
| 23508 | -0.0003514 | 0.0900324 | 5671 | M_VLDL_TG | Medium VLDL |
| 23509 | -0.0003543 | 0.0907235 | 5671 | S_VLDL_P | Small VLDL |
| 23510 | -0.0003353 | 0.1087356 | 5671 | S_VLDL_L | Small VLDL |
| 23511 | -0.0002152 | 0.2982564 | 5671 | S_VLDL_PL | Small VLDL |
| 23512 | -0.0002533 | 0.2187084 | 5671 | S_VLDL_C | Small VLDL |
| 23513 | -0.0003337 | 0.1075664 | 5671 | S_VLDL_CE | Small VLDL |
| 23514 | -0.0001119 | 0.5823152 | 5671 | S_VLDL_FC | Small VLDL |
| 23515 | -0.0003878 | 0.0623598 | 5671 | S_VLDL_TG | Small VLDL |
| 23516 | -0.0001952 | 0.3270255 | 5671 | XS_VLDL_P | Very Small VLDL |
| 23517 | -0.0001799 | 0.3625743 | 5671 | XS_VLDL_L | Very Small VLDL |
| 23518 | -0.0003014 | 0.1322811 | 5671 | XS_VLDL_PL | Very Small VLDL |
| 23519 | -4.382E-06 | 0.9816252 | 5671 | XS_VLDL_C | Very Small VLDL |
| 23520 | 7.0242E-05 | 0.7081711 | 5671 | XS_VLDL_CE | Very Small VLDL |
| 23521 | -0.0001843 | 0.3484785 | 5671 | XS_VLDL_FC | Very Small VLDL |
| 23522 | -0.0004351 | 0.0375108 | 5671 | XS_VLDL_TG | Very Small VLDL |
| 23523 | 3.7782E-05 | 0.8450378 | 5671 | IDL_P | IDL |
| 23524 | 0.00027183 | 0.1434626 | 5671 | IDL_L | IDL |
| 23525 | 0.00021358 | 0.2500906 | 5671 | IDL_PL | IDL |
| 23526 | 0.00034016 | 0.0656343 | 5671 | IDL_C | IDL |
| 23527 | 0.00036943 | 0.0455897 | 5671 | IDL_CE | IDL |
| 23528 | 0.00025007 | 0.1787978 | 5671 | IDL_FC | IDL |
| 23529 | -0.0003715 | 0.0750948 | 5671 | IDL_TG | IDL |
| 23530 | 2.171E-05 | 0.9128132 | 5671 | L_LDL_P | Large LDL |
| 23531 | 0.00027412 | 0.1608748 | 5671 | L_LDL_L | Large LDL |
| 23532 | 0.00024612 | 0.2085751 | 5671 | L_LDL_PL | Large LDL |
| 23533 | 0.00032109 | 0.09873 | 5671 | L_LDL_C | Large LDL |
| 23534 | 0.00030057 | 0.1255845 | 5671 | L_LDL_CE | Large LDL |
| 23535 | 0.00036806 | 0.0531684 | 5671 | L_LDL_FC | Large LDL |
| 23536 | -0.0003567 | 0.0876677 | 5671 | L_LDL_TG | Large LDL |
| 23537 | -6.272E-05 | 0.7588237 | 5671 | M_LDL_P | Medium LDL |
| 23538 | 4.67E-05 | 0.8185862 | 5671 | M_LDL_L | Medium LDL |
| 23539 | 7.0568E-05 | 0.729337 | 5671 | M_LDL_PL | Medium LDL |
| 23540 | 6.9911E-05 | 0.7306769 | 5671 | M_LDL_C | Medium LDL |
| 23541 | -1.577E-06 | 0.9938418 | 5671 | M_LDL_CE | Medium LDL |
| 23542 | 0.00025712 | 0.1973817 | 5671 | M_LDL_FC | Medium LDL |
| 23543 | -0.0003751 | 0.0720739 | 5671 | M_LDL_TG | Medium LDL |
| 23544 | -1.11E-04 | 0.5832838 | 5671 | S_LDL_P | Small LDL |
| 23545 | -7.788E-06 | 0.9691986 | 5671 | S_LDL_L | Small LDL |
| 23546 | -1.33E-05 | 0.9473077 | 5671 | S_LDL_PL | Small LDL |
| 23547 | 4.3456E-05 | 0.8289603 | 5671 | S_LDL_C | Small LDL |
| 23548 | -1.81E-05 | 0.9287122 | 5671 | S_LDL_CE | Small LDL |
| 23549 | 0.00019994 | 0.3158091 | 5671 | S_LDL_FC | Small LDL |
| 23550 | -4.19E-04 | 0.043048 | 5671 | S_LDL_TG | Small LDL |
| 23551 | 0.00048964 | 0.0069298 | 5671 | XL_HDL_P | Very Large HDL |
| 23552 | 5.01E-04 | 0.0054864 | 5671 | XL_HDL_L | Very Large HDL |
| 23553 | 0.00048008 | 0.0085056 | 5671 | XL_HDL_PL | Very Large HDL |
| 23554 | 0.0005244 | 0.0037401 | 5671 | XL_HDL_C | Very Large HDL |
| 23555 | 0.00057632 | 0.0013879 | 5671 | XL_HDL_CE | Very Large HDL |
| 23556 | 0.00030064 | 0.1068501 | 5671 | XL_HDL_FC | Very Large HDL |
| 23557 | -1.08E-04 | 0.6030787 | 5671 | XL_HDL_TG | Very Large HDL |
| 23558 | 0.00073727 | 4.356E-05 | 5671 | L_HDL_P | Large HDL |
| 23559 | 0.00078372 | 8.81E-06 | 5671 | L_HDL_L | Large HDL |
| 23560 | 0.00076868 | 1.821E-05 | 5671 | L_HDL_PL | Large HDL |
| 23561 | 0.00077484 | 1.301E-05 | 5671 | L_HDL_C | Large HDL |
| 23562 | 7.84E-04 | 1.144E-05 | 5671 | L_HDL_CE | Large HDL |
| 23563 | 0.00069729 | 0.0001014 | 5671 | L_HDL_FC | Large HDL |
| 23564 | 0.00014277 | 0.4829516 | 5671 | L_HDL_TG | Large HDL |
| 23565 | 0.00089136 | 2.913E-06 | 5671 | M_HDL_P | Medium HDL |
| 23566 | 8.65E-04 | 5.515E-06 | 5671 | M_HDL_L | Medium HDL |
| 23567 | 0.000804 | 3.082E-05 | 5671 | M_HDL_PL | Medium HDL |
| 23568 | 0.0009471 | 5.394E-07 | 5671 | M_HDL_C | Medium HDL |
| 23569 | 0.00095848 | 4.673E-07 | 5671 | M_HDL_CE | Medium HDL |
| 23570 | 8.75E-04 | 2.916E-06 | 5671 | M_HDL_FC | Medium HDL |
| 23571 | -0.0001946 | 0.3543016 | 5671 | M_HDL_TG | Medium HDL |
| 23572 | 5.56E-04 | 0.0073346 | 5671 | S_HDL_P | Small HDL |
| 23573 | 4.88E-04 | 0.0179652 | 5671 | S_HDL_L | Small HDL |
| 23574 | 4.94E-04 | 0.0161496 | 5671 | S_HDL_PL | Small HDL |
| 23575 | 6.40E-04 | 0.0020325 | 5671 | S_HDL_C | Small HDL |
| 23576 | 6.32E-04 | 0.0023904 | 5671 | S_HDL_CE | Small HDL |
| 23577 | 5.74E-04 | 0.0047583 | 5671 | S_HDL_FC | Small HDL |
| 23578 | -0.0004819 | 0.0181402 | 5671 | S_HDL_TG | Small HDL |
| 30000 | -3.21E-04 | 0.062557 | 6555 | White blood cell (leukocyte) count | White blood cell |
| 30010 | -2.67E-04 | 0.0726453 | 6555 | Red blood cell (erythrocyte) count | Red blood cell |
| 30020 | -1.01E-04 | 0.4671816 | 6556 | Haemoglobin concentration | Red blood cell |
| 30030 | -1.28E-04 | 0.3700933 | 6556 | Haematocrit percentage | Red blood cell |
| 30040 | 2.77E-04 | 0.0932619 | 6556 | Mean corpuscular volume | Red blood cell |
| 30050 | 2.43E-04 | 0.1316906 | 6556 | Mean corpuscular haemoglobin | Red blood cell |
| 30060 | 3.44E-05 | 0.8300711 | 6556 | Mean corpuscular haemoglobin concentration | Red blood cell |
| 30070 | -1.25E-05 | 0.941086 | 6556 | Red blood cell (erythrocyte) distribution width | Red blood cell |
| 30080 | 6.48E-05 | 0.6949932 | 6556 | Platelet count | Platelet |
| 30090 | -3.13E-05 | 0.8689706 | 4757 | Platelet crit | Platelet |
| 30100 | -1.45E-04 | 0.4052407 | 6555 | Mean platelet (thrombocyte) volume | Platelet |
| 30110 | -8.11E-05 | 0.6895532 | 4757 | Platelet distribution width | Platelet |
| 30120 | -3.22E-04 | 0.0612301 | 6550 | Lymphocyte count | White blood cell |
| 30130 | -2.00E-04 | 0.2451665 | 6550 | Monocyte count | White blood cell |
| 30140 | -2.03E-04 | 0.2384655 | 6550 | Neutrophill count | White blood cell |
| 30150 | -3.20E-04 | 0.0555099 | 6550 | Eosinophill count | White blood cell |
| 30160 | -2.41E-04 | 0.1670631 | 6550 | Basophill count | White blood cell |
| 30170 | -7.12E-05 | 0.7018813 | 4747 | Nucleated red blood cell count | Red blood cell |
| 30180 | -1.16E-04 | 0.4996125 | 6551 | Lymphocyte percentage | White blood cell |
| 30190 | -5.53E-05 | 0.753049 | 6551 | Monocyte percentage | White blood cell |
| 30200 | 1.08E-04 | 0.5369733 | 6551 | Neutrophill percentage | White blood cell |
| 30210 | -1.44E-04 | 0.4037871 | 6551 | Eosinophill percentage | White blood cell |
| 30220 | -8.10E-05 | 0.6437966 | 6551 | Basophill percentage | White blood cell |
| 30230 | -8.61E-05 | 0.647228 | 4747 | Nucleated red blood cell percentage | Red blood cell |
| 30240 | -4.42E-04 | 0.0087828 | 6529 | Reticulocyte percentage | Red blood cell |
| 30250 | -4.56E-04 | 0.0057994 | 6529 | Reticulocyte count | Red blood cell |
| 30260 | 2.87E-04 | 0.0947517 | 6529 | Mean reticulocyte volume | Red blood cell |
| 30270 | 3.51E-04 | 0.0770473 | 4755 | Mean sphered cell volume | Red blood cell |
| 30280 | -6.68E-04 | 0.0006306 | 4755 | Immature reticulocyte fraction | Red blood cell |
| 30290 | -5.39E-04 | 0.0051056 | 4755 | High light scatter reticulocyte percentage | Red blood cell |
| 30300 | -5.20E-04 | 0.0067689 | 4755 | High light scatter reticulocyte count | Red blood cell |
| 30600 | -1.86E-05 | 0.9191064 | 5236 | Albumin | Liver function |
| 30610 | -3.40E-04 | 0.0556412 | 5995 | Alkaline phosphatase | Bone and joint |
| 30620 | -6.03E-05 | 0.7221162 | 5995 | Alanine aminotransferase | Liver function |
| 30630 | 0.00065246 | 7.079E-05 | 5190 | Apolipoprotein A | Immunometabolism |
| 30640 | -1.53E-04 | 0.384077 | 5973 | Apolipoprotein B | Immunometabolism |
| 30650 | 7.95E-04 | 1.476E-05 | 5972 | Aspartate aminotransferase | Liver function |
| 30660 | 6.85E-04 | 0.000188 | 5065 | Direct bilirubin | Liver function |
| 30670 | 2.47E-04 | 0.1474661 | 5990 | Urea | Renal function |
| 30680 | 6.37E-05 | 0.7375947 | 5236 | Calcium | Bone and joint |
| 30690 | 1.34E-04 | 0.415112 | 5998 | Cholesterol | Immunometabolism |
| 30700 | -1.93E-04 | 0.1684682 | 5987 | Creatinine | Renal function |
| 30710 | -3.25E-04 | 0.0531567 | 5986 | C-reactive protein | Immunometabolism |
| 30720 | -5.79E-04 | 0.0001376 | 5988 | Cystatin C | Renal function |
| 30730 | -1.56E-04 | 0.3526654 | 5995 | Gamma glutamyltransferase | Liver function |
| 30740 | -7.80E-06 | 0.9549573 | 5235 | Glucose | Endocrine |
| 30750 | -2.19E-04 | 0.1513836 | 4689 | Glycated haemoglobin (HbA1c) | Immunometabolism |
| 30760 | 7.68E-04 | 9.643E-07 | 5235 | HDL cholesterol | Immunometabolism |
| 30770 | -7.10E-05 | 0.6867966 | 5936 | IGF-1 | Endocrine |
| 30780 | -6.84E-05 | 0.687036 | 5991 | LDL direct | Immunometabolism |
| 30790 | -3.25E-04 | 0.1046329 | 4782 | Lipoprotein A | Endocrine |
| 30800 | 4.25E-04 | 0.2796499 | 659 | Oestradiol | Endocrine |
| 30810 | -9.25E-05 | 0.6090115 | 5220 | Phosphate | Renal function |
| 30820 | 3.06E-04 | 0.5633202 | 576 | Rheumatoid factor | Immunometabolism |
| 30830 | 5.65E-04 | 0.0006001 | 5151 | SHBG | Endocrine |
| 30840 | 7.17E-04 | 4.258E-05 | 5963 | Total bilirubin | Liver function |
| 30850 | 1.45E-04 | 0.0793338 | 5369 | Testosterone | Endocrine |
| 30860 | -1.74E-04 | 0.3510488 | 5234 | Total protein | Immunometabolism |
| 30870 | -6.62E-04 | 0.0001553 | 5987 | Triglycerides | Immunometabolism |
| 30880 | -1.22E-04 | 0.4010159 | 5985 | Urate | Renal function |
| 30890 | 8.69E-04 | 1.781E-06 | 5726 | Vitamin D | Bone and joint |

| **Table S11. Associations of repeated measurements Blood and Metabolomic Biomarkers with acrophase.** | | | | | |
| --- | --- | --- | --- | --- | --- |
| **Field ID** | **Beta** | **P Value** | **Sample size** | **Biomarker** | **Group** |
| 20280 | -0.0062621 | 0.4350337 | 5671 | Glucose-lactate | Glycolysis related metabolites |
| 20281 | 5.05E-03 | 0.5919733 | 5671 | Spectrometer-corrected alanine | Amino acids |
| 23400 | -0.0082274 | 0.3447877 | 5671 | Total_C | Cholesterol |
| 23401 | 3.61E-03 | 0.6933819 | 5671 | Non_HDL_C | Cholesterol |
| 23402 | 8.21E-03 | 0.3679769 | 5671 | Remnant_C | Cholesterol |
| 23403 | 0.02411449 | 1.10E-02 | 5671 | VLDL Cholesterol | Cholesterol |
| 23404 | -3.82E-03 | 0.6749539 | 5671 | Clinical_LDL_C | Cholesterol |
| 23405 | -8.43E-04 | 0.9270425 | 5671 | LDL_C | Cholesterol |
| 23406 | -0.0340037 | 4.75E-05 | 5671 | HDL_C | Cholesterol |
| 23407 | 0.04480258 | 2.65E-06 | 5671 | Total_TG | Triglycerides |
| 23408 | 0.0453009 | 1.69E-06 | 5671 | VLDL_TG | Triglycerides |
| 23409 | 0.03100349 | 1.42E-03 | 5671 | LDL_TG | Triglycerides |
| 23410 | 0.03318767 | 0.0007071 | 5671 | HDL_TG | Triglycerides |
| 23411 | -0.00276 | 0.7527625 | 5671 | Total_PL | Phospholipids |
| 23412 | 0.03541801 | 2.30E-04 | 5671 | VLDL_PL | Phospholipids |
| 23413 | 1.15E-03 | 0.9015144 | 5671 | LDL_PL | Phospholipids |
| 23414 | -0.0227389 | 7.88E-03 | 5671 | HDL_PL | Phospholipids |
| 23415 | -0.0108439 | 0.2111498 | 5671 | Total_CE | Cholesteryl Esters |
| 23416 | 0.01820319 | 0.0527999 | 5671 | VLDL_CE | Cholesteryl Esters |
| 23417 | 2.69E-03 | 0.7719159 | 5671 | LDL_CE | Cholesteryl Esters |
| 23418 | -0.0355842 | 2.45E-05 | 5671 | HDL_CE | Cholesteryl Esters |
| 23419 | -1.41E-03 | 0.8731035 | 5671 | Total_FC | Free Cholesterol |
| 23420 | 0.03111237 | 1.19E-03 | 5671 | VLDL_FC | Free Cholesterol |
| 23421 | -1.02E-02 | 0.2575673 | 5671 | LDL_FC | Free Cholesterol |
| 23422 | -0.026767 | 1.33E-03 | 5671 | HDL_FC | Free Cholesterol |
| 23423 | 8.69E-03 | 0.3421133 | 5671 | Total_L | Total Lipids |
| 23424 | 0.03978972 | 3.23E-05 | 5671 | VLDL_L | Total Lipids |
| 23425 | 1.53E-03 | 0.8682205 | 5671 | LDL_L | Total Lipids |
| 23426 | -0.0263814 | 1.73E-03 | 5671 | HDL_L | Total Lipids |
| 23427 | -0.0193753 | 2.99E-02 | 5671 | Total_P | Particle Concentrations |
| 23428 | 0.02968693 | 2.11E-03 | 5671 | VLDL_P | Particle Concentrations |
| 23429 | 0.00843369 | 0.3644959 | 5671 | LDL_P | Particle Concentrations |
| 23430 | -0.0218108 | 1.52E-02 | 5671 | HDL_P | Particle Concentrations |
| 23431 | 0.04404481 | 9.54E-07 | 5671 | VLDL_size | Lipoprotein particle size |
| 23432 | -0.0186989 | 0.0442136 | 5671 | LDL_size | Lipoprotein particle size |
| 23433 | -0.030733 | 2.18E-04 | 5671 | HDL_size | Lipoprotein particle size |
| 23434 | -0.0059096 | 5.08E-01 | 5671 | Phosphoglycerides | Other lipids |
| 23436 | -0.0102161 | 2.44E-01 | 5671 | Cholines | Other lipids |
| 23437 | -0.0065314 | 4.58E-01 | 5671 | Phosphatidylc | Other lipids |
| 23438 | -0.0173878 | 0.0423415 | 5671 | Sphingomyelins | Other lipids |
| 23439 | 0.00938389 | 0.3118574 | 5671 | ApoB | Apolipoproteins |
| 23440 | -0.0241547 | 5.27E-03 | 5671 | ApoA1 | Apolipoproteins |
| 23442 | 0.01291646 | 0.1789429 | 5671 | Total_FA | Fatty acids |
| 23443 | -0.044196 | 2.377E-06 | 5671 | Unsaturation | Fatty acids |
| 23444 | 7.82E-03 | 0.4040762 | 5671 | Omega_3 | Fatty acids |
| 23445 | -9.36E-04 | 0.9187305 | 5671 | Omega_6 | Fatty acids |
| 23446 | 7.63E-04 | 0.9336156 | 5671 | PUFA | Fatty acids |
| 23447 | 0.02991908 | 1.86E-03 | 5671 | MUFA | Fatty acids |
| 23448 | 0.01892818 | 0.0457064 | 5671 | SFA | Fatty acids |
| 23449 | 4.24E-03 | 0.6427898 | 5671 | LA | Fatty acids |
| 23450 | -7.63E-03 | 0.4048009 | 5671 | DHA | Fatty acids |
| 23460 | -3.18E-03 | 0.7409085 | 5671 | Alanine | Amino acids |
| 23461 | -0.0105223 | 0.2741417 | 5671 | Glutamine | Amino acids |
| 23462 | 1.04E-02 | 0.2665702 | 5671 | Glycine | Amino acids |
| 23463 | 5.48E-03 | 0.5471298 | 5671 | Histidine | Amino acids |
| 23464 | 4.48E-03 | 0.6371514 | 5671 | Total_BCAA | Amino acids |
| 23465 | 0.00653099 | 0.493033 | 5671 | Isoleucine | Amino acids |
| 23466 | 9.23E-04 | 0.9218563 | 5671 | Leucine | Amino acids |
| 23467 | 0.00479982 | 0.6122041 | 5671 | Valine | Amino acids |
| 23468 | 6.08E-03 | 0.5486938 | 5671 | Phenylalanine | Amino acids |
| 23469 | 1.47E-02 | 0.1203088 | 5671 | Tyrosine | Amino acids |
| 23470 | -7.87E-03 | 0.3175574 | 5671 | Glucose | Glycolysis related metabolites |
| 23471 | 4.03E-03 | 0.672441 | 5671 | Lactate | Glycolysis related metabolites |
| 23472 | 1.02E-02 | 0.2886804 | 5671 | Pyruvate | Glycolysis related metabolites |
| 23473 | -0.0201461 | 4.11E-02 | 5671 | Citrate | Glycolysis related metabolites |
| 23474 | -2.65E-02 | 0.0072018 | 5671 | bOHbutyrate | Ketone bodies |
| 23475 | 2.12E-02 | 0.0530975 | 5671 | Acetate | Ketone bodies |
| 23476 | -2.08E-02 | 0.0286818 | 5671 | Acetoacetate | Ketone bodies |
| 23477 | -2.73E-02 | 0.0058711 | 5671 | Acetone | Ketone bodies |
| 23478 | 3.32E-03 | 0.7479631 | 5671 | Creatinine | Fluid balance |
| 23479 | -0.0127605 | 0.1856052 | 5671 | Albumin | Fluid balance |
| 23480 | 0.0171667 | 6.53E-02 | 5671 | GlycA | Inflammation |
| 23481 | 0.04262388 | 4.87E-06 | 5671 | XXL_VLDL_P | Chylomicrons and Extremely Large VLDL |
| 23482 | 0.04336418 | 3.12E-06 | 5671 | XXL_VLDL_L | Chylomicrons and Extremely Large VLDL |
| 23483 | 0.04209245 | 6.11E-06 | 5671 | XXL_VLDL_PL | Chylomicrons and Extremely Large VLDL |
| 23484 | 0.0407565 | 1.42E-05 | 5671 | XXL_VLDL_C | Chylomicrons and Extremely Large VLDL |
| 23485 | 0.04016118 | 2.05E-05 | 5671 | XXL_VLDL_CE | Chylomicrons and Extremely Large VLDL |
| 23486 | 0.0406976 | 1.37E-05 | 5671 | XXL_VLDL_FC | Chylomicrons and Extremely Large VLDL |
| 23487 | 4.35E-02 | 2.859E-06 | 5671 | XXL_VLDL_TG | Chylomicrons and Extremely Large VLDL |
| 23488 | 0.04378555 | 3.42E-06 | 5671 | XL_VLDL_P | Very Large VLDL |
| 23489 | 0.04397399 | 2.93E-06 | 5671 | XL_VLDL_L | Very Large VLDL |
| 23490 | 0.0428318 | 5.687E-06 | 5671 | XL_VLDL_PL | Very Large VLDL |
| 23491 | 0.03875798 | 4.93E-05 | 5671 | XL_VLDL_C | Very Large VLDL |
| 23492 | 0.03446359 | 3.29E-04 | 5671 | XL_VLDL_CE | Very Large VLDL |
| 23493 | 0.04184143 | 1.03E-05 | 5671 | XL_VLDL_FC | Very Large VLDL |
| 23494 | 0.04469285 | 1.75E-06 | 5671 | XL_VLDL_TG | Very Large VLDL |
| 23495 | 0.04277178 | 7.21E-06 | 5671 | L_VLDL_P | Large VLDL |
| 23496 | 0.04278261 | 7.00E-06 | 5671 | L_VLDL_L | Large VLDL |
| 23497 | 0.04313918 | 5.679E-06 | 5671 | L_VLDL_PL | Large VLDL |
| 23498 | 0.03868834 | 5.81E-05 | 5671 | L_VLDL_C | Large VLDL |
| 23499 | 0.034422 | 3.84E-04 | 5671 | L_VLDL_CE | Large VLDL |
| 23500 | 0.04158647 | 1.25E-05 | 5671 | L_VLDL_FC | Large VLDL |
| 23501 | 0.04279624 | 6.70E-06 | 5671 | L_VLDL_TG | Large VLDL |
| 23502 | 0.02400017 | 1.23E-02 | 5671 | M_VLDL_P | Medium VLDL |
| 23503 | 0.03074302 | 1.39E-03 | 5671 | M_VLDL_L | Medium VLDL |
| 23504 | 0.02305213 | 1.56E-02 | 5671 | M_VLDL_PL | Medium VLDL |
| 23505 | 0.00538528 | 0.5584434 | 5671 | M_VLDL_C | Medium VLDL |
| 23506 | -4.09E-03 | 0.6497769 | 5671 | M_VLDL_CE | Medium VLDL |
| 23507 | 0.01757956 | 0.0628891 | 5671 | M_VLDL_FC | Medium VLDL |
| 23508 | 0.04111398 | 1.99E-05 | 5671 | M_VLDL_TG | Medium VLDL |
| 23509 | 0.03392177 | 4.97E-04 | 5671 | S_VLDL_P | Small VLDL |
| 23510 | 0.03250601 | 8.27E-04 | 5671 | S_VLDL_L | Small VLDL |
| 23511 | 0.02263051 | 1.87E-02 | 5671 | S_VLDL_PL | Small VLDL |
| 23512 | 0.01849252 | 5.36E-02 | 5671 | S_VLDL_C | Small VLDL |
| 23513 | 0.02085165 | 3.07E-02 | 5671 | S_VLDL_CE | Small VLDL |
| 23514 | 0.01392682 | 0.1411923 | 5671 | S_VLDL_FC | Small VLDL |
| 23515 | 0.04249287 | 1.12E-05 | 5671 | S_VLDL_TG | Small VLDL |
| 23516 | 0.0124708 | 0.1783543 | 5671 | XS_VLDL_P | Very Small VLDL |
| 23517 | 0.01308646 | 0.1545316 | 5671 | XS_VLDL_L | Very Small VLDL |
| 23518 | 0.01587908 | 0.0882573 | 5671 | XS_VLDL_PL | Very Small VLDL |
| 23519 | 8.69E-04 | 0.921811 | 5671 | XS_VLDL_C | Very Small VLDL |
| 23520 | -2.36E-03 | 0.7872233 | 5671 | XS_VLDL_CE | Very Small VLDL |
| 23521 | 0.00863616 | 0.345044 | 5671 | XS_VLDL_FC | Very Small VLDL |
| 23522 | 0.03658092 | 1.70E-04 | 5671 | XS_VLDL_TG | Very Small VLDL |
| 23523 | -1.72E-04 | 0.9847827 | 5671 | IDL_P | IDL |
| 23524 | -7.04E-03 | 0.4156202 | 5671 | IDL_L | IDL |
| 23525 | -6.96E-03 | 0.4204398 | 5671 | IDL_PL | IDL |
| 23526 | -0.010538 | 0.2203046 | 5671 | IDL_C | IDL |
| 23527 | -0.0092517 | 0.2819354 | 5671 | IDL_CE | IDL |
| 23528 | -0.0139104 | 0.1079335 | 5671 | IDL_FC | IDL |
| 23529 | 0.02984307 | 0.0021125 | 5671 | IDL_TG | IDL |
| 23530 | 0.00576097 | 0.5322946 | 5671 | L_LDL_P | Large LDL |
| 23531 | -3.28E-03 | 0.7183874 | 5671 | L_LDL_L | Large LDL |
| 23532 | -4.07E-03 | 0.6552568 | 5671 | L_LDL_PL | Large LDL |
| 23533 | -5.34E-03 | 0.5552152 | 5671 | L_LDL_C | Large LDL |
| 23534 | -2.53E-03 | 0.7820025 | 5671 | L_LDL_CE | Large LDL |
| 23535 | -1.28E-02 | 0.1489515 | 5671 | L_LDL_FC | Large LDL |
| 23536 | 0.0270514 | 0.0053596 | 5671 | L_LDL_TG | Large LDL |
| 23537 | 0.01026647 | 0.2800578 | 5671 | M_LDL_P | Medium LDL |
| 23538 | 0.00932978 | 0.3250156 | 5671 | M_LDL_L | Medium LDL |
| 23539 | 0.00896254 | 0.3448653 | 5671 | M_LDL_PL | Medium LDL |
| 23540 | 0.00727377 | 0.4414094 | 5671 | M_LDL_C | Medium LDL |
| 23541 | 0.01170305 | 0.2182488 | 5671 | M_LDL_CE | Medium LDL |
| 23542 | -5.12E-03 | 0.5808453 | 5671 | M_LDL_FC | Medium LDL |
| 23543 | 0.03296802 | 6.76E-04 | 5671 | M_LDL_TG | Medium LDL |
| 23544 | 0.01593196 | 0.0901697 | 5671 | S_LDL_P | Small LDL |
| 23545 | 0.01024334 | 0.274989 | 5671 | S_LDL_L | Small LDL |
| 23546 | 0.00694599 | 0.456977 | 5671 | S_LDL_PL | Small LDL |
| 23547 | 0.00733987 | 0.4328484 | 5671 | S_LDL_C | Small LDL |
| 23548 | 0.01218026 | 0.1955377 | 5671 | S_LDL_CE | Small LDL |
| 23549 | -5.99E-03 | 0.5183295 | 5671 | S_LDL_FC | Small LDL |
| 23550 | 0.04049785 | 2.56E-05 | 5671 | S_LDL_TG | Small LDL |
| 23551 | -0.0243162 | 3.95E-03 | 5671 | XL_HDL_P | Very Large HDL |
| 23552 | -0.0272865 | 1.15E-03 | 5671 | XL_HDL_L | Very Large HDL |
| 23553 | -0.0262429 | 1.99E-03 | 5671 | XL_HDL_PL | Very Large HDL |
| 23554 | -0.0293203 | 4.92E-04 | 5671 | XL_HDL_C | Very Large HDL |
| 23555 | -0.0301694 | 3.21E-04 | 5671 | XL_HDL_CE | Very Large HDL |
| 23556 | -0.0241081 | 0.0054366 | 5671 | XL_HDL_FC | Very Large HDL |
| 23557 | 2.40E-02 | 0.0127933 | 5671 | XL_HDL_TG | Very Large HDL |
| 23558 | -0.0313 | 1.92E-04 | 5671 | L_HDL_P | Large HDL |
| 23559 | -0.0337098 | 3.98E-05 | 5671 | L_HDL_L | Large HDL |
| 23560 | -0.0316302 | 1.51E-04 | 5671 | L_HDL_PL | Large HDL |
| 23561 | -0.0355379 | 1.72E-05 | 5671 | L_HDL_C | Large HDL |
| 23562 | -0.0362408 | 1.31E-05 | 5671 | L_HDL_CE | Large HDL |
| 23563 | -0.0305042 | 2.57E-04 | 5671 | L_HDL_FC | Large HDL |
| 23564 | 1.03E-02 | 0.2766002 | 5671 | L_HDL_TG | Large HDL |
| 23565 | -2.36E-02 | 0.0079469 | 5671 | M_HDL_P | Medium HDL |
| 23566 | -0.0208146 | 1.89E-02 | 5671 | M_HDL_L | Medium HDL |
| 23567 | -0.0157773 | 7.90E-02 | 5671 | M_HDL_PL | Medium HDL |
| 23568 | -0.0294655 | 8.10E-04 | 5671 | M_HDL_C | Medium HDL |
| 23569 | -0.0304692 | 5.79E-04 | 5671 | M_HDL_CE | Medium HDL |
| 23570 | -0.0240701 | 5.72E-03 | 5671 | M_HDL_FC | Medium HDL |
| 23571 | 0.0324505 | 0.0008947 | 5671 | M_HDL_TG | Medium HDL |
| 23572 | -0.0033779 | 0.7265492 | 5671 | S_HDL_P | Small HDL |
| 23573 | 0.00383898 | 0.6889922 | 5671 | S_HDL_L | Small HDL |
| 23574 | 0.00408255 | 0.6690881 | 5671 | S_HDL_PL | Small HDL |
| 23575 | -0.0079354 | 0.4108203 | 5671 | S_HDL_C | Small HDL |
| 23576 | -0.0096245 | 0.3201848 | 5671 | S_HDL_CE | Small HDL |
| 23577 | -0.0018945 | 0.841427 | 5671 | S_HDL_FC | Small HDL |
| 23578 | 0.04181614 | 1.04E-05 | 5671 | S_HDL_TG | Small HDL |
| 30000 | 0.0029915 | 7.39E-01 | 6555 | White blood cell (leukocyte) count | White blood cell |
| 30010 | -0.0077589 | 3.16E-01 | 6555 | Red blood cell (erythrocyte) count | Red blood cell |
| 30020 | -1.01E-02 | 0.1641019 | 6556 | Haemoglobin concentration | Red blood cell |
| 30030 | -1.28E-02 | 0.0852121 | 6556 | Haematocrit percentage | Red blood cell |
| 30040 | -0.0079811 | 3.54E-01 | 6556 | Mean corpuscular volume | Red blood cell |
| 30050 | -0.0037492 | 6.55E-01 | 6556 | Mean corpuscular haemoglobin | Red blood cell |
| 30060 | 4.65E-03 | 0.5771232 | 6556 | Mean corpuscular haemoglobin concentration | Red blood cell |
| 30070 | 1.07E-02 | 0.2264944 | 6556 | Red blood cell (erythrocyte) distribution width | Red blood cell |
| 30080 | 6.87E-03 | 0.4257664 | 6556 | Platelet count | Platelet |
| 30090 | -6.02E-03 | 0.5419529 | 4757 | Platelet crit | Platelet |
| 30100 | -7.86E-03 | 0.3885196 | 6555 | Mean platelet (thrombocyte) volume | Platelet |
| 30110 | 6.06E-03 | 0.5662709 | 4757 | Platelet distribution width | Platelet |
| 30120 | -0.0005544 | 0.9506916 | 6550 | Lymphocyte count | White blood cell |
| 30130 | 0.02463426 | 6.04E-03 | 6550 | Monocyte count | White blood cell |
| 30140 | -0.0031483 | 7.25E-01 | 6550 | Neutrophill count | White blood cell |
| 30150 | 0.02671086 | 0.0021441 | 6550 | Eosinophill count | White blood cell |
| 30160 | 4.27E-04 | 0.9625672 | 6550 | Basophill count | White blood cell |
| 30170 | -5.18E-03 | 0.5930694 | 4747 | Nucleated red blood cell count | Red blood cell |
| 30180 | -0.0026302 | 0.768225 | 6551 | Lymphocyte percentage | White blood cell |
| 30190 | 2.57E-02 | 0.0050161 | 6551 | Monocyte percentage | White blood cell |
| 30200 | -1.13E-02 | 0.2139229 | 6551 | Neutrophill percentage | White blood cell |
| 30210 | 2.97E-02 | 0.000979 | 6551 | Eosinophill percentage | White blood cell |
| 30220 | 2.05E-03 | 0.821982 | 6551 | Basophill percentage | White blood cell |
| 30230 | -7.57E-03 | 0.440103 | 4747 | Nucleated red blood cell percentage | Red blood cell |
| 30240 | 1.56E-02 | 0.0763266 | 6529 | Reticulocyte percentage | Red blood cell |
| 30250 | 0.01664395 | 0.0536391 | 6529 | Reticulocyte count | Red blood cell |
| 30260 | -0.0030916 | 0.7302065 | 6529 | Mean reticulocyte volume | Red blood cell |
| 30270 | -0.0143876 | 1.64E-01 | 4755 | Mean sphered cell volume | Red blood cell |
| 30280 | 0.01639477 | 1.08E-01 | 4755 | Immature reticulocyte fraction | Red blood cell |
| 30290 | 0.02685112 | 7.47E-03 | 4755 | High light scatter reticulocyte percentage | Red blood cell |
| 30300 | 0.02986776 | 2.86E-03 | 4755 | High light scatter reticulocyte count | Red blood cell |
| 30600 | -1.38E-02 | 0.1594016 | 5236 | Albumin | Liver function |
| 30610 | 0.02178217 | 0.0196134 | 5995 | Alkaline phosphatase | Bone and joint |
| 30620 | 0.00326334 | 0.7145108 | 5995 | Alanine aminotransferase | Liver function |
| 30630 | -0.0247823 | 5.11E-03 | 5190 | Apolipoprotein A | Immunometabolism |
| 30640 | -0.0054626 | 5.55E-01 | 5973 | Apolipoprotein B | Immunometabolism |
| 30650 | -0.0180531 | 6.18E-02 | 5972 | Aspartate aminotransferase | Liver function |
| 30660 | -0.0091132 | 0.3609975 | 5065 | Direct bilirubin | Liver function |
| 30670 | -0.0176501 | 4.95E-02 | 5990 | Urea | Renal function |
| 30680 | -1.53E-03 | 0.8810984 | 5236 | Calcium | Bone and joint |
| 30690 | -6.52E-03 | 0.4492616 | 5998 | Cholesterol | Immunometabolism |
| 30700 | 9.22E-03 | 0.2113255 | 5987 | Creatinine | Renal function |
| 30710 | 0.01637812 | 0.0641502 | 5986 | C-reactive protein | Immunometabolism |
| 30720 | 0.02008304 | 1.20E-02 | 5988 | Cystatin C | Renal function |
| 30730 | 1.07E-02 | 0.2267279 | 5995 | Gamma glutamyltransferase | Liver function |
| 30740 | -6.41E-03 | 0.3881698 | 5235 | Glucose | Endocrine |
| 30750 | 0.02553715 | 0.0010094 | 4689 | Glycated haemoglobin (HbA1c) | Immunometabolism |
| 30760 | -0.0336106 | 6.79E-05 | 5235 | HDL cholesterol | Immunometabolism |
| 30770 | -3.11E-02 | 0.0008012 | 5936 | IGF-1 | Endocrine |
| 30780 | -0.0025929 | 0.7715925 | 5991 | LDL direct | Immunometabolism |
| 30790 | -1.59E-02 | 0.1471264 | 4782 | Lipoprotein A | Endocrine |
| 30800 | 0.01106023 | 0.6895115 | 659 | Oestradiol | Endocrine |
| 30810 | 6.81E-03 | 0.4838768 | 5220 | Phosphate | Renal function |
| 30820 | 0.02506577 | 0.436732 | 576 | Rheumatoid factor | Immunometabolism |
| 30830 | -0.0362538 | 4.20E-05 | 5151 | SHBG | Endocrine |
| 30840 | -0.0101008 | 0.2740988 | 5963 | Total bilirubin | Liver function |
| 30850 | -0.0103972 | 1.98E-02 | 5369 | Testosterone | Endocrine |
| 30860 | -1.72E-02 | 0.0860209 | 5234 | Total protein | Immunometabolism |
| 30870 | 0.04523978 | 8.90E-07 | 5987 | Triglycerides | Immunometabolism |
| 30880 | 5.73E-03 | 0.4542827 | 5985 | Urate | Renal function |
| 30890 | -0.0350651 | 2.34E-04 | 5726 | Vitamin D | Bone and joint |

| **Table S12. Associations of repeated measurements Blood and Metabolomic Biomarkers with pseudo-F.** | | | | | |
| --- | --- | --- | --- | --- | --- |
| **Field ID** | **Beta** | **P Value** | **Sample size** | **Biomarker** | **Group** |
| 20280 | -6.5E-05 | 0.2736678 | 5671 | Glucose-lactate | Glycolysis related metabolites |
| 20281 | 2.50E-05 | 0.7197986 | 5671 | Spectrometer-corrected alanine | Amino acids |
| 23400 | 9.8581E-05 | 0.1261642 | 5671 | Total_C | Cholesterol |
| 23401 | 7.21E-05 | 0.2865853 | 5671 | Non_HDL_C | Cholesterol |
| 23402 | 4.54E-05 | 0.5016069 | 5671 | Remnant_C | Cholesterol |
| 23403 | 1.7774E-05 | 8.00E-01 | 5671 | VLDL Cholesterol | Cholesterol |
| 23404 | 7.93E-05 | 0.2399768 | 5671 | Clinical_LDL_C | Cholesterol |
| 23405 | 9.49E-05 | 0.163276 | 5671 | LDL_C | Cholesterol |
| 23406 | 0.00010929 | 7.75E-02 | 5671 | HDL_C | Cholesterol |
| 23407 | 9.7279E-06 | 8.91E-01 | 5671 | Total_TG | Triglycerides |
| 23408 | 9.421E-06 | 8.93E-01 | 5671 | VLDL_TG | Triglycerides |
| 23409 | 2.3697E-05 | 7.42E-01 | 5671 | LDL_TG | Triglycerides |
| 23410 | 1.2167E-05 | 0.8668492 | 5671 | HDL_TG | Triglycerides |
| 23411 | 0.00011195 | 0.0842898 | 5671 | Total_PL | Phospholipids |
| 23412 | 1.2719E-05 | 8.58E-01 | 5671 | VLDL_PL | Phospholipids |
| 23413 | 8.21E-05 | 0.2307888 | 5671 | LDL_PL | Phospholipids |
| 23414 | 0.00011057 | 8.10E-02 | 5671 | HDL_PL | Phospholipids |
| 23415 | 0.000104 | 0.1052033 | 5671 | Total_CE | Cholesteryl Esters |
| 23416 | 1.8975E-05 | 0.7851054 | 5671 | VLDL_CE | Cholesteryl Esters |
| 23417 | 9.64E-05 | 0.1604085 | 5671 | LDL_CE | Cholesteryl Esters |
| 23418 | 0.0001099 | 7.85E-02 | 5671 | HDL_CE | Cholesteryl Esters |
| 23419 | 8.30E-05 | 0.2044723 | 5671 | Total_FC | Free Cholesterol |
| 23420 | 2.1441E-05 | 7.63E-01 | 5671 | VLDL_FC | Free Cholesterol |
| 23421 | 8.78E-05 | 0.1883592 | 5671 | LDL_FC | Free Cholesterol |
| 23422 | 9.9605E-05 | 1.07E-01 | 5671 | HDL_FC | Free Cholesterol |
| 23423 | 9.92E-05 | 0.1429379 | 5671 | Total_L | Total Lipids |
| 23424 | 4.55E-06 | 9.49E-01 | 5671 | VLDL_L | Total Lipids |
| 23425 | 9.06E-05 | 0.185938 | 5671 | LDL_L | Total Lipids |
| 23426 | 0.00011156 | 7.35E-02 | 5671 | HDL_L | Total Lipids |
| 23427 | 0.00014451 | 2.87E-02 | 5671 | Total_P | Particle Concentrations |
| 23428 | 2.7737E-05 | 6.98E-01 | 5671 | VLDL_P | Particle Concentrations |
| 23429 | 6.0429E-05 | 0.380048 | 5671 | LDL_P | Particle Concentrations |
| 23430 | 0.00014403 | 3.04E-02 | 5671 | HDL_P | Particle Concentrations |
| 23431 | -1.942E-05 | 7.71E-01 | 5671 | VLDL_size | Lipoprotein particle size |
| 23432 | 3.6319E-05 | 0.5975838 | 5671 | LDL_size | Lipoprotein particle size |
| 23433 | 2.1764E-05 | 7.24E-01 | 5671 | HDL_size | Lipoprotein particle size |
| 23434 | 0.00011049 | 9.42E-02 | 5671 | Phosphoglycerides | Other lipids |
| 23436 | 0.00011132 | 8.65E-02 | 5671 | Cholines | Other lipids |
| 23437 | 0.00011043 | 9.02E-02 | 5671 | Phosphatidylc | Other lipids |
| 23438 | 6.7486E-05 | 0.2871646 | 5671 | Sphingomyelins | Other lipids |
| 23439 | 5.0732E-05 | 0.4601263 | 5671 | ApoB | Apolipoproteins |
| 23440 | 0.00012255 | 5.59E-02 | 5671 | ApoA1 | Apolipoproteins |
| 23442 | 6.7237E-05 | 0.3445843 | 5671 | Total_FA | Fatty acids |
| 23443 | 6.171E-05 | 0.3738922 | 5671 | Unsaturation | Fatty acids |
| 23444 | 2.00E-05 | 0.7735206 | 5671 | Omega_3 | Fatty acids |
| 23445 | 1.24E-04 | 0.0682039 | 5671 | Omega_6 | Fatty acids |
| 23446 | 1.10E-04 | 0.1043679 | 5671 | PUFA | Fatty acids |
| 23447 | 2.7978E-05 | 6.94E-01 | 5671 | MUFA | Fatty acids |
| 23448 | 5.8513E-05 | 0.4040862 | 5671 | SFA | Fatty acids |
| 23449 | 1.19E-04 | 0.0794848 | 5671 | LA | Fatty acids |
| 23450 | 3.74E-06 | 0.9560096 | 5671 | DHA | Fatty acids |
| 23460 | -1.10E-05 | 0.8771883 | 5671 | Alanine | Amino acids |
| 23461 | 3.2234E-05 | 0.6508567 | 5671 | Glutamine | Amino acids |
| 23462 | -1.68E-05 | 0.8089591 | 5671 | Glycine | Amino acids |
| 23463 | 2.92E-07 | 0.9965458 | 5671 | Histidine | Amino acids |
| 23464 | 3.55E-05 | 0.613828 | 5671 | Total_BCAA | Amino acids |
| 23465 | 2.8855E-05 | 0.6824225 | 5671 | Isoleucine | Amino acids |
| 23466 | 2.47E-05 | 0.7227789 | 5671 | Leucine | Amino acids |
| 23467 | 4.3415E-05 | 0.5356129 | 5671 | Valine | Amino acids |
| 23468 | 5.94E-05 | 0.4284601 | 5671 | Phenylalanine | Amino acids |
| 23469 | -5.27E-05 | 0.4512473 | 5671 | Tyrosine | Amino acids |
| 23470 | -3.26E-05 | 0.5762355 | 5671 | Glucose | Glycolysis related metabolites |
| 23471 | -8.93E-05 | 0.2054478 | 5671 | Lactate | Glycolysis related metabolites |
| 23472 | -9.60E-05 | 0.1774564 | 5671 | Pyruvate | Glycolysis related metabolites |
| 23473 | 6.3381E-05 | 3.85E-01 | 5671 | Citrate | Glycolysis related metabolites |
| 23474 | 5.52E-05 | 0.4503865 | 5671 | bOHbutyrate | Ketone bodies |
| 23475 | 1.26E-05 | 0.8765352 | 5671 | Acetate | Ketone bodies |
| 23476 | 8.07E-05 | 0.2505261 | 5671 | Acetoacetate | Ketone bodies |
| 23477 | 6.22E-05 | 0.3960701 | 5671 | Acetone | Ketone bodies |
| 23478 | 7.03E-05 | 0.3584986 | 5671 | Creatinine | Fluid balance |
| 23479 | 1.5708E-05 | 0.8257728 | 5671 | Albumin | Fluid balance |
| 23480 | -2.397E-05 | 7.28E-01 | 5671 | GlycA | Inflammation |
| 23481 | -6.612E-06 | 9.24E-01 | 5671 | XXL_VLDL_P | Chylomicrons and Extremely Large VLDL |
| 23482 | -1.88E-05 | 7.85E-01 | 5671 | XXL_VLDL_L | Chylomicrons and Extremely Large VLDL |
| 23483 | -1.727E-05 | 8.02E-01 | 5671 | XXL_VLDL_PL | Chylomicrons and Extremely Large VLDL |
| 23484 | -7.41E-06 | 9.15E-01 | 5671 | XXL_VLDL_C | Chylomicrons and Extremely Large VLDL |
| 23485 | -2.856E-07 | 9.97E-01 | 5671 | XXL_VLDL_CE | Chylomicrons and Extremely Large VLDL |
| 23486 | -1.29E-05 | 8.52E-01 | 5671 | XXL_VLDL_FC | Chylomicrons and Extremely Large VLDL |
| 23487 | -1.39E-05 | 0.8402546 | 5671 | XXL_VLDL_TG | Chylomicrons and Extremely Large VLDL |
| 23488 | 1.566E-05 | 8.23E-01 | 5671 | XL_VLDL_P | Very Large VLDL |
| 23489 | 9.436E-06 | 8.92E-01 | 5671 | XL_VLDL_L | Very Large VLDL |
| 23490 | 6.3405E-06 | 0.9277578 | 5671 | XL_VLDL_PL | Very Large VLDL |
| 23491 | 1.3608E-05 | 8.47E-01 | 5671 | XL_VLDL_C | Very Large VLDL |
| 23492 | 2.0498E-05 | 7.73E-01 | 5671 | XL_VLDL_CE | Very Large VLDL |
| 23493 | 7.8366E-06 | 9.11E-01 | 5671 | XL_VLDL_FC | Very Large VLDL |
| 23494 | 1.5735E-05 | 8.20E-01 | 5671 | XL_VLDL_TG | Very Large VLDL |
| 23495 | 2.8013E-05 | 6.92E-01 | 5671 | L_VLDL_P | Large VLDL |
| 23496 | 2.1828E-05 | 7.57E-01 | 5671 | L_VLDL_L | Large VLDL |
| 23497 | 1.5738E-05 | 0.8231831 | 5671 | L_VLDL_PL | Large VLDL |
| 23498 | 1.9447E-05 | 7.85E-01 | 5671 | L_VLDL_C | Large VLDL |
| 23499 | 1.8371E-05 | 7.98E-01 | 5671 | L_VLDL_CE | Large VLDL |
| 23500 | 2.2873E-05 | 7.46E-01 | 5671 | L_VLDL_FC | Large VLDL |
| 23501 | 3.6551E-05 | 6.04E-01 | 5671 | L_VLDL_TG | Large VLDL |
| 23502 | 5.1636E-05 | 4.67E-01 | 5671 | M_VLDL_P | Medium VLDL |
| 23503 | 4.3632E-05 | 5.40E-01 | 5671 | M_VLDL_L | Medium VLDL |
| 23504 | 4.2956E-05 | 5.43E-01 | 5671 | M_VLDL_PL | Medium VLDL |
| 23505 | 4.1687E-05 | 0.5405745 | 5671 | M_VLDL_C | Medium VLDL |
| 23506 | 3.58E-05 | 0.5916616 | 5671 | M_VLDL_CE | Medium VLDL |
| 23507 | 4.5658E-05 | 0.5140525 | 5671 | M_VLDL_FC | Medium VLDL |
| 23508 | 4.493E-05 | 5.29E-01 | 5671 | M_VLDL_TG | Medium VLDL |
| 23509 | 2.5908E-05 | 7.20E-01 | 5671 | S_VLDL_P | Small VLDL |
| 23510 | 2.477E-05 | 7.31E-01 | 5671 | S_VLDL_L | Small VLDL |
| 23511 | 3.5926E-05 | 6.14E-01 | 5671 | S_VLDL_PL | Small VLDL |
| 23512 | 1.5665E-05 | 8.25E-01 | 5671 | S_VLDL_C | Small VLDL |
| 23513 | 2.4867E-06 | 9.72E-01 | 5671 | S_VLDL_CE | Small VLDL |
| 23514 | 3.8535E-05 | 0.5823391 | 5671 | S_VLDL_FC | Small VLDL |
| 23515 | 2.8175E-05 | 6.94E-01 | 5671 | S_VLDL_TG | Small VLDL |
| 23516 | 4.3529E-06 | 0.9494009 | 5671 | XS_VLDL_P | Very Small VLDL |
| 23517 | 7.5544E-06 | 0.9115989 | 5671 | XS_VLDL_L | Very Small VLDL |
| 23518 | -3.809E-06 | 0.9559551 | 5671 | XS_VLDL_PL | Very Small VLDL |
| 23519 | 1.42E-05 | 0.8289004 | 5671 | XS_VLDL_C | Very Small VLDL |
| 23520 | 1.77E-05 | 0.7838659 | 5671 | XS_VLDL_CE | Very Small VLDL |
| 23521 | 4.8928E-06 | 0.9423869 | 5671 | XS_VLDL_FC | Very Small VLDL |
| 23522 | 3.4917E-06 | 9.61E-01 | 5671 | XS_VLDL_TG | Very Small VLDL |
| 23523 | 1.91E-05 | 0.7744391 | 5671 | IDL_P | IDL |
| 23524 | 5.95E-05 | 0.3526473 | 5671 | IDL_L | IDL |
| 23525 | 5.95E-05 | 0.3519969 | 5671 | IDL_PL | IDL |
| 23526 | 6.0553E-05 | 0.3413524 | 5671 | IDL_C | IDL |
| 23527 | 6.5316E-05 | 0.3047787 | 5671 | IDL_CE | IDL |
| 23528 | 4.5779E-05 | 0.4748135 | 5671 | IDL_FC | IDL |
| 23529 | 9.3175E-06 | 0.8968793 | 5671 | IDL_TG | IDL |
| 23530 | 5.8366E-05 | 0.3926714 | 5671 | L_LDL_P | Large LDL |
| 23531 | 9.39E-05 | 0.1629133 | 5671 | L_LDL_L | Large LDL |
| 23532 | 8.26E-05 | 0.2203414 | 5671 | L_LDL_PL | Large LDL |
| 23533 | 9.88E-05 | 0.1400515 | 5671 | L_LDL_C | Large LDL |
| 23534 | 1.01E-04 | 0.133382 | 5671 | L_LDL_CE | Large LDL |
| 23535 | 8.93E-05 | 0.1733659 | 5671 | L_LDL_FC | Large LDL |
| 23536 | 2.3441E-05 | 0.7445171 | 5671 | L_LDL_TG | Large LDL |
| 23537 | 6.7733E-05 | 0.335659 | 5671 | M_LDL_P | Medium LDL |
| 23538 | 8.4883E-05 | 0.2263779 | 5671 | M_LDL_L | Medium LDL |
| 23539 | 8.3118E-05 | 0.2366107 | 5671 | M_LDL_PL | Medium LDL |
| 23540 | 8.6904E-05 | 0.2140099 | 5671 | M_LDL_C | Medium LDL |
| 23541 | 8.4967E-05 | 0.2271965 | 5671 | M_LDL_CE | Medium LDL |
| 23542 | 8.61E-05 | 0.2099466 | 5671 | M_LDL_FC | Medium LDL |
| 23543 | 2.9432E-05 | 6.82E-01 | 5671 | M_LDL_TG | Medium LDL |
| 23544 | 4.3717E-05 | 0.5299416 | 5671 | S_LDL_P | Small LDL |
| 23545 | 7.2035E-05 | 0.2996748 | 5671 | S_LDL_L | Small LDL |
| 23546 | 6.5681E-05 | 0.3419815 | 5671 | S_LDL_PL | Small LDL |
| 23547 | 7.5551E-05 | 0.2753986 | 5671 | S_LDL_C | Small LDL |
| 23548 | 7.3672E-05 | 0.2902155 | 5671 | S_LDL_CE | Small LDL |
| 23549 | 7.33E-05 | 0.2856237 | 5671 | S_LDL_FC | Small LDL |
| 23550 | 2.0941E-05 | 7.69E-01 | 5671 | S_LDL_TG | Small LDL |
| 23551 | 2.2137E-05 | 7.23E-01 | 5671 | XL_HDL_P | Very Large HDL |
| 23552 | 1.7174E-05 | 7.82E-01 | 5671 | XL_HDL_L | Very Large HDL |
| 23553 | 9.5716E-06 | 8.79E-01 | 5671 | XL_HDL_PL | Very Large HDL |
| 23554 | 2.3357E-05 | 7.08E-01 | 5671 | XL_HDL_C | Very Large HDL |
| 23555 | 3.0628E-05 | 6.22E-01 | 5671 | XL_HDL_CE | Very Large HDL |
| 23556 | -6.892E-06 | 0.9145305 | 5671 | XL_HDL_FC | Very Large HDL |
| 23557 | 1.06E-05 | 0.8822331 | 5671 | XL_HDL_TG | Very Large HDL |
| 23558 | 5.565E-05 | 3.71E-01 | 5671 | L_HDL_P | Large HDL |
| 23559 | 6.2835E-05 | 3.01E-01 | 5671 | L_HDL_L | Large HDL |
| 23560 | 6.5529E-05 | 2.89E-01 | 5671 | L_HDL_PL | Large HDL |
| 23561 | 5.7283E-05 | 3.50E-01 | 5671 | L_HDL_C | Large HDL |
| 23562 | 5.6918E-05 | 3.55E-01 | 5671 | L_HDL_CE | Large HDL |
| 23563 | 5.2543E-05 | 3.95E-01 | 5671 | L_HDL_FC | Large HDL |
| 23564 | 1.25E-05 | 0.8584761 | 5671 | L_HDL_TG | Large HDL |
| 23565 | 1.24E-04 | 0.0596868 | 5671 | M_HDL_P | Medium HDL |
| 23566 | 0.00012788 | 5.14E-02 | 5671 | M_HDL_L | Medium HDL |
| 23567 | 0.0001264 | 5.73E-02 | 5671 | M_HDL_PL | Medium HDL |
| 23568 | 0.00012795 | 4.95E-02 | 5671 | M_HDL_C | Medium HDL |
| 23569 | 0.00012758 | 5.17E-02 | 5671 | M_HDL_CE | Medium HDL |
| 23570 | 0.00012464 | 5.32E-02 | 5671 | M_HDL_FC | Medium HDL |
| 23571 | 1.9995E-05 | 0.7822589 | 5671 | M_HDL_TG | Medium HDL |
| 23572 | 0.00014689 | 0.0398917 | 5671 | S_HDL_P | Small HDL |
| 23573 | 0.00014097 | 0.0470486 | 5671 | S_HDL_L | Small HDL |
| 23574 | 0.00013587 | 0.0546054 | 5671 | S_HDL_PL | Small HDL |
| 23575 | 0.00015266 | 0.0325229 | 5671 | S_HDL_C | Small HDL |
| 23576 | 0.00014825 | 0.038557 | 5671 | S_HDL_CE | Small HDL |
| 23577 | 0.00014451 | 0.03919 | 5671 | S_HDL_FC | Small HDL |
| 23578 | 4.2164E-06 | 9.52E-01 | 5671 | S_HDL_TG | Small HDL |
| 30000 | -2.039E-05 | 7.57E-01 | 6555 | White blood cell (leukocyte) count | White blood cell |
| 30010 | -3.196E-05 | 5.75E-01 | 6555 | Red blood cell (erythrocyte) count | Red blood cell |
| 30020 | 1.62E-05 | 0.7622778 | 6556 | Haemoglobin concentration | Red blood cell |
| 30030 | 1.91E-05 | 0.7262204 | 6556 | Haematocrit percentage | Red blood cell |
| 30040 | 9.2645E-05 | 1.43E-01 | 6556 | Mean corpuscular volume | Red blood cell |
| 30050 | 6.5729E-05 | 2.88E-01 | 6556 | Mean corpuscular haemoglobin | Red blood cell |
| 30060 | -1.09E-05 | 0.859636 | 6556 | Mean corpuscular haemoglobin concentration | Red blood cell |
| 30070 | 4.51E-05 | 0.4873707 | 6556 | Red blood cell (erythrocyte) distribution width | Red blood cell |
| 30080 | -2.25E-06 | 0.9716848 | 6556 | Platelet count | Platelet |
| 30090 | -1.76E-05 | 0.8002314 | 4757 | Platelet crit | Platelet |
| 30100 | -9.23E-05 | 0.1683458 | 6555 | Mean platelet (thrombocyte) volume | Platelet |
| 30110 | 4.36E-05 | 0.5578815 | 4757 | Platelet distribution width | Platelet |
| 30120 | -1.968E-05 | 0.7651911 | 6550 | Lymphocyte count | White blood cell |
| 30130 | 2.4984E-05 | 7.05E-01 | 6550 | Monocyte count | White blood cell |
| 30140 | -2.402E-05 | 7.15E-01 | 6550 | Neutrophill count | White blood cell |
| 30150 | -8.216E-05 | 0.1991195 | 6550 | Eosinophill count | White blood cell |
| 30160 | 3.03E-05 | 0.6502905 | 6550 | Basophill count | White blood cell |
| 30170 | -2.50E-05 | 0.7135827 | 4747 | Nucleated red blood cell count | Red blood cell |
| 30180 | -2.089E-06 | 0.9746044 | 6551 | Lymphocyte percentage | White blood cell |
| 30190 | 1.12E-05 | 0.8677894 | 6551 | Monocyte percentage | White blood cell |
| 30200 | -2.79E-05 | 0.6763489 | 6551 | Neutrophill percentage | White blood cell |
| 30210 | -5.33E-05 | 0.4217705 | 6551 | Eosinophill percentage | White blood cell |
| 30220 | 9.63E-05 | 0.1513863 | 6551 | Basophill percentage | White blood cell |
| 30230 | -2.78E-05 | 0.6870838 | 4747 | Nucleated red blood cell percentage | Red blood cell |
| 30240 | 4.30E-05 | 0.5061481 | 6529 | Reticulocyte percentage | Red blood cell |
| 30250 | 3.9113E-05 | 0.537492 | 6529 | Reticulocyte count | Red blood cell |
| 30260 | 8.3436E-05 | 0.205618 | 6529 | Mean reticulocyte volume | Red blood cell |
| 30270 | 7.8861E-06 | 9.14E-01 | 4755 | Mean sphered cell volume | Red blood cell |
| 30280 | -0.0001065 | 1.37E-01 | 4755 | Immature reticulocyte fraction | Red blood cell |
| 30290 | -5.545E-06 | 9.37E-01 | 4755 | High light scatter reticulocyte percentage | Red blood cell |
| 30300 | -1.529E-06 | 9.83E-01 | 4755 | High light scatter reticulocyte count | Red blood cell |
| 30600 | 4.95E-05 | 0.4719575 | 5236 | Albumin | Liver function |
| 30610 | -0.0001647 | 0.0138375 | 5995 | Alkaline phosphatase | Bone and joint |
| 30620 | 9.7189E-05 | 0.1285553 | 5995 | Alanine aminotransferase | Liver function |
| 30630 | 0.00010176 | 1.01E-01 | 5190 | Apolipoprotein A | Immunometabolism |
| 30640 | 5.6709E-05 | 3.93E-01 | 5973 | Apolipoprotein B | Immunometabolism |
| 30650 | 0.00018209 | 8.51E-03 | 5972 | Aspartate aminotransferase | Liver function |
| 30660 | 0.00018955 | 0.0066854 | 5065 | Direct bilirubin | Liver function |
| 30670 | 3.2599E-05 | 6.13E-01 | 5990 | Urea | Renal function |
| 30680 | 2.54E-05 | 0.7229341 | 5236 | Calcium | Bone and joint |
| 30690 | 6.31E-05 | 0.3075431 | 5998 | Cholesterol | Immunometabolism |
| 30700 | -1.24E-05 | 0.814131 | 5987 | Creatinine | Renal function |
| 30710 | -9.862E-05 | 0.1197541 | 5986 | C-reactive protein | Immunometabolism |
| 30720 | -7.55E-05 | 1.88E-01 | 5988 | Cystatin C | Renal function |
| 30730 | 6.31E-05 | 0.3183703 | 5995 | Gamma glutamyltransferase | Liver function |
| 30740 | -2.79E-05 | 0.5918486 | 5235 | Glucose | Endocrine |
| 30750 | -4.859E-05 | 0.36427 | 4689 | Glycated haemoglobin (HbA1c) | Immunometabolism |
| 30760 | 0.00010478 | 7.62E-02 | 5235 | HDL cholesterol | Immunometabolism |
| 30770 | 1.18E-04 | 0.0743786 | 5936 | IGF-1 | Endocrine |
| 30780 | 3.3831E-05 | 0.5969618 | 5991 | LDL direct | Immunometabolism |
| 30790 | -1.12E-04 | 0.1376669 | 4782 | Lipoprotein A | Endocrine |
| 30800 | 0.00040812 | 0.0756447 | 659 | Oestradiol | Endocrine |
| 30810 | -3.06E-05 | 0.6538463 | 5220 | Phosphate | Renal function |
| 30820 | -0.0001543 | 0.4713158 | 576 | Rheumatoid factor | Immunometabolism |
| 30830 | 0.00013395 | 3.03E-02 | 5151 | SHBG | Endocrine |
| 30840 | 0.00020576 | 0.0018451 | 5963 | Total bilirubin | Liver function |
| 30850 | 2.0132E-06 | 9.49E-01 | 5369 | Testosterone | Endocrine |
| 30860 | 3.96E-05 | 0.5735898 | 5234 | Total protein | Immunometabolism |
| 30870 | -7.787E-05 | 2.38E-01 | 5987 | Triglycerides | Immunometabolism |
| 30880 | -3.17E-05 | 0.5622408 | 5985 | Urate | Renal function |
| 30890 | 0.00015387 | 2.29E-02 | 5726 | Vitamin D | Bone and joint |

| **Table S13. Associations of repeated measurements Blood and Metabolomic Biomarkers with mesor.** | | | | | |
| --- | --- | --- | --- | --- | --- |
| **Field ID** | **Beta** | **P Value** | **Sample size** | **Biomarker** | **Group** |
| 20280 | -0.0004963 | 0.124527 | 5671 | Glucose-lactate | Glycolysis related metabolites |
| 20281 | -7.53E-05 | 0.8427123 | 5671 | Spectrometer-corrected alanine | Amino acids |
| 23400 | 0.00079285 | 0.0237851 | 5671 | Total_C | Cholesterol |
| 23401 | 1.94E-04 | 0.5981233 | 5671 | Non_HDL_C | Cholesterol |
| 23402 | -2.89E-05 | 0.9371992 | 5671 | Remnant_C | Cholesterol |
| 23403 | -0.0006269 | 1.01E-01 | 5671 | VLDL Cholesterol | Cholesterol |
| 23404 | 3.78E-04 | 0.303067 | 5671 | Clinical_LDL_C | Cholesterol |
| 23405 | 3.98E-04 | 0.2833735 | 5671 | LDL_C | Cholesterol |
| 23406 | 0.00183133 | 5.28E-08 | 5671 | HDL_C | Cholesterol |
| 23407 | -0.0010006 | 9.27E-03 | 5671 | Total_TG | Triglycerides |
| 23408 | -0.0010146 | 7.81E-03 | 5671 | VLDL_TG | Triglycerides |
| 23409 | -0.0008309 | 3.39E-02 | 5671 | LDL_TG | Triglycerides |
| 23410 | -0.0005431 | 0.1690233 | 5671 | HDL_TG | Triglycerides |
| 23411 | 0.00087198 | 0.0134617 | 5671 | Total_PL | Phospholipids |
| 23412 | -0.0009102 | 1.88E-02 | 5671 | VLDL_PL | Phospholipids |
| 23413 | 2.62E-04 | 0.481928 | 5671 | LDL_PL | Phospholipids |
| 23414 | 0.00162667 | 2.34E-06 | 5671 | HDL_PL | Phospholipids |
| 23415 | 0.00089955 | 0.0100052 | 5671 | Total_CE | Cholesteryl Esters |
| 23416 | -0.0004874 | 0.1980406 | 5671 | VLDL_CE | Cholesteryl Esters |
| 23417 | 3.16E-04 | 0.3980739 | 5671 | LDL_CE | Cholesteryl Esters |
| 23418 | 0.00187855 | 3.15E-08 | 5671 | HDL_CE | Cholesteryl Esters |
| 23419 | 5.06E-04 | 0.1546431 | 5671 | Total_FC | Free Cholesterol |
| 23420 | -0.0007954 | 3.97E-02 | 5671 | VLDL_FC | Free Cholesterol |
| 23421 | 6.01E-04 | 0.0978056 | 5671 | LDL_FC | Free Cholesterol |
| 23422 | 0.00158903 | 2.22E-06 | 5671 | HDL_FC | Free Cholesterol |
| 23423 | 4.02E-04 | 0.2749385 | 5671 | Total_L | Total Lipids |
| 23424 | -0.0009561 | 1.32E-02 | 5671 | VLDL_L | Total Lipids |
| 23425 | 3.03E-04 | 0.4158544 | 5671 | LDL_L | Total Lipids |
| 23426 | 0.00171043 | 4.50E-07 | 5671 | HDL_L | Total Lipids |
| 23427 | 0.00161768 | 6.66E-06 | 5671 | Total_P | Particle Concentrations |
| 23428 | -0.0007291 | 6.10E-02 | 5671 | VLDL_P | Particle Concentrations |
| 23429 | -0.0001047 | 0.779958 | 5671 | LDL_P | Particle Concentrations |
| 23430 | 0.00172263 | 1.92E-06 | 5671 | HDL_P | Particle Concentrations |
| 23431 | -0.0010276 | 4.56E-03 | 5671 | VLDL_size | Lipoprotein particle size |
| 23432 | 0.00068398 | 0.067692 | 5671 | LDL_size | Lipoprotein particle size |
| 23433 | 0.00126245 | 1.63E-04 | 5671 | HDL_size | Lipoprotein particle size |
| 23434 | 0.00096733 | 7.07E-03 | 5671 | Phosphoglycerides | Other lipids |
| 23436 | 0.0010819 | 2.20E-03 | 5671 | Cholines | Other lipids |
| 23437 | 0.00116025 | 1.07E-03 | 5671 | Phosphatidylc | Other lipids |
| 23438 | 0.00070807 | 0.0401308 | 5671 | Sphingomyelins | Other lipids |
| 23439 | -0.0001507 | 0.6867451 | 5671 | ApoB | Apolipoproteins |
| 23440 | 0.00171591 | 8.48E-07 | 5671 | ApoA1 | Apolipoproteins |
| 23442 | -6.233E-05 | 0.8720883 | 5671 | Total_FA | Fatty acids |
| 23443 | 0.00060079 | 0.1116253 | 5671 | Unsaturation | Fatty acids |
| 23444 | -7.06E-04 | 0.0612639 | 5671 | Omega_3 | Fatty acids |
| 23445 | 8.16E-04 | 0.0272138 | 5671 | Omega_6 | Fatty acids |
| 23446 | 4.95E-04 | 0.1794931 | 5671 | PUFA | Fatty acids |
| 23447 | -0.0005677 | 1.43E-01 | 5671 | MUFA | Fatty acids |
| 23448 | -0.0002013 | 0.5978568 | 5671 | SFA | Fatty acids |
| 23449 | 9.37E-04 | 0.010953 | 5671 | LA | Fatty acids |
| 23450 | -3.09E-04 | 0.4022959 | 5671 | DHA | Fatty acids |
| 23460 | -3.41E-04 | 0.3797411 | 5671 | Alanine | Amino acids |
| 23461 | 0.0005598 | 0.1486276 | 5671 | Glutamine | Amino acids |
| 23462 | 1.93E-04 | 0.6093766 | 5671 | Glycine | Amino acids |
| 23463 | 2.16E-05 | 0.9530654 | 5671 | Histidine | Amino acids |
| 23464 | -2.36E-04 | 0.5377821 | 5671 | Total_BCAA | Amino acids |
| 23465 | -0.0003841 | 0.3168948 | 5671 | Isoleucine | Amino acids |
| 23466 | 4.16E-05 | 0.9124561 | 5671 | Leucine | Amino acids |
| 23467 | -0.0002863 | 0.4528306 | 5671 | Valine | Amino acids |
| 23468 | 1.53E-04 | 0.7077672 | 5671 | Phenylalanine | Amino acids |
| 23469 | -4.22E-04 | 0.2681299 | 5671 | Tyrosine | Amino acids |
| 23470 | 2.90E-04 | 0.3603159 | 5671 | Glucose | Glycolysis related metabolites |
| 23471 | -2.38E-03 | 5.339E-10 | 5671 | Lactate | Glycolysis related metabolites |
| 23472 | -2.09E-03 | 6.536E-08 | 5671 | Pyruvate | Glycolysis related metabolites |
| 23473 | 0.00029985 | 4.50E-01 | 5671 | Citrate | Glycolysis related metabolites |
| 23474 | -3.47E-04 | 0.3831708 | 5671 | bOHbutyrate | Ketone bodies |
| 23475 | -4.61E-04 | 0.2962409 | 5671 | Acetate | Ketone bodies |
| 23476 | 1.53E-05 | 0.9680456 | 5671 | Acetoacetate | Ketone bodies |
| 23477 | 1.24E-04 | 0.7557337 | 5671 | Acetone | Ketone bodies |
| 23478 | -2.43E-05 | 0.9534249 | 5671 | Creatinine | Fluid balance |
| 23479 | 0.00030391 | 0.4338602 | 5671 | Albumin | Fluid balance |
| 23480 | -0.0013566 | 2.97E-04 | 5671 | GlycA | Inflammation |
| 23481 | -0.0010676 | 4.51E-03 | 5671 | XXL_VLDL_P | Chylomicrons and Extremely Large VLDL |
| 23482 | -0.0011178 | 2.86E-03 | 5671 | XXL_VLDL_L | Chylomicrons and Extremely Large VLDL |
| 23483 | -0.0011049 | 3.22E-03 | 5671 | XXL_VLDL_PL | Chylomicrons and Extremely Large VLDL |
| 23484 | -0.0010678 | 4.78E-03 | 5671 | XXL_VLDL_C | Chylomicrons and Extremely Large VLDL |
| 23485 | -0.0010428 | 6.06E-03 | 5671 | XXL_VLDL_CE | Chylomicrons and Extremely Large VLDL |
| 23486 | -0.0010609 | 4.91E-03 | 5671 | XXL_VLDL_FC | Chylomicrons and Extremely Large VLDL |
| 23487 | -1.07E-03 | 0.0042586 | 5671 | XXL_VLDL_TG | Chylomicrons and Extremely Large VLDL |
| 23488 | -0.0011006 | 3.78E-03 | 5671 | XL_VLDL_P | Very Large VLDL |
| 23489 | -0.001115 | 3.27E-03 | 5671 | XL_VLDL_L | Very Large VLDL |
| 23490 | -0.0011418 | 0.0026877 | 5671 | XL_VLDL_PL | Very Large VLDL |
| 23491 | -0.001055 | 6.11E-03 | 5671 | XL_VLDL_C | Very Large VLDL |
| 23492 | -0.0009561 | 1.34E-02 | 5671 | XL_VLDL_CE | Very Large VLDL |
| 23493 | -0.001115 | 3.54E-03 | 5671 | XL_VLDL_FC | Very Large VLDL |
| 23494 | -0.0010693 | 4.55E-03 | 5671 | XL_VLDL_TG | Very Large VLDL |
| 23495 | -0.0010261 | 7.56E-03 | 5671 | L_VLDL_P | Large VLDL |
| 23496 | -0.0010082 | 8.60E-03 | 5671 | L_VLDL_L | Large VLDL |
| 23497 | -0.0010901 | 0.0044362 | 5671 | L_VLDL_PL | Large VLDL |
| 23498 | -0.0010148 | 8.88E-03 | 5671 | L_VLDL_C | Large VLDL |
| 23499 | -0.0009212 | 1.84E-02 | 5671 | L_VLDL_CE | Large VLDL |
| 23500 | -0.0010664 | 5.44E-03 | 5671 | L_VLDL_FC | Large VLDL |
| 23501 | -0.0009093 | 1.76E-02 | 5671 | L_VLDL_TG | Large VLDL |
| 23502 | -0.000459 | 2.35E-01 | 5671 | M_VLDL_P | Medium VLDL |
| 23503 | -0.0005788 | 1.35E-01 | 5671 | M_VLDL_L | Medium VLDL |
| 23504 | -0.000442 | 2.50E-01 | 5671 | M_VLDL_PL | Medium VLDL |
| 23505 | -4.865E-05 | 0.8955904 | 5671 | M_VLDL_C | Medium VLDL |
| 23506 | 1.74E-04 | 0.631901 | 5671 | M_VLDL_CE | Medium VLDL |
| 23507 | -0.0003374 | 0.375585 | 5671 | M_VLDL_FC | Medium VLDL |
| 23508 | -0.0008009 | 3.92E-02 | 5671 | M_VLDL_TG | Medium VLDL |
| 23509 | -0.0008071 | 3.98E-02 | 5671 | S_VLDL_P | Small VLDL |
| 23510 | -0.0007705 | 4.92E-02 | 5671 | S_VLDL_L | Small VLDL |
| 23511 | -0.0005221 | 1.78E-01 | 5671 | S_VLDL_PL | Small VLDL |
| 23512 | -0.0005868 | 1.28E-01 | 5671 | S_VLDL_C | Small VLDL |
| 23513 | -0.0007447 | 5.53E-02 | 5671 | S_VLDL_CE | Small VLDL |
| 23514 | -0.0003064 | 0.4216254 | 5671 | S_VLDL_FC | Small VLDL |
| 23515 | -0.0008764 | 2.46E-02 | 5671 | S_VLDL_TG | Small VLDL |
| 23516 | -0.0004525 | 0.2253548 | 5671 | XS_VLDL_P | Very Small VLDL |
| 23517 | -0.0004252 | 0.2507849 | 5671 | XS_VLDL_L | Very Small VLDL |
| 23518 | -0.0006621 | 0.0776151 | 5671 | XS_VLDL_PL | Very Small VLDL |
| 23519 | -5.87E-05 | 0.8693569 | 5671 | XS_VLDL_C | Very Small VLDL |
| 23520 | 9.27E-05 | 0.792107 | 5671 | XS_VLDL_CE | Very Small VLDL |
| 23521 | -0.0004219 | 0.2521298 | 5671 | XS_VLDL_FC | Very Small VLDL |
| 23522 | -0.0009539 | 1.50E-02 | 5671 | XS_VLDL_TG | Very Small VLDL |
| 23523 | 3.08E-05 | 0.9322217 | 5671 | IDL_P | IDL |
| 23524 | 4.91E-04 | 0.1587657 | 5671 | IDL_L | IDL |
| 23525 | 3.76E-04 | 0.27956 | 5671 | IDL_PL | IDL |
| 23526 | 0.00063296 | 0.067571 | 5671 | IDL_C | IDL |
| 23527 | 0.00068236 | 0.0487958 | 5671 | IDL_CE | IDL |
| 23528 | 0.00047956 | 0.1688903 | 5671 | IDL_FC | IDL |
| 23529 | -0.0008203 | 0.0359752 | 5671 | IDL_TG | IDL |
| 23530 | -2.911E-05 | 0.9375679 | 5671 | L_LDL_P | Large LDL |
| 23531 | 4.80E-04 | 0.1899923 | 5671 | L_LDL_L | Large LDL |
| 23532 | 4.31E-04 | 0.2404065 | 5671 | L_LDL_PL | Large LDL |
| 23533 | 5.75E-04 | 0.1146168 | 5671 | L_LDL_C | Large LDL |
| 23534 | 5.27E-04 | 0.1521289 | 5671 | L_LDL_CE | Large LDL |
| 23535 | 6.91E-04 | 0.0528779 | 5671 | L_LDL_FC | Large LDL |
| 23536 | -0.0007811 | 0.0459569 | 5671 | L_LDL_TG | Large LDL |
| 23537 | -0.000177 | 0.6439516 | 5671 | M_LDL_P | Medium LDL |
| 23538 | 2.4154E-05 | 0.9495643 | 5671 | M_LDL_L | Medium LDL |
| 23539 | 6.4738E-05 | 0.8655003 | 5671 | M_LDL_PL | Medium LDL |
| 23540 | 7.5802E-05 | 0.8421397 | 5671 | M_LDL_C | Medium LDL |
| 23541 | -6.75E-05 | 0.860077 | 5671 | M_LDL_CE | Medium LDL |
| 23542 | 4.55E-04 | 0.2230277 | 5671 | M_LDL_FC | Medium LDL |
| 23543 | -0.0008249 | 3.48E-02 | 5671 | M_LDL_TG | Medium LDL |
| 23544 | -0.0003009 | 0.4269119 | 5671 | S_LDL_P | Small LDL |
| 23545 | -8.675E-05 | 0.8184854 | 5671 | S_LDL_L | Small LDL |
| 23546 | -9.212E-05 | 0.806532 | 5671 | S_LDL_PL | Small LDL |
| 23547 | 2.0085E-05 | 0.957508 | 5671 | S_LDL_C | Small LDL |
| 23548 | -0.0001065 | 0.7786757 | 5671 | S_LDL_CE | Small LDL |
| 23549 | 3.49E-04 | 0.3506954 | 5671 | S_LDL_FC | Small LDL |
| 23550 | -0.0009222 | 1.74E-02 | 5671 | S_LDL_TG | Small LDL |
| 23551 | 0.0009993 | 3.27E-03 | 5671 | XL_HDL_P | Very Large HDL |
| 23552 | 0.0010315 | 2.28E-03 | 5671 | XL_HDL_L | Very Large HDL |
| 23553 | 0.00099273 | 3.69E-03 | 5671 | XL_HDL_PL | Very Large HDL |
| 23554 | 0.00107869 | 1.46E-03 | 5671 | XL_HDL_C | Very Large HDL |
| 23555 | 0.00117882 | 4.83E-04 | 5671 | XL_HDL_CE | Very Large HDL |
| 23556 | 0.0006426 | 0.0658829 | 5671 | XL_HDL_FC | Very Large HDL |
| 23557 | -2.87E-04 | 0.4593488 | 5671 | XL_HDL_TG | Very Large HDL |
| 23558 | 0.00148628 | 1.09E-05 | 5671 | L_HDL_P | Large HDL |
| 23559 | 0.00157633 | 1.84E-06 | 5671 | L_HDL_L | Large HDL |
| 23560 | 0.00154389 | 4.35E-06 | 5671 | L_HDL_PL | Large HDL |
| 23561 | 0.00156799 | 2.49E-06 | 5671 | L_HDL_C | Large HDL |
| 23562 | 0.00158864 | 2.09E-06 | 5671 | L_HDL_CE | Large HDL |
| 23563 | 0.00140873 | 2.78E-05 | 5671 | L_HDL_FC | Large HDL |
| 23564 | 2.40E-04 | 0.5290568 | 5671 | L_HDL_TG | Large HDL |
| 23565 | 1.74E-03 | 1.117E-06 | 5671 | M_HDL_P | Medium HDL |
| 23566 | 0.00168221 | 2.44E-06 | 5671 | M_HDL_L | Medium HDL |
| 23567 | 0.00155267 | 1.75E-05 | 5671 | M_HDL_PL | Medium HDL |
| 23568 | 0.00185975 | 1.51E-07 | 5671 | M_HDL_C | Medium HDL |
| 23569 | 0.00188397 | 1.25E-07 | 5671 | M_HDL_CE | Medium HDL |
| 23570 | 0.00170848 | 1.10E-06 | 5671 | M_HDL_FC | Medium HDL |
| 23571 | -0.0004703 | 0.23224 | 5671 | M_HDL_TG | Medium HDL |
| 23572 | 0.00102194 | 0.0086045 | 5671 | S_HDL_P | Small HDL |
| 23573 | 0.00087956 | 0.0227918 | 5671 | S_HDL_L | Small HDL |
| 23574 | 0.00089191 | 0.0204185 | 5671 | S_HDL_PL | Small HDL |
| 23575 | 0.00119337 | 0.0021283 | 5671 | S_HDL_C | Small HDL |
| 23576 | 0.00118416 | 0.002386 | 5671 | S_HDL_CE | Small HDL |
| 23577 | 0.00105559 | 0.0056329 | 5671 | S_HDL_FC | Small HDL |
| 23578 | -0.0010433 | 6.34E-03 | 5671 | S_HDL_TG | Small HDL |
| 30000 | -0.0006655 | 4.01E-02 | 6555 | White blood cell (leukocyte) count | White blood cell |
| 30010 | -0.0005414 | 5.28E-02 | 6555 | Red blood cell (erythrocyte) count | Red blood cell |
| 30020 | -2.41E-04 | 0.3573245 | 6556 | Haemoglobin concentration | Red blood cell |
| 30030 | -2.60E-04 | 0.3325935 | 6556 | Haematocrit percentage | Red blood cell |
| 30040 | 0.00054725 | 7.81E-02 | 6556 | Mean corpuscular volume | Red blood cell |
| 30050 | 0.00043342 | 1.53E-01 | 6556 | Mean corpuscular haemoglobin | Red blood cell |
| 30060 | -5.19E-06 | 0.9862572 | 6556 | Mean corpuscular haemoglobin concentration | Red blood cell |
| 30070 | -3.89E-05 | 0.9030429 | 6556 | Red blood cell (erythrocyte) distribution width | Red blood cell |
| 30080 | 1.17E-04 | 0.7076553 | 6556 | Platelet count | Platelet |
| 30090 | -9.60E-05 | 0.7883132 | 4757 | Platelet crit | Platelet |
| 30100 | -3.32E-04 | 0.3133647 | 6555 | Mean platelet (thrombocyte) volume | Platelet |
| 30110 | -1.85E-04 | 0.6291689 | 4757 | Platelet distribution width | Platelet |
| 30120 | -0.0006159 | 0.056956 | 6550 | Lymphocyte count | White blood cell |
| 30130 | -0.0004235 | 1.91E-01 | 6550 | Monocyte count | White blood cell |
| 30140 | -0.0004398 | 1.74E-01 | 6550 | Neutrophill count | White blood cell |
| 30150 | -0.000647 | 0.0393949 | 6550 | Eosinophill count | White blood cell |
| 30160 | -4.64E-04 | 0.1575887 | 6550 | Basophill count | White blood cell |
| 30170 | -1.34E-04 | 0.7024152 | 4747 | Nucleated red blood cell count | Red blood cell |
| 30180 | -0.0001706 | 0.5963197 | 6551 | Lymphocyte percentage | White blood cell |
| 30190 | -1.06E-04 | 0.7492849 | 6551 | Monocyte percentage | White blood cell |
| 30200 | 1.71E-04 | 0.6029535 | 6551 | Neutrophill percentage | White blood cell |
| 30210 | -3.02E-04 | 0.3537398 | 6551 | Eosinophill percentage | White blood cell |
| 30220 | -1.58E-04 | 0.6326322 | 6551 | Basophill percentage | White blood cell |
| 30230 | -1.49E-04 | 0.6744209 | 4747 | Nucleated red blood cell percentage | Red blood cell |
| 30240 | -9.35E-04 | 0.0032226 | 6529 | Reticulocyte percentage | Red blood cell |
| 30250 | -0.0009568 | 0.0021125 | 6529 | Reticulocyte count | Red blood cell |
| 30260 | 0.00059759 | 0.0647523 | 6529 | Mean reticulocyte volume | Red blood cell |
| 30270 | 0.00072973 | 5.12E-02 | 4755 | Mean sphered cell volume | Red blood cell |
| 30280 | -0.0012744 | 5.51E-04 | 4755 | Immature reticulocyte fraction | Red blood cell |
| 30290 | -0.0010932 | 2.63E-03 | 4755 | High light scatter reticulocyte percentage | Red blood cell |
| 30300 | -0.0010547 | 3.64E-03 | 4755 | High light scatter reticulocyte count | Red blood cell |
| 30600 | -2.75E-05 | 0.9364784 | 5236 | Albumin | Liver function |
| 30610 | -0.0006883 | 0.0397346 | 5995 | Alkaline phosphatase | Bone and joint |
| 30620 | -6.66E-05 | 0.8350799 | 5995 | Alanine aminotransferase | Liver function |
| 30630 | 0.00126842 | 4.26E-05 | 5190 | Apolipoprotein A | Immunometabolism |
| 30640 | -0.0002889 | 3.84E-01 | 5973 | Apolipoprotein B | Immunometabolism |
| 30650 | 0.00157921 | 4.98E-06 | 5972 | Aspartate aminotransferase | Liver function |
| 30660 | 0.00129301 | 0.0001903 | 5065 | Direct bilirubin | Liver function |
| 30670 | 0.00050219 | 1.19E-01 | 5990 | Urea | Renal function |
| 30680 | 1.42E-04 | 0.6927363 | 5236 | Calcium | Bone and joint |
| 30690 | 2.70E-04 | 0.3821308 | 5998 | Cholesterol | Immunometabolism |
| 30700 | -3.77E-04 | 0.1545222 | 5987 | Creatinine | Renal function |
| 30710 | -0.0006534 | 0.0393418 | 5986 | C-reactive protein | Immunometabolism |
| 30720 | -0.0011762 | 4.02E-05 | 5988 | Cystatin C | Renal function |
| 30730 | -3.21E-04 | 0.3100676 | 5995 | Gamma glutamyltransferase | Liver function |
| 30740 | 6.44E-06 | 0.9802923 | 5235 | Glucose | Endocrine |
| 30750 | -0.0004248 | 0.1400723 | 4689 | Glycated haemoglobin (HbA1c) | Immunometabolism |
| 30760 | 0.00150797 | 3.41E-07 | 5235 | HDL cholesterol | Immunometabolism |
| 30770 | -5.13E-05 | 0.8771011 | 5936 | IGF-1 | Endocrine |
| 30780 | -0.0001218 | 0.7035228 | 5991 | LDL direct | Immunometabolism |
| 30790 | -5.33E-04 | 0.1584258 | 4782 | Lipoprotein A | Endocrine |
| 30800 | 0.00082522 | 0.2695266 | 659 | Oestradiol | Endocrine |
| 30810 | -1.29E-04 | 0.7052004 | 5220 | Phosphate | Renal function |
| 30820 | 0.00047604 | 0.6352832 | 576 | Rheumatoid factor | Immunometabolism |
| 30830 | 0.00109535 | 4.26E-04 | 5151 | SHBG | Endocrine |
| 30840 | 0.00137232 | 3.259E-05 | 5963 | Total bilirubin | Liver function |
| 30850 | 0.00028037 | 7.17E-02 | 5369 | Testosterone | Endocrine |
| 30860 | -3.10E-04 | 0.3785248 | 5234 | Total protein | Immunometabolism |
| 30870 | -0.0013552 | 4.03E-05 | 5987 | Triglycerides | Immunometabolism |
| 30880 | -2.94E-04 | 0.2835463 | 5985 | Urate | Renal function |
| 30890 | 0.00173861 | 4.00E-07 | 5726 | Vitamin D | Bone and joint |

| **Table S14. Association of circadian rest-activity with the risk of T2D outcomes by excluding events that occurred within the first year of follow-up (N = 73,964) ^a^** | | | | |
| --- | --- | --- | --- | --- |
| **Subgroup** | **Circadian rest-activity characteristics** | | | ***P* for trend ^c^** |
| **Amplitude** | High | Intermediate | Low |  |
| No. of events | 371 | 489 | 731 |  |
| Person years | 197,648 | 209,431 | 168,594 |  |
| Incidence per 1000 PYs | 1.88 | 2.33 | 4.34 |  |
| Model 1 | 1.00 (reference) | 1.17 (1.02, 1.33) ^b^ | 1.58 (1.39, 1.79) | <0.0001 |
| Model 2 | 1.00 (reference) | 1.15 (1.00, 1.31) | 1.46 (1.29, 1.66) | <0.0001 |
| Model 3 | 1.00 (reference) | 1.15 (1.00, 1.32) | 1.42 (1.25, 1.61) | <0.0001 |
| Model 4 | 1.00 (reference) | 1.16 (1.01, 1.32) | 1.45 (1.27, 1.64) | <0.0001 |
| **Acrophase** | Advanced | Intermediate | Delayed |  |
| No. of events | 373 | 965 | 253 |  |
| Person years | 116,495 | 376,706 | 82,472 |  |
| Incidence per 1000 PYs | 3.20 | 2.56 | 3.07 |  |
| Model 1 | 1.00 (reference) | 0.99 (0.88, 1.12) | 1.20 (1.02, 1.40) | 0.06 |
| Model 2 | 1.00 (reference) | 1.05 (0.93, 1.19) | 1.22 (1.04, 1.43) | 0.03 |
| Model 3 | 1.00 (reference) | 1.07 (0.95, 1.21) | 1.23 (1.05, 1.45) | 0.02 |
| Model 4 | 1.00 (reference) | 1.10 (0.97, 1.24) | 1.28 (1.09, 1.51) | <0.01 |
| **Pseudo-F** | High | Intermediate | Low |  |
| No. of events | 479 | 505 | 607 |  |
| Person years | 200,877 | 178,685 | 196,111 |  |
| Incidence per 1000 PYs | 2.38 | 2.83 | 3.10 |  |
| Model 1 | 1.00 (reference) | 1.09 (0.97, 1.24) | 1.17 (1.04, 1.32) | 0.01 |
| Model 2 | 1.00 (reference) | 1.09 (0.97, 1.24) | 1.17 (1.03, 1.32) | 0.01 |
| Model 3 | 1.00 (reference) | 1.08 (0.95, 1.23) | 1.14 (1.01, 1.29) | 0.03 |
| Model 4 | 1.00 (reference) | 1.11 (0.98, 1.26) | 1.17 (1.04, 1.32) | 0.01 |
| **Mesor** | High | Intermediate | Low |  |
| No. of events | 428 | 423 | 740 |  |
| Person years | 221,694 | 182,283 | 171,696 |  |
| Incidence per 1000 PYs | 1.93 | 2.32 | 4.31 |  |
| Model 1 | 1.00 (reference) | 1.10 (0.97, 1.26) | 1.63 (1.44, 1.84) | <0.0001 |
| Model 2 | 1.00 (reference) | 1.09 (0.95, 1.25) | 1.50 (1.33, 1.70) | <0.0001 |
| Model 3 | 1.00 (reference) | 1.09 (0.95, 1.25) | 1.50 (1.33, 1.70) | <0.0001 |
| Model 4 | 1.00 (reference) | 1.10 (0.96, 1.26) | 1.54 (1.36, 1.74) | <0.0001 |
| Abbreviations: BMI, body mass index; PYs, person-years; T2D-PRS, type 2 diabetes-polygenic risk score. | | | | |
| ^a^ Obtained by using multivariable Cox regression model. | | | | |
| ^b^ Hazard ratios (95% confidence interval) (all such values). | | | | |
| ^c^ P for trend was calculated across quartiles using multivariable Cox regression models. | | | | |
| Model 1 was adjusted for age, sex, and BMI. | | | | |
| Model 2 was additionally adjusted for recruitment center, smoking status, drinking status, healthy diet score, educational level, Townsend deprivation index, shiftwork, physical activity, season of accelerometer wear, use of blood pressure-lowering medications, and use of cholesterol-lowering medications. | | | | |
| Model 3 was additionally adjusted for sleep efficiency and sleep duration. | | | | |
| Model 4 was additionally adjusted for T2D-PRS, first 10 principal components of ancestry, and genotype measurement batch. | | | | |

| **Table S15. Subgroup analysis of association between circadian rest-activity and the risk of T2D by age categories (N = 74,165) ^a^** | | | | | |
| --- | --- | --- | --- | --- | --- |
| **Subgroup** | **Circadian rest-activity characteristics** | | | ***P* for trend ^c^** | ***P* for interaction** |
| **Amplitude** | High | Intermediate | Low |  | 0.29 |
| **Age < 65 year** |  |  |  |  |  |
| No. of events | 319 | 437 | 584 |  |  |
| Person years | 174,937 | 179,166 | 132,165 |  |  |
| Incidence per 1000 PYs | 1.82 | 2.44 | 4.42 |  |  |
| Minimally adjusted model | 1.00 (reference) | 1.22 (1.05, 1.41) ^b^ | 1.47 (1.28, 1.69) | <0.0001 |  |
| Fully adjusted model | 1.00 (reference) | 1.22 (1.06, 1.42) | 1.48 (1.29, 1.71) | <0.0001 |  |
| **Age ≥ 65 years** |  |  |  |  |  |
| No. of events | 89 | 109 | 246 |  |  |
| Person years | 22,730 | 30,291 | 36,478 |  |  |
| Incidence per 1000 PYs | 3.92 | 3.60 | 6.74 |  |  |
| Minimally adjusted model | 1.00 (reference) | 0.98 (0.74, 1.30) | 1.46 (1.14, 1.86) | <0.001 |  |
| Fully adjusted model | 1.00 (reference) | 0.97 (0.74, 1.29) | 1.42 (1.11, 1.82) | <0.01 |  |
| **Acrophase** | Advanced | Intermediate | Delayed |  | 0.72 |
| **Age < 65 year** |  |  |  |  |  |
| No. of events | 290 | 835 | 215 |  |  |
| Person years | 91,889 | 322,252 | 72,127 |  |  |
| Incidence per 1000 PYs | 3.16 | 2.59 | 2.98 |  |  |
| Minimally adjusted model | 1.00 (reference) | 1.00 (0.87, 1.14) | 1.15 (0.96, 1.37) | 0.18 |  |
| Fully adjusted model | 1.00 (reference) | 1.11 (0.97, 1.27) | 1.20 (1.00, 1.44) | 0.04 |  |
| **Age ≥ 65 years** |  |  |  |  |  |
| No. of events | 128 | 252 | 64 |  |  |
| Person years | 24,626 | 54,516 | 10,357 |  |  |
| Incidence per 1000 PYs | 5.20 | 4.62 | 6.18 |  |  |
| Minimally adjusted model | 1.00 (reference) | 1.00 (0.80, 1.23) | 1.29 (0.96, 1.75) | 0.19 |  |
| Fully adjusted model | 1.00 (reference) | 1.07 (0.86, 1.33) | 1.44 (1.06, 1.95) | 0.04 |  |
| **Pseudo-F** | High | Intermediate | Low |  | 0.21 |
| **Age < 65 year** |  |  |  |  |  |
| No. of events | 384 | 406 | 550 |  |  |
| Person years | 166,733 | 149,432 | 170,103 |  |  |
| Incidence per 1000 PYs | 2.30 | 2.72 | 3.23 |  |  |
| Minimally adjusted model | 1.00 (reference) | 1.07 (0.93, 1.23) | 1.22 (1.07, 1.39) | <0.01 |  |
| Fully adjusted model | 1.00 (reference) | 1.09 (0.95, 1.25) | 1.22 (1.07, 1.39) | <0.01 |  |
| **Age ≥ 65 years** |  |  |  |  |  |
| No. of events | 157 | 151 | 136 |  |  |
| Person years | 34,177 | 29,277 | 26,045 |  |  |
| Incidence per 1000 PYs | 4.59 | 5.16 | 5.22 |  |  |
| Minimally adjusted model | 1.00 (reference) | 1.06 (0.85, 1.33) | 1.02 (0.81, 1.29) | 0.84 |  |
| Fully adjusted model | 1.00 (reference) | 1.06 (0.85, 1.33) | 1.03 (0.82, 1.30) | 0.78 |  |
| **Mesor** | High | Intermediate | Low |  | 0.22 |
| **Age < 65 year** |  |  |  |  |  |
| No. of events | 365 | 367 | 608 |  |  |
| Person years | 193,175 | 154,180 | 138,914 |  |  |
| Incidence per 1000 PYs | 1.89 | 2.38 | 4.38 |  |  |
| Minimally adjusted model | 1.00 (reference) | 1.15 (1.00, 1.33) | 1.69 (1.48, 1.93) | <0.0001 |  |
| Fully adjusted model | 1.00 (reference) | 1.13 (0.98, 1.31) | 1.56 (1.36, 1.78) | <0.0001 |  |
| **Age ≥ 65 years** |  |  |  |  |  |
| No. of events | 106 | 117 | 221 |  |  |
| Person years | 28,541 | 28,132 | 32,826 |  |  |
| Incidence per 1000 PYs | 3.71 | 4.16 | 6.73 |  |  |
| Minimally adjusted model | 1.00 (reference) | 1.12 (0.86, 1.46) | 1.52 (1.21, 1.93) | <0.001 |  |
| Fully adjusted model | 1.00 (reference) | 1.16 (0.89, 1.51) | 1.51 (1.20, 1.92) | <0.001 |  |
| Abbreviations: BMI, body mass index; T2D-PRS, type 2 diabetes-polygenic risk score; PYs, person-years. | | | | | |
| ^a^ Obtained by using multivariable Cox regression model. | | | | | |
| ^b^ Hazard ratios (95% confidence interval) (all such values). | | | | | |
| ^c^ P for trend was calculated across quartiles using multivariable Cox regression models. | | | | | |
| Minimally adjusted model was adjusted for age, sex, and BMI. | | | | | |
| Fully adjusted model was additionally adjusted for recruitment center, smoking status, drinking status, healthy diet score, educational level, Townsend deprivation index, shiftwork, physical activity, season of accelerometer wear, use of blood pressure-lowering medications, use of cholesterol-lowering medications, sleep efficiency, sleep duration, T2D-PRS, first 10 principal components of ancestry, and genotype measurement batch. | | | | | |

| **Table S16. Subgroup analysis of association between circadian rest-activity and the risk of T2D by sex categories (N = 74,165) ^a^** | | | | | |
| --- | --- | --- | --- | --- | --- |
| **Subgroup** | **Circadian rest-activity characteristics** | | | ***P* for trend ^c^** | ***P* for interaction** |
| **Amplitude** | High | Intermediate | Low |  | 0.37 |
| **Females** |  |  |  |  |  |
| No. of events | 148 | 249 | 316 |  |  |
| Person years | 106,630 | 130,879 | 91,105 |  |  |
| Incidence per 1000 PYs | 1.39 | 1.90 | 3.47 |  |  |
| Minimally adjusted model | 1.00 (reference) | 1.13 (0.92, 1.38) ^b^ | 1.50 (1.23, 1.83) | <0.0001 |  |
| Fully adjusted model | 1.00 (reference) | 1.11 (0.91, 1.36) | 1.33 (1.09, 1.63) | <0.0001 |  |
| **Males** |  |  |  |  |  |
| No. of events | 260 | 297 | 514 |  |  |
| Person years | 91,037 | 78,577 | 77,539 |  |  |
| Incidence per 1000 PYs | 2.86 | 3.78 | 6.63 |  |  |
| Minimally adjusted model | 1.00 (reference) | 1.22 (1.03, 1.44) | 1.70 (1.46, 1.98) | <0.0001 |  |
| Fully adjusted model | 1.00 (reference) | 1.20 (1.02, 1.42) | 1.56 (1.34, 1.82) | <0.0001 |  |
| **Acrophase** | Advanced | Intermediate | Delayed |  | 0.71 |
| **Females** |  |  |  |  |  |
| No. of events | 119 | 476 | 118 |  |  |
| Person years | 55,263 | 225,709 | 47,642 |  |  |
| Incidence per 1000 PYs | 2.15 | 2.11 | 2.48 |  |  |
| Minimally adjusted model | 1.00 (reference) | 1.10 (0.90, 1.34) | 1.23 (0.95, 1.59) | 0.12 |  |
| Fully adjusted model | 1.00 (reference) | 1.21 (0.99, 1.48) | 1.29 (1.00, 1.67) | <0.01 |  |
| **Males** |  |  |  |  |  |
| No. of events | 299 | 611 | 161 |  |  |
| Person years | 61,252 | 151,060 | 34,841 |  |  |
| Incidence per 1000 PYs | 4.88 | 4.04 | 4.62 |  |  |
| Minimally adjusted model | 1.00 (reference) | 0.95 (0.83, 1.10) | 1.17 (0.96, 1.42) | 0.05 |  |
| Fully adjusted model | 1.00 (reference) | 1.06 (0.92, 1.22) | 1.23 (1.02, 1.50) | 0.04 |  |
| **Pseudo-F** | High | Intermediate | Low |  | 0.13 |
| **Females** |  |  |  |  |  |
| No. of events | 252 | 224 | 237 |  |  |
| Person years | 123,551 | 103,025 | 102,038 |  |  |
| Incidence per 1000 PYs | 2.04 | 2.17 | 2.32 |  |  |
| Minimally adjusted model | 1.00 (reference) | 0.98 (0.81, 1.17) | 1.06 (0.89, 1.27) | 0.54 |  |
| Fully adjusted model | 1.00 (reference) | 0.96 (0.80, 1.15) | 1.03 (0.86, 1.23) | 0.75 |  |
| **Males** |  |  |  |  |  |
| No. of events | 289 | 333 | 449 |  |  |
| Person years | 77,359 | 75,685 | 94,110 |  |  |
| Incidence per 1000 PYs | 3.74 | 4.40 | 4.77 |  |  |
| Minimally adjusted model | 1.00 (reference) | 1.14 (0.98, 1.34) | 1.25 (1.08, 1.45) | <0.01 |  |
| Fully adjusted model | 1.00 (reference) | 1.18 (1.01, 1.38) | 1.27 (1.09, 1.47) | <0.01 |  |
| **Mesor** | High | Intermediate | Low |  | 0.33 |
| **Females** |  |  |  |  |  |
| No. of events | 175 | 218 | 320 |  |  |
| Person years | 121,008 | 112,147 | 95,458 |  |  |
| Incidence per 1000 PYs | 1.45 | 1.94 | 3.35 |  |  |
| Minimally adjusted model | 1.00 (reference) | 1.12 (0.92, 1.37) | 1.51 (1.26, 1.83) | <0.0001 |  |
| Fully adjusted model | 1.00 (reference) | 1.09 (0.90, 1.34) | 1.40 (1.16, 1.69) | <0.001 |  |
| **Males** |  |  |  |  |  |
| No. of events | 296 | 266 | 509 |  |  |
| Person years | 100,707 | 70,165 | 76,281 |  |  |
| Incidence per 1000 PYs | 2.94 | 3.79 | 6.67 |  |  |
| Minimally adjusted model | 1.00 (reference) | 1.16 (0.98, 1.37) | 1.74 (1.51, 2.01) | <0.0001 |  |
| Fully adjusted model | 1.00 (reference) | 1.17 (0.99, 1.38) | 1.64 (1.42, 1.90) | <0.0001 |  |
| Abbreviations: BMI, body mass index; T2D-PRS, type 2 diabetes mellitus-polygenic risk score; PYs, person-years. | | | | | |
| ^a^ Obtained by using multivariable Cox regression model. | | | | | |
| ^b^ Hazard ratios (95% confidence interval) (all such values). | | | | | |
| ^c^ P for trend was calculated across quartiles using multivariable Cox regression models. | | | | | |
| Minimally adjusted model was adjusted for age, sex, and BMI. | | | | | |
| Fully adjusted model was additionally adjusted for recruitment center, smoking status, drinking status, healthy diet score, educational level, Townsend deprivation index, shiftwork, physical activity, season of accelerometer wear, use of blood pressure-lowering medications, use of cholesterol-lowering medications, sleep efficiency, sleep duration, T2D-PRS, first 10 principal components of ancestry, and genotype measurement batch. | | | | | |

| **Table S17. Association of circadian rest-activity with the risk of T2D outcomes with missing data imputed by multiple imputation (N = 74,533) ^a^** | | | | |
| --- | --- | --- | --- | --- |
| **Subgroup** | **Circadian rest-activity characteristics** | | | ***P* for trend ^c^** |
| **Amplitude** | High | Intermediate | Low |  |
| No. of events | 409 | 548 | 841 |  |
| Person years | 198,406 | 210,452 | 169,802 |  |
| Incidence per 1000 PYs | 2.06 | 2.60 | 4.95 |  |
| Minimally adjusted model | 1.00 (reference) | 1.18 (1.04, 1.35) ^b^ | 1.64 (1.46, 1.85) | <0.0001 |
| Fully adjusted model | 1.00 (reference) | 1.17 (1.03, 1.33) | 1.49 (1.32, 1.69) | <0.0001 |
| **Acrophase** | Advanced | Intermediate | Delayed |  |
| No. of events | 419 | 1,096 | 283 |  |
| Person years | 116,949 | 378,548 | 83,164 |  |
| Incidence per 1000 PYs | 3.58 | 2.90 | 3.40 |  |
| Minimally adjusted model | 1.00 (reference) | 1.00 (0.90, 1.13) | 1.19 (1.02, 1.38) | <0.01 |
| Fully adjusted model | 1.00 (reference) | 1.11 (0.99, 1.25) | 1.26 (1.08, 1.46) | <0.01 |
| **Pseudo-F** | High | Intermediate | Low |  |
| No. of events | 546 | 562 | 690 |  |
| Person years | 202,169 | 179,472 | 197,020 |  |
| Incidence per 1000 PYs | 2.70 | 3.13 | 3.50 |  |
| Minimally adjusted model | 1.00 (reference) | 1.07 (0.95, 1.20) | 1.17 (1.04, 1.31) | <0.01 |
| Fully adjusted model | 1.00 (reference) | 1.08 (0.96, 1.22) | 1.17 (1.04, 1.30) | <0.01 |
| **Mesor** | High | Intermediate | Low |  |
| No. of events | 473 | 488 | 837 |  |
| Person years | 222,612 | 183,121 | 172,927 |  |
| Incidence per 1000 PYs | 2.12 | 2.66 | 4.84 |  |
| Minimally adjusted model | 1.00 (reference) | 1.15 (1.02, 1.31) | 1.66 (1.48, 1.86) | <0.0001 |
| Fully adjusted model | 1.00 (reference) | 1.14 (1.01, 1.30) | 1.55 (1.38, 1.74) | <0.0001 |
| Abbreviations: BMI, body mass index; PYs, person-years; T2D-PRS, type 2 diabetes-polygenic risk score. | | | | |
| ^a^ Obtained by using multivariable Cox regression model. | | | | |
| ^b^ Hazard ratios (95% confidence interval) (all such values). | | | | |
| ^c^ P for trend was calculated across quartiles using multivariable Cox regression models. | | | | |
| Minimally adjusted model was adjusted for age, sex, and BMI. | | | | |
| Fully adjusted model was additionally adjusted for recruitment center, smoking status, drinking status, healthy diet score, educational level, Townsend deprivation index, shiftwork, physical activity, season of accelerometer wear, use of blood pressure-lowering medications, use of cholesterol-lowering medications, sleep efficiency, sleep duration, T2D-PRS, first 10 principal components of ancestry, and genotype measurement batch. | | | | |

| **Table S18. Association of circadian rest-activity with the risk of T2D outcomes after excluding participants with shift work history (N = 57,961) ^a^** | | | | |
| --- | --- | --- | --- | --- |
| **Subgroup** | **Circadian rest-activity characteristics** | | | ***P* for trend ^c^** |
| **Amplitude** | High | Intermediate | Low |  |
| No. of events | 310 | 825 | 200 |  |
| Person years | 151,105 | 165,521 | 133,263 |  |
| Incidence per 1000 PYs | 2.05 | 4.98 | 1.50 |  |
| Minimally adjusted model | 1.00 (reference) | 1.15 (0.99, 1.33) b | 1.57 (1.36, 1.80) | <0.0001 |
| Fully adjusted model | 1.00 (reference) | 1.13 (0.98, 1.31) | 1.43 (1.24, 1.64) | <0.0001 |
| **Acrophase** | Advanced | Intermediate | Delayed |  |
| No. of events | 310 | 825 | 200 |  |
| Person years | 88,872 | 296,863 | 64,155 |  |
| Incidence per 1000 PYs | 3.49 | 2.78 | 3.12 |  |
| Minimally adjusted model | 1.00 (reference) | 0.98 (0.86, 1.12) | 1.18 (1.01, 1.35) | <0.01 |
| Fully adjusted model | 1.00 (reference) | 1.09 (0.95, 1.24) | 1.25 (1.06, 1.43) | <0.01 |
| **Pseudo-F** | High | Intermediate | Low |  |
| No. of events | 541 | 557 | 686 |  |
| Person years | 200,803 | 178,595 | 196,036 |  |
| Incidence per 1000 PYs | 2.69 | 3.12 | 3.5 |  |
| Minimally adjusted model | 1.00 (reference) | 1.08 (0.94, 1.24) | 1.18 (1.04, 1.35) | <0.01 |
| Fully adjusted model | 1.00 (reference) | 1.09 (0.95, 1.25) | 1.18 (1.03, 1.34) | <0.01 |
| **Mesor** | High | Intermediate | Low |  |
| No. of events | 350 | 370 | 615 |  |
| Person years | 170,559 | 143,466 | 135,864 |  |
| Incidence per 1000 PYs | 2.05 | 2.58 | 4.53 |  |
| Minimally adjusted model | 1.00 (reference) | 1.16 (1.00, 1.34) | 1.61 (1.41, 1.84) | <0.0001 |
| Fully adjusted model | 1.00 (reference) | 1.15 (0.99, 1.33) | 1.51 (1.32, 1.73) | <0.0001 |
| Abbreviations: BMI, body mass index; PYs, person-years; T2D-PRS, type 2 diabetes-polygenic risk score. | | | | |
| ^a^ Obtained by using multivariable Cox regression model. | | | | |
| ^b^ Hazard ratios (95% confidence interval) (all such values). | | | | |
| ^c^ P for trend was calculated across quartiles using multivariable Cox regression models. | | | | |
| Minimally adjusted model was adjusted for age, sex, and BMI. | | | | |
| Fully adjusted model was additionally adjusted for recruitment center, smoking status, drinking status, healthy diet score, educational level, Townsend deprivation index, physical activity, season of accelerometer wear, use of blood pressure-lowering medications, use of cholesterol-lowering medications, sleep efficiency, sleep duration, T2D-PRS, first 10 principal components of ancestry, and genotype measurement batch. | | | | |

| **Table S19. Association of circadian rest-activity with the risk of T2D outcomes after** **adjusting for mediating factors. ^a^** | | | | |
| --- | --- | --- | --- | --- |
| **Subgroup** | **Circadian rest-activity characteristics** | | | ***P* for trend ^c^** |
| **Amplitude** | High | Intermediate | Low |  |
| Fully adjusted model+ Vitamin D | 1.00 (reference) | 1.14 (1.00, 1.31) ^b^ | 1.41 (1.24, 1.61) | <0.0001 |
| Fully adjusted model+ HDL cholesterol | 1.00 (reference) | 1.11 (0.97, 1.28) | 1.40 (1.23, 1.60) | <0.0001 |
| Fully adjusted model+ apolipoprotein A | 1.00 (reference) | 1.13 (0.98, 1.29) | 1.42 (1.25, 1.62) | <0.0001 |
| Fully adjusted model+ three mediating factors | 1.00 (reference) | 1.08 (0.94, 1.25) | 1.36 (1.19, 1.55) | <0.0001 |
| **Acrophase** | Advanced | Intermediate | Delayed |  |
| Fully adjusted model+ Vitamin D | 1.00 (reference) | 1.11 (0.98, 1.25) | 1.23 (1.05, 1.44) | 0.01 |
| Fully adjusted model+ HDL cholesterol | 1.00 (reference) | 1.09 (0.96, 1.23) | 1.22 (1.03, 1.43) | 0.02 |
| Fully adjusted model+ apolipoprotein A | 1.00 (reference) | 1.09 (0.97, 1.23) | 1.23 (1.04, 1.45) | 0.02 |
| Fully adjusted model+ three mediating factors | 1.00 (reference) | 1.07 (0.95, 1.22) | 1.2 (1.01, 1.42) | 0.04 |
| **Pseudo-F** | High | Intermediate | Low |  |
| Fully adjusted model+ Vitamin D | 1.00 (reference) | 1.09 (0.96, 1.23) | 1.12 (1.01, 1.26) | 0.04 |
| Fully adjusted model+ HDL cholesterol | 1.00 (reference) | 1.06 (0.94, 1.2) | 1.11 (1.02, 1.25) | 0.03 |
| Fully adjusted model+ apolipoprotein A | 1.00 (reference) | 1.06 (0.94, 1.21) | 1.11 (1.01, 1.26) | 0.03 |
| Fully adjusted model+ three mediating factors | 1.00 (reference) | 1.06 (0.93, 1.2) | 1.10 (0.97, 1.24) | 0.07 |
| **Mesor** | High | Intermediate | Low |  |
| Fully adjusted model+ Vitamin D | 1.00 (reference) | 1.14 (0.99, 1.30) | 1.50 (1.33, 1.70) | <0.0001 |
| Fully adjusted model+ HDL cholesterol | 1.00 (reference) | 1.11 (0.97, 1.27) | 1.49 (1.32, 1.69) | <0.0001 |
| Fully adjusted model+ apolipoprotein A | 1.00 (reference) | 1.13 (0.98, 1.29) | 1.52 (1.34, 1.72) | <0.0001 |
| Fully adjusted model+ three mediating factors | 1.00 (reference) | 1.10 (0.95, 1.26) | 1.46 (1.29, 1.66) | <0.0001 |
| Abbreviations: BMI, body mass index; PYs, person-years; T2D-PRS, type 2 diabetes-polygenic risk score. | | | | |
| ^a^ Obtained by using multivariable Cox regression model. | | | | |
| ^b^ Hazard ratios (95% confidence interval) (all such values). | | | | |
| ^c^ P for trend was calculated across quartiles using multivariable Cox regression models. | | | | |
| Fully adjusted model was additionally adjusted for recruitment center, smoking status, drinking status, healthy diet score, educational level, Townsend deprivation index, physical activity, season of accelerometer wear, use of blood pressure-lowering medications, use of cholesterol-lowering medications, sleep efficiency, sleep duration, T2D-PRS, first 10 principal components of ancestry, and genotype measurement batch. | | | | |

| **Table S20. Association of circadian rest-activity with the risk of all-cause mortality among participants with T2D after adjusting for the duration of T2D ^a^** | | | | |
| --- | --- | --- | --- | --- |
| **Subgroup** | **Circadian rest-activity characteristics** | | | ***P* for trend ^c^** |
| **Amplitude** | High | Intermediate | Low |  |
| Fully adjusted model+ duration of T2D | 1.00 (reference) | 0.97 (0.74, 1.27) | 1.30 (1.03, 1.64) | <0.01 |
| **Acrophase** | Advanced | Intermediate | Delayed |  |
| Fully adjusted model+ duration of T2D | 1.00 (reference) | 1.03 (0.84, 1.26) | 1.25 (0.96, 1.62) | 0.13 |
| **Pseudo-F** | High | Intermediate | Low |  |
| No. of events | 1.00 (reference) | 1.00 (0.80, 1.24) | 1.04 (0.84, 1.28) | 0.71 |
| **Mesor** | High | Intermediate | Low |  |
| No. of events | 1.00 (reference) | 0.95 (0.73, 1.23) | 1.27 (1.03, 1.58) | <0.01 |
| Abbreviations: BMI, body mass index; PYs, person-years, T2D, type 2 diabetes. | | | | |
| ^a^ Obtained by using multivariable Cox regression model. | | | | |
| ^b^ Hazard ratios (95% confidence interval) (all such values). | | | | |
| ^c^ P for trend was calculated across quartiles using multivariable Cox regression models. | | | | |
| Fully adjusted model was additionally adjusted for recruitment center, smoking status, drinking status, healthy diet score, educational level, Townsend deprivation index, physical activity, season of accelerometer wear, use of blood pressure-lowering medications, use of cholesterol-lowering medications, sleep efficiency, sleep duration, T2D-PRS, first 10 principal components of ancestry, and genotype measurement batch. | | | | |

| **Table S21. Association of circadian rest-activity with the risk of T2D outcomes by using competing risk regression (Fine and Gray) (N = 74,165) ^a^** | | | | |
| --- | --- | --- | --- | --- |
| **Subgroup** | **Circadian rest-activity characteristics** | | | ***P* for trend ^c^** |
| **Amplitude** | High | Intermediate | Low |  |
| No. of events | 408 | 546 | 830 |  |
| Person years | 197,667 | 209,456 | 168,644 |  |
| Incidence per 1000 PYs | 2.06 | 2.61 | 4.92 |  |
| Minimally adjusted model | 1.00 (reference) | 1.18 (1.04, 1.35) ^b^ | 1.61 (1.42, 1.81) | <0.0001 |
| Fully adjusted model | 1.00 (reference) | 1.17 (1.03, 1.33) | 1.46 (1.29, 1.65) | <0.0001 |
| **Acrophase** | Advanced | Intermediate | Delayed |  |
| No. of events | 418 | 1,087 | 279 |  |
| Person years | 116,515 | 376,768 | 82,484 |  |
| Incidence per 1000 PYs | 3.59 | 2.89 | 3.38 |  |
| Minimally adjusted model | 1.00 (reference) | 1.00 (0.89, 1.12) | 1.18 (1.01, 1.37) | 0.07 |
| Fully adjusted model | 1.00 (reference) | 1.10 (0.98, 1.24) | 1.24 (1.06, 1.45) | <0.01 |
| **Pseudo-F** | High | Intermediate | Low |  |
| No. of events | 541 | 557 | 686 |  |
| Person years | 200,910 | 178,709 | 196,148 |  |
| Incidence per 1000 PYs | 2.69 | 3.12 | 3.50 |  |
| Minimally adjusted model | 1.00 (reference) | 1.07 (0.95, 1.20) | 1.17 (1.04, 1.31) | <0.01 |
| Fully adjusted model | 1.00 (reference) | 1.08 (0.96, 1.21) | 1.17 (1.04, 1.31) | <0.01 |
| **Mesor** | High | Intermediate | Low |  |
| No. of events | 471 | 484 | 829 |  |
| Person years | 221,716 | 182,312 | 171,740 |  |
| Incidence per 1000 PYs | 2.12 | 2.65 | 4.83 |  |
| Minimally adjusted model | 1.00 (reference) | 1.15 (1.01, 1.30) | 1.63 (1.45, 1.83) | <0.0001 |
| Fully adjusted model | 1.00 (reference) | 1.13 (1.00, 1.29) | 1.53 (1.36, 1.71) | <0.0001 |
| Abbreviations: BMI, body mass index; PYs, person-years; T2D-PRS, type 2 diabetes-polygenic risk score. | | | | |
| ^a^ Obtained by using multivariable Cox regression model. | | | | |
| ^b^ Hazard ratios (95% confidence interval) (all such values). | | | | |
| ^c^ P for trend was calculated across quartiles using multivariable Cox regression models. | | | | |
| Minimally adjusted model was adjusted for age, sex, and BMI. | | | | |
| Fully adjusted model was additionally adjusted for recruitment center, smoking status, drinking status, healthy diet score, educational level, Townsend deprivation index, shiftwork, physical activity, season of accelerometer wear, use of blood pressure-lowering medications, use of cholesterol-lowering medications, sleep efficiency, sleep duration, T2D-PRS, first 10 principal components of ancestry, and genotype measurement batch. | | | | |

Participants with acceleration intensity time- series data

(n = 103,712)

Participants who did not undergo health examinations during follow-up were excluded (n=3,294)

Participants included in the final follow-up analysis (n=15

Participants who directed to hypertension were excluded (n=1,056)

Participants with unreliable accelerometry data

(n = 11,104)

Participants with incomplete information on any variables (n = 538)

Participants with prevalent diabetes at baseline

(n = 3,248)

Participants with without complete genetic data or not of European descent (n =14,657)

Participants included in the T2D incidence analysis

(n = 74,165)

Blood biomarkers analysis (n = 64,321)

Metabolomics analysis (n = 40,978)

Participants included in the all-cause mortality among participants T2D analysis (n = 4,551)

**Figure S1.** Selection of study participants in the UK Biobank Cohort.

**Figure S2.** Restricted cubic spline models for the association between (A) amplitude, (B) acrophase, (C) Pseudo-F, (D) mesor and risk of all-cause mortality among participants with type 2 diabetes. The 95% CIs of the adjusted HRs are represented by the shaded are. Restricted cubic spline model is adjusted for age, sex, BMI, recruitment center, smoking status, drinking status, healthy diet score, educational level, Townsend deprivation index, shiftwork, physical activity, season of accelerometer wear, use of blood pressure-lowering medications, use of cholesterol-lowering medications, sleep efficiency, sleep duration, type 2 diabetes polygenic risk score, first 10 principal components of ancestry, and genotype measurement batch.


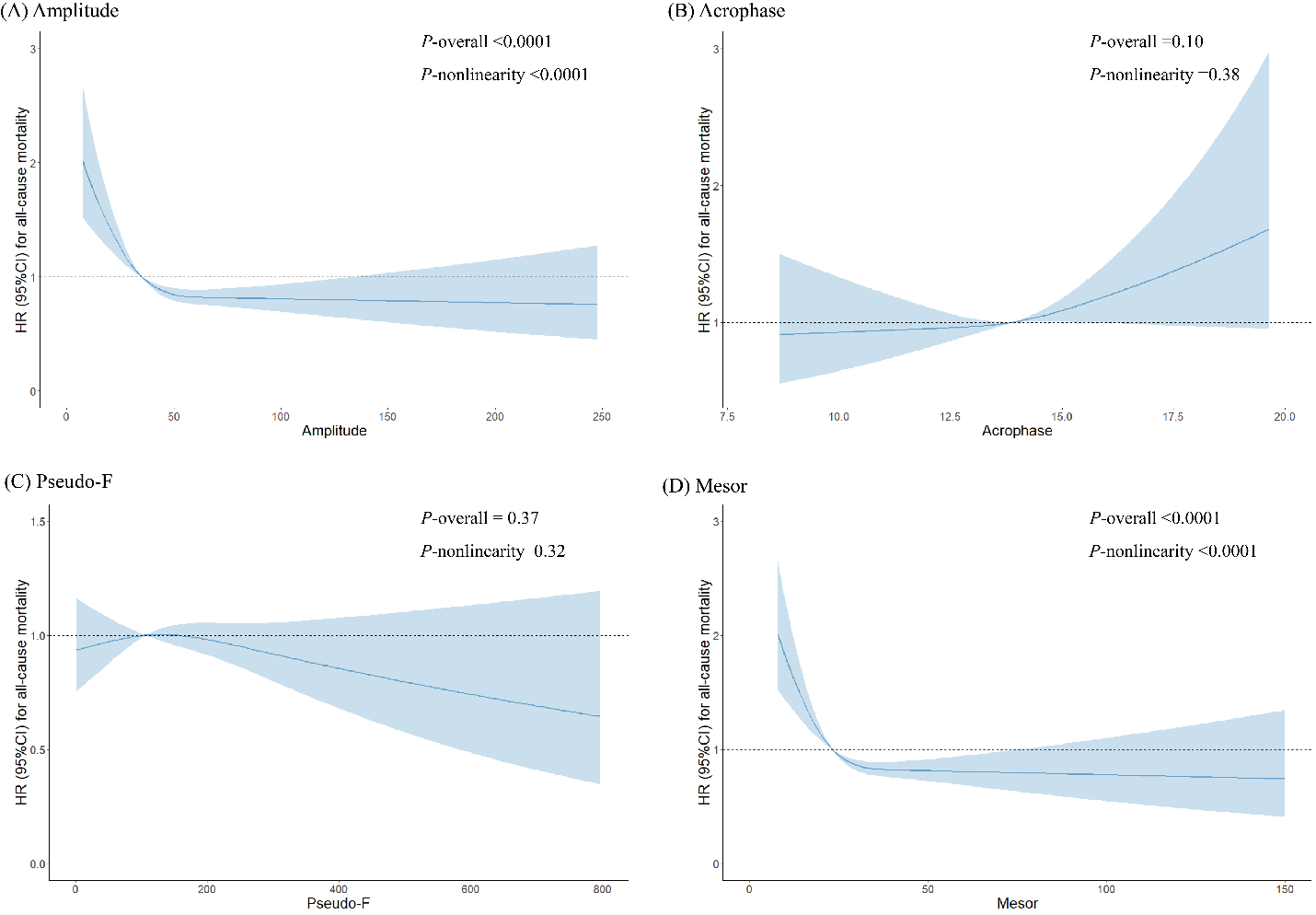

Supplement: Supplementary file 1 — Supplemental material [file 41387_2025_395_MOESM1_ESM.docx]
